# Diversity of *Harbinger*-like Transposons in Teleost Fish Genomes

**DOI:** 10.3390/ani12111429

**Published:** 2022-05-31

**Authors:** Ema Etchegaray, Corentin Dechaud, Jérémy Barbier, Magali Naville, Jean-Nicolas Volff

**Affiliations:** Institut de Génomique Fonctionnelle de Lyon, Ecole Normale Supérieure de Lyon, CNRS UMR 5242, Université Claude Bernard Lyon 1, 69007 Lyon, France; ema.etchegaray@ens-lyon.fr (E.E.); corentin.dechaud@ens-lyon.fr (C.D.); jeremy.barbier@ens-lyon.fr (J.B.); magali.naville@gmail.com (M.N.)

**Keywords:** transposable elements, *Harbinger*, *ISL2EU*, genomes, teleost fish, evolution

## Abstract

**Simple Summary:**

The study of transposable elements, which are repeated DNA sequences that can insert into new locations in genomes, is of particular interest to genome evolution, as they are sources of mutations but also of new regulatory and coding sequences. Teleost fish are a species-rich clade presenting a high diversity of transposable elements, both quantitatively and qualitatively, making them a very attractive group to investigate the evolution of mobile sequences. We studied *Harbinger*-like DNA transposons, which are widespread from plants to vertebrates but absent from mammalian genomes. These elements code for both a transposase and a *Myb*-like protein. We observed high variability in the genomic composition of *Harbinger*-like sequences in teleost fish. While *Harbinger* transposons might have been present in a common ancestor of all the fish species studied, *ISL2EU* elements were possibly gained by horizontal transfer at the base of teleost fish. Transposase and *Myb*-like protein phylogenies of *Harbinger* transposons indicated unique origins of the association between both genes and suggests recombination was rare between transposon sublineages. Finally, we report one case of *Harbinger* horizontal transfer between divergent fish species and the transcriptional activity of both *Harbinger* and *ISL2EU* transposons in teleost fish. There was male-biased expression in the gonads of the medaka fish.

**Abstract:**

*Harbinger* elements are DNA transposons that are widespread from plants to vertebrates but absent from mammalian genomes. Among vertebrates, teleost fish are the clade presenting not only the largest number of species but also the highest diversity of transposable elements, both quantitatively and qualitatively, making them a very attractive group to investigate the evolution of mobile sequences. We studied *Harbinger* DNA transposons and the distantly related *ISL2EU* elements in fish, focusing on representative teleost species compared to the spotted gar, the coelacanth, the elephant shark and the amphioxus. We observed high variability in the genomic composition of *Harbinger*-like sequences in teleost fish, as they covered 0.002–0.14% of the genome, when present. While *Harbinger* transposons might have been present in a common ancestor of all the fish species studied here, with secondary loss in elephant shark, our results suggests that *ISL2EU* elements were gained by horizontal transfer at the base of teleost fish 200–300 million years ago, and that there was secondary loss in a common ancestor of pufferfishes and stickleback. *Harbinger* transposons code for a transposase and a *Myb*-like protein. We reconstructed and compared molecular phylogenies of both proteins to get insights into the evolution of *Harbinger* transposons in fish. Transposase and *Myb*-like protein phylogenies showed global congruent evolution, indicating unique origin of the association between both genes and suggesting rare recombination between transposon sublineages. Finally, we report one case of *Harbinger* horizontal transfer between divergent fish species and the transcriptional activity of both *Harbinger* and *ISL2EU* transposons in teleost fish. There was male-biased expression in the gonads of the medaka fish.

## 1. Introduction

Transposable elements (TEs) are repeated DNA sequences that can be inserted into new locations in genomes. They are classified into two main classes, class I retrotransposons and class II DNA transposons, depending on their transposition mechanisms. A class I TE uses an RNA intermediate that is reverse-transcribed into a new cDNA copy of the element (copy-and-paste mechanism), whereas a class II transposon is generally excised from the original locus by a transposase and integrated into another site (cut-and-paste mechanism). Within each class, TEs are subdivided into superfamilies and families according to their phylogenetic relationships [1]. Since the discovery of TEs in the 1950s, TEs have been shown to be major components of genomes, and there is growing evidence of their important roles in genome evolution and organism adaptation [2,3,4,5,6].

With the flourishing development of sequencing technologies and genome annotation tools, the number of sequenced genomes has exploded. Studying new species besides model organisms allows a broader and wider understanding of the molecular basis and evolutionary dynamics of biodiversity. In particular, fish, of which there are 36,000 known species—more than 48% being vertebrate species—are still understudied [7,8]. Notably, the group of species called fish does not represent a monophyletic group, since it is composed of both chondrichthyes (cartilaginous fish) and osteichthyes, the latter comprising both bony fish and non-fish sarcopterygian species. Bony fish are subdivided into actinopterygians (ray-finned fish), including the non-teleost spotted gar and teleost fishes, such as zebrafish, cod, stickleback, tetraodon, fugu, platyfish, medaka and tilapia; and into sarcopterygians (lobe-finned fish) such as coelacanth and lungfish, which are the closest fish relatives of terrestrial vertebrates (tetrapods).

Teleost fish constitute the great majority of fish species. They present a high level of biodiversity and are considered to have plastic genomes [3]. They particularly constitute an attractive group for studying mobile sequences, since their genomes present a high level of TE diversity, both qualitatively and quantitatively [3,9,10]. Indeed, teleost fish genomes contain larger numbers of TE superfamilies compared to tetrapods, particularly birds and mammals, which have lost many groups of transposable elements during their evolution [3]. Teleost genomes are also variable in term of TE coverage, which ranges from less than 10% for tetraodon and fugu, which are species with compact genomes, to more than 50% for zebrafish [9]. Finally, fish genomes have higher proportions of class II DNA transposons compared to mammals and birds, which mainly possess class I TEs.

In this study, we investigated in teleost fish genomes a superfamily of DNA transposons called *Harbinger* or *PIF/Harbinger*. *Harbinger* transposons are found in various species, including fish, other animals and plants, but are absent from mammalian genomes [11,12,13,14,15,16,17,18,19,20,21,22,23]. They are usually flanked by terminal inverted repeats (TIRS) 25–50 base pairs (bp) in length and generally generate 3 bp long target site duplications through their integration into a genomic site. Typical autonomous *Harbinger* elements carry two open reading frames (ORFs) [16]. The first ORF codes for a transposase containing a DDE endonuclease motif, which is composed of three carboxylate residues coordinating metal ions necessary for both catalysis of DNA cleavage at the site of insertion and strand transfer. The second ORF encodes a DNA-binding protein possessing a conserved *Myb*/SANT-like domain (we will refer to this ORF as *Myb*-like). This domain is composed of a tri-helix motif—with conserved bulky aromatic residues essential for the stability of the motif—allowing interactions with DNA and proteins. Indeed, the *Myb*-like protein has been shown to interact with the transposase, thereby allowing their concomitant nuclear import, and to bind DNA at the *Harbinger* TIR sequences, leading to the excision of the *Harbinger* sequence by the transposase [24]. Transposases present high degrees of conservation even between different families of *Harbinger* transposons, whereas the *Myb*-like proteins are much more divergent, there being only some similarities in restricted parts of the *Myb*-like domain sequence between different *Harbinger* families [16].

*ISL2EU* elements are distantly related to *Harbinger* transposons but belong to the same superfamily called *Harbinger*-like [20,22]. They are found in animals and display two ORFs, one encoding a DDE transposase with helix–turn–helix (HTH) or THAP putative DNA-binding domains, and the other one coding for an exonuclease containing an YqaJ alkaline exonuclease domain. Therefore, even if *Harbinger* and *ISL2EU* elements both present two ORFs, one of them encoding a DDE transposase, the second ORF is different in the two types of elements. Finally, other *Harbinger*-like elements called *Spy* have been found only in invertebrates [22,25]. Most *Spy* elements present only one ORF encoding a transposase with DDE and HTH motifs.

Both genes of *Harbinger* transposons are necessary for transposition in vitro [24,26]. *Harbinger* elements are transcriptionally active in *Triticeae* plants and in the salamander *Pleurodeles waltl* [27,28]. In another study, the expression of a coelacanth *Harbinger* transposon was detected in a mouse PAC transgenic cell line (P1-derived artificial chromosome with a coelacanth genomic insert containing a *Harbinger* element), suggesting its expression in the coelacanth itself [29].

Here we present the analysis of the evolutionary dynamics of *Harbinger* and *ISL2EU* transposons, collectively called *Harbinger*-like elements, in teleost fish species for which genome assemblies of good quality are available. Genomes of spotted gar (*Lepisosteus oculatus*, non-teleost ray-finned fish), coelacanth (*Latimeria chalumnae*, sarcopterygian), elephant shark (*Callorhinchus milii*, cartilaginous fish) and amphioxus (*Branchiostoma floridae*, cephalochordate) were also included in this study as external groups for comparison. We observed a differential distribution of *Harbinger*-like elements depending on the fish genomes, including high abundance in medaka for *Harbinger* transposons and in platyfish for *ISL2EU* transposons. Moreover, we performed a comparative evolutionary analysis of the two ORFs of *Harbinger* transposons. We observed the persistence of the two ORFs in all fish transposons studied. There was global congruent evolution for the transposase and the *Myb*-like proteins. Finally, we also show evidence of transcriptional activity of *Harbinger* and *ISL2EU* transposons in fish, including testis-biased expression in the gonads of the medaka, and one probable case of horizontal transfer between divergent species.

## 2. Materials and Methods

### 2.1. Genomes

We used the following genome sequences in our analysis: amphioxus (Branchiostoma_floridae_v2.0.assembly.fasta, http://genome.jgi-psf.org/Brafl1/Brafl1.download.ftp.html, accessed on 30 January 2015), elephant shark (EsharkAssembly, http://esharkgenome.imcb.a-star.edu.sg, accessed on 30 January 2015), fugu (Takifugu_rubripes.FUGU4.66.dna.toplevel.fa, Ensembl), tetraodon (Tetraodon_nigroviridis.TETRAODON8.73.dna.toplevel.fa, Ensembl), stickleback (Gasterosteus_aculeatus.BROADS1.68.dna.toplevel.fa, Ensembl), Nile tilapia (Oreochromis_niloticus.Orenil1.0.68.dna.toplevel.fa, Ensembl), platyfish (Xiphophorus_maculatus.Xipmac4.4.2.69.dna.nonchromosomal.fa, Ensembl), medaka (https://www.ncbi.nlm.nih.gov/assembly/GCF_002234675.1, accessed on 15 September 2021), Atlantic cod (Gadus_morhua.gadMor1.73.dna.toplevel.fa, Ensembl), zebrafish (Danio_rerio.Zv9.66.dna.toplevel.fa, Ensembl), silver (Coho) salmon (Okis_V2, GCA_002021735.2, Ensembl), European (common) carp (Cypcar_WagV4.0, GCA_905221575.1, Ensembl), sea bass (seabass_V1.0, GCA_000689215.1, Ensembl), large yellow croaker (L_crocea_2.0, GCA_000972845.2, Ensembl), swamp eel (M_albus_1.0, GCA_001952655.1, Ensembl), mangrove killifish (ASM164957v1, GCA_001649575.1, Ensembl), spotted gar (http://www.ncbi.nlm.nih.gov/assembly/GCF_000242695.1/, Genbank Assembly, accessed on 30 January 2015) and African coelacanth (Latimeria_chalumnae.LatCha1.72.dna_toplevel.fa, Ensembl).

### 2.2. TE Annotation

TE libraries were established by a combination of both automatic and manual annotations. Manual annotations corresponded to TBLASTN searches against genomes (downloaded or on the NCBI website https://www.ncbi.nlm.nih.gov/genome/, accessed on 15 September 2021) using TE proteins from the *PIF-Harbinger* and *PIF-ISL2EU* superfamilies as queries, using default parameters without any filtering for low complexity regions. TE sequences were also retrieved from the Repbase database (http://www.girinst.org, accessed on 15 September 2021) [30]. Automatic annotation was performed using the RepeatModeler software (Smit, AFA, Hubley, R., http://www.repeatmasker.org, accessed on 15 September 2021) with default parameters. For the coelacanth, we used and reannotated the library from Amemiya et al. (2013) [31]. Predicted transposase and *Myb*-like sequences are provided as Supplementary Data S1 and S2, respectively.

### 2.3. TE Genome Masking, Copy Number and Genome Coverage

TE genome masking, copy number and genome coverage estimations were performed according to Chalopin et al. (2015) [9]. Briefly, RepeatMasker version 3.3.0 (Smit, AFA, Hubley, R, and Green, P. RepeatMasker Open-3.0. 1996–2010; http://www.repeatmasker.org, accessed on 15 September 2021) with “-a” and “-lib” default parameters was locally used to mask genomes. Copy number and genome coverage were calculated on RepeatMasker outfiles (.out) with custom scripts (Supplementary Data S3). In order to eliminate very short and too divergent sequences, data were filtered to include only elements longer than 80 nucleotides and sharing more than 80% of identity with the reference sequences from the species-specific library [9].

### 2.4. Sequence Alignment and Phylogenetic Analysis

Nucleotide and amino-acid sequences were aligned using MAFFT [32]. Phylogenetic trees were built using maximum likelihood calculation with the PhyML software [33] using the LG model, which is optimized for amino-acid alignment [34] (invariable sites optimized, tree searching operation with best of NNI and SPR, bootstraps with 100 replicates). Phylogenetic analysis was also performed with the MrBayes package [35] using the mixed model (estimated by the protest-3 software [36]), 500,000 generations of Bayesian inferences for the tree with consensus sequences and 5,000,000 for the tree with transposase copies to reach average standard deviation of split frequencies <0.02 (burn-in: 25%).

### 2.5. TE Expression Analysis in Spotted Gar, Zebrafish, Cod and Medaka

TE expression was studied by comparing TE consensus protein sequences against the PhyloFish database, which provides qualitative non-normalized expression tendencies [37]. Results were analyzed using the RNAbrowse interface (http://phylofish.sigenae.org/index.html, accessed on 15 January 2022) [38]. Expression was given at the copy level in PhyloFish and summarized for all superfamilies.

### 2.6. TE Expression Analysis in Medaka Gonads

TE expression quantification was performed on RNAseq data from both male and female gonads of the medaka fish *O. latipes*, as previously published [39]. In brief, SquIRE [40] was used to estimate TE expression at the copy level using a custom TE library on the *O. latipes* genome (https://www.ncbi.nlm.nih.gov/assembly/GCF_002234675.1, accessed on 15 September 2021). We ran SQuIRE “clean,” “map,” “count,” and “call” steps to estimate TE expression. Then, mean expression was calculated for all copies of a same TE family.

### 2.7. TE Distribution on Medaka Chromosomes

Distribution of *Harbinger* and *ISL2EU* elements along medaka chromosomes was represented using the R karyoploteR package (available at the Bioconductor site https://bioconductor.org/packages/release/bioc/html/karyoploteR.html, accessed on 15 January 2022).

## 3. Results

### 3.1. Differential Contributions of Harbinger-like Transposons to Fish Genomes

**Figure 1:**
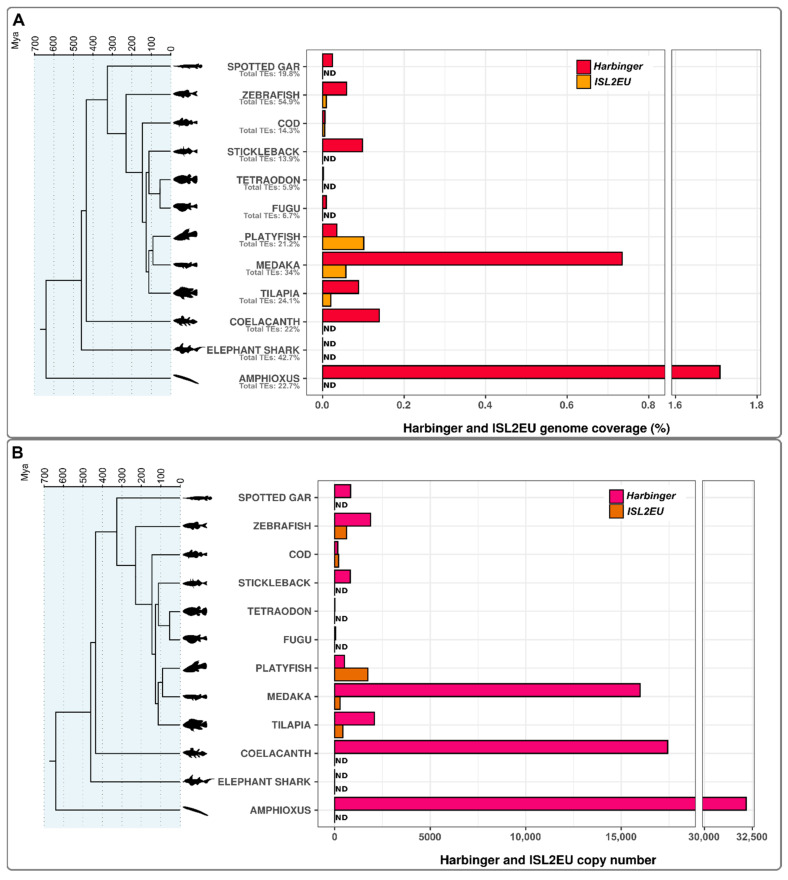
Genome coverage (**A**) and copy number (**B**) of *Harbinger* and *ISL2EU* transposons in spotted gar, zebrafish, cod, stickleback, tetraodon, fugu, platyfish, medaka, tilapia, coelacanth, elephant shark and amphioxus. Data were filtered to include only copies longer than 80 nucleotides and sharing more than 80% of identity with the reference sequence from the species-specific library [9]. In (**A**), the global percentage of TEs in genomes is indicated for each species under the species name. ND (not detected) is indicated when no element was detected in the species. Species phylogeny was based on divergence times estimated using the TimeTree public database [41].

In order to investigate the genomic contributions, evolution and diversification of *Harbinger* and *ISL2EU* transposons in fish genomes, we analyzed various teleost fish species, as along with one non-teleost ray-finned fish (spotted gar), one sarcopterygian fish (coelacanth), one cartilaginous fish (elephant shark) and one cephalochordate (amphioxus), which were used as outgroups for comparison (Figure 1).

*Harbinger* transposon content was variable in teleost fish genomes. Tetraodon, fugu and cod genomes, which contain the lowest global amounts of TEs among the species studied (5.9%, 6.7% and 14.3%, respectively [9]), also had the lowest genomic contributions of *Harbinger* transposons. The zebrafish genome, which otherwise possesses the highest global TE content among teleost fish (54.9% [9]), was not particularly enriched in *Harbinger* transposons, indicating that these elements did not significantly contribute to TE expansion in this fish. In contrast, *Harbinger* transposons were particularly well represented in medaka in terms of both genome coverage and copy number. Outside ray-finned fish, these transposons were present with high copy numbers in both coelacanth and amphioxus, this being, however, not correlated with high coverage in coelacanth, probably due to large genome size (more than 2800 Mb [31]). Finally, *Harbinger* elements could not be detected in elephant shark in our analysis.

*ISL2EU* elements were detected in teleost fish but absent from the non-teleost species, including spotted gar, coelacanth and elephant shark. Concerning amphioxus, Han et al. indicated the presence of ISL2EU in this genome; however, we were not able to detect them with our analysis [22]. Since such elements are also absent from tetrapods but present in more divergent animals [22], they might have been gained through horizontal transfer at the base of the teleost lineage 200–300 million years ago. Within teleost fish, *ISL2EU* distribution was patchy compared to *Harbinger*, with the absence of three out of the eight species studied for genome coverage (fugu, tetraodon and stickleback). This suggests secondary loss of the elements in a common ancestor of these three species about 100 million years ago. *ISL2EU* elements were particularly present in platyfish and medaka genomes. Overall, there was no clear correlation between *Harbinger* and *ISL2EU* transposon in fish genomes.

### 3.2. Distribution of Harbinger-like Transposons in Medaka Genome

We looked at the distribution of *Harbinger* and *ISL2EU* transposons on medaka chromosomes (Figure S1). We observed that they were homogeneously distributed all along the chromosomes. This suggests that *Harbinger-like* transposons do not have any preferential insertion/retention regions at the genomic scale in the medaka genome.

### 3.3. Evolution of Harbinger Transposons in Teleost Fish Genomes

**Figure 2:**
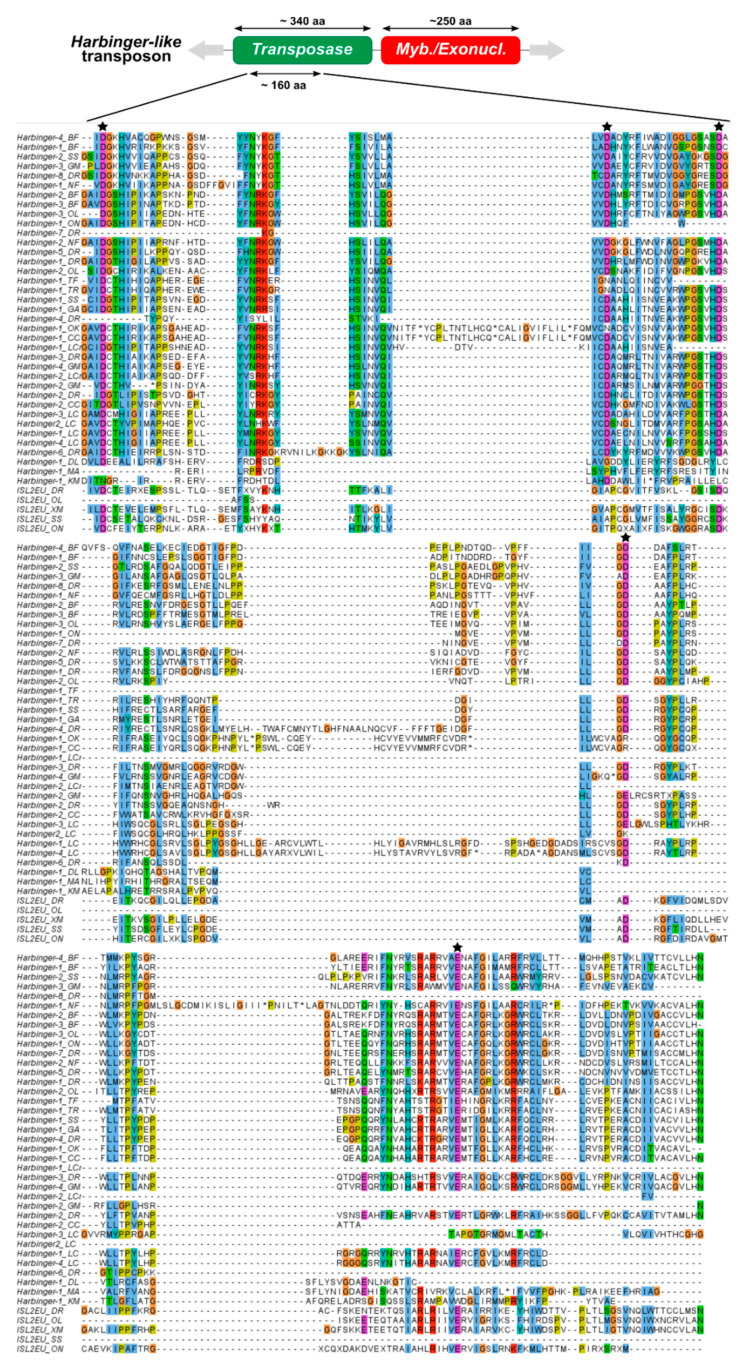
Multiple alignments of the *Harbinger*-like transposase proteins on the DDE domain. Conserved similar amino-acid residues are shown by a same color; black pentagrams indicate the putative catalytic DDE residues. BF: *Branchiostoma floridae* amphioxus, CC: *Cyprinus carpio* carp, DL: *Dicentrarchus labrax* bass, DR: *Danio rerio* zebrafish, GA: *Gasterosteus aculeatus* stickleback, GM: *Gadus morhua* cod, KM: *Kryptolebias marmoratus* killifish, LC: *Latimeria chalumnae* coelacanth, LCr: *Larimichthys crocea* croaker, MA: *Monopterus albus* eel, NF: *Nothobranchius furzeri* killifish, OK: *Oncorhynchus kisutch* silver salmon, OL: *Oryzias latipes* medaka, ON: *Oreochromis niloticus* tilapia, SS: *Salmo salar* salmon, TF: *Takifugu flavidus* fugu, TR: *Takifugu rubripes* fugu, XM: *Xiphophorus maculatus* platyfish.

**Figure 3:**
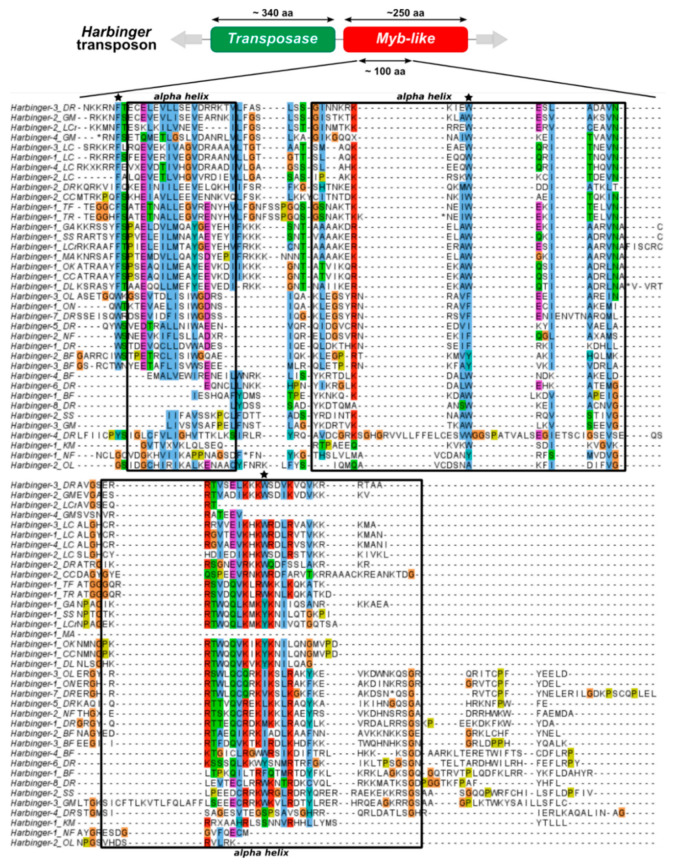
Multiple alignments of the *Harbinger*-like *Myb*-like proteins on the *Myb*-like domain. Predicted alpha-helix motifs are represented with black dashed squares; conserved similar amino-acid residues are shown by a same color; bulky aromatic residues, which are essential for alpha helix structure stabilization, are indicated by black pentagrams. BF: *Branchiostoma floridae* amphioxus, CC: *Cyprinus carpio* carp, DL: *Dicentrarchus labrax* bass, DR: *Danio rerio* zebrafish, GA: *Gasterosteus aculeatus* stickleback, GM: *Gadus morhua* cod, KM: *Kryptolebias marmoratus* killifish, LC: *Latimeria chalumnae* coelacanth, LCr: *Larimichthys crocea* croaker, MA: *Monopterus albus* eel, NF: *Nothobranchius furzeri* killifish, OK: *Oncorhynchus kisutch* silver salmon, OL: *Oryzias latipes* medaka, ON: *Oreochromis niloticus* tilapia, SS: *Salmo salar* salmon, TF: *Takifugu flavidus* fugu, TR: *Takifugu rubripes* fugu, XM: *Xiphophorus maculatus* platyfish.

To investigate the evolution of *Harbinger*-like transposons in fish genomes, representative sequences of 42 *Harbinger* and five *ISL2EU* transposons were further analyzed. These were consensus sequences of different families of *Harbinger*-like transposons from species presented in Figure 1, to which Repbase *Harbinger* sequences of silver salmon, carp, bass, croaker, eel and killifish were added. Multiple alignments of sequences of transposases (for *Harbinger* and *ISL2EU*) and *Myb*-like proteins (for *Harbinger*) were constructed based on the DDE motif (about 160 amino-acids, aa) and the *Myb*-like domain (ca. 100 aa), respectively (Figure 2 and Figure 3). The results indicate conservation of the protein sequences among teleost fish, particularly for the transposase. As expected from the literature, *Myb*-like proteins appear to have been less constrained, but their secondary structure was well conserved [16].

**Figure 4:**
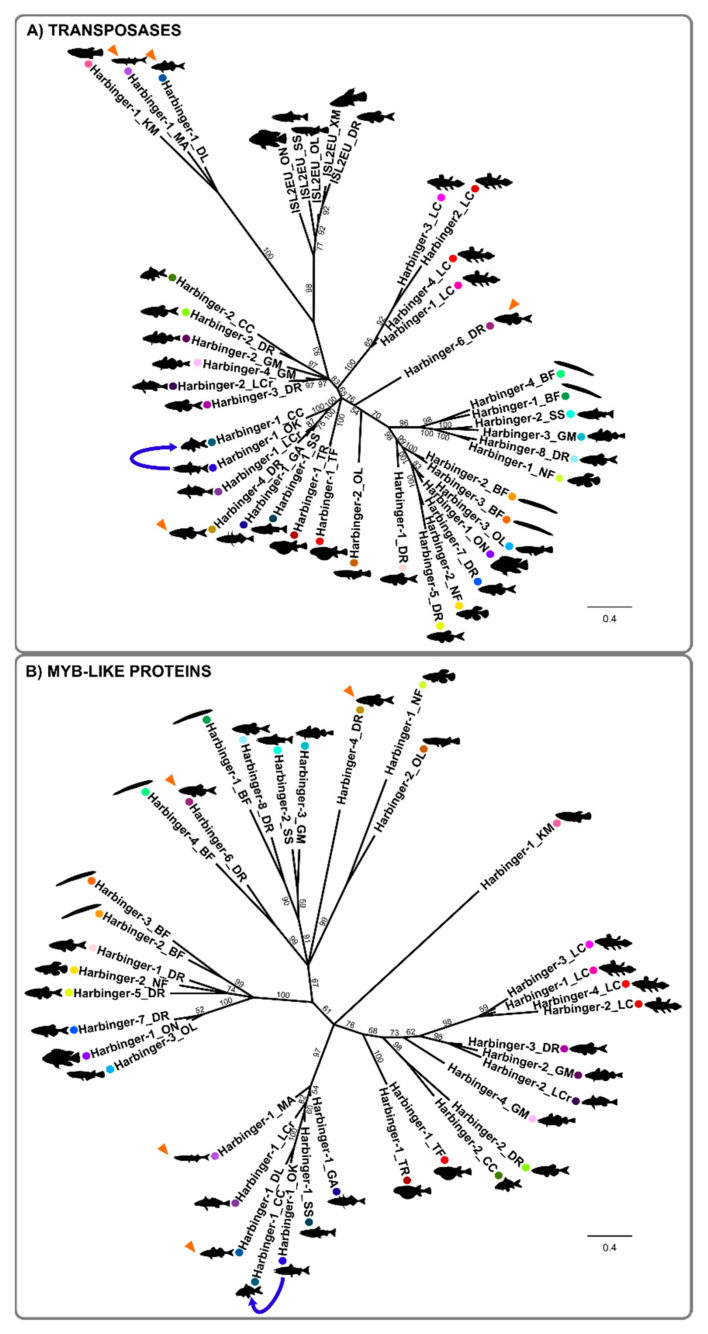
Phylogenetic relationships between *Harbinger* and *ISL2EU* transposases (**A**) and *Myb*-like proteins (**B**) from different fish species. The tree was constructed using the Bayesian method; posterior probability values are indicated [35]. Colored dots indicate the correspondence of the transposases and *Myb*-like proteins from a same *Harbinger* element. The orange arrowheads indicate non-congruent elements between the (**A**,**B**) phylogenies (see Figure 5). The blue arrow indicates a probable case of horizontal transfer. BF: *Branchiostoma floridae* amphioxus, CC: *Cyprinus carpio* carp, DL: *Dicentrarchus labrax* bass, DR: *Danio rerio* zebrafish, GA: *Gasterosteus aculeatus* stickleback, GM: *Gadus morhua* cod, KM: *Kryptolebias marmoratus* killifish, LC: *Latimeria chalumnae* coelacanth, LCr: *Larimichthys crocea* croaker, MA: *Monopterus albus* eel, NF: *Nothobranchius furzeri* killifish, OK: *Oncorhynchus kisutch* silver salmon, OL: *Oryzias latipes* medaka, ON: *Oreochromis niloticus* tilapia, SS: *Salmo salar* salmon, TF: *Takifugu flavidus* fugu, TR: *Takifugu rubripes* fugu, XM: *Xiphophorus maculatus* platyfish.

In order to study *Harbinger*-like transposon evolution, phylogenetic trees were constructed based on transposase DDE domain multiple alignment using the Bayesian (Figure 4A) and maximum likelihood methods (Figure S2A) [33,35]. The results confirmed that *ISL2EU* transposons form a phylogenetic group distinct from *Harbinger* elements. We observed that many *Harbinger* sequences were more related to elements from other species than to sequences from the same species, thereby defining different *Harbinger* families. Some teleost *Harbinger* elements grouped with amphioxus sequences, indicating families possibly present in a common chordate ancestor, or alternatively, horizontal transfer, a hypothesis that should be addressed by comparing the divergence of TE copies and non-TE sequences from the two lineages. Some other teleost *Harbinger* sequences were related neither to coelacanth nor to amphioxus families, suggesting teleost-specific family expansion and divergence, or horizontal transfer from more divergent organisms. Finally, coelacanth *Harbinger* transposon sequences preferentially grouped together and formed a group distinct from other fish transposons, indicating specific family expansion in the coelacanth genome or in one of its sarcopterygian ancestors.

Including in the analysis transposase sequences from 154 manually-annotated individual *Harbinger* copies from different fish species did not significantly modify the topology of the tree, indicating that consensus sequences were likely to be representative of the diversity of these transposons in fish (Figure S3). However, copies from different fish species grouped together with *Harbinger-2_OL* and *Harbinger-6_DR*, which stood alone in the consensus phylogeny, hence uncovering two additional families of fish *Harbinger* elements (Figure 4 and Figure S3).

Within both transposase and *Myb*-like molecular phylogenies, some sequences from divergent fish species appeared to be more related than expected from species relationships. This was particularly the case for *Harbinger-1_OK* from the salmonid *Oncorhynchus kisutch* (silver salmon) and *Harbinger-1_CC* from the cyprinid *Cyprinus carpio* (European carp), which were very close in the phylogenies (Figure 4 and Figure S2). *Harbinger-1_OK*-related sequences were detected in other salmonids, including the Atlantic salmon *Salmo salar,* and in the Northern pike *Esox lucius* from the related family Esocidae (ca. 80 million years divergence) (data not shown). In contrast, besides *C. carpio*, no sequence closely related to *Harbinger-1_OK* was found in the ten other cyprinid fish genus that were tested (data not shown). Strikingly, the level of nucleotide identity between elements from salmonids and *C. carpio* (96–98%), which diverged ca. 220 million years ago, was similar to that observed between the salmonid species tested (97–99%, maximal divergence 30 million years ago) and much higher than between salmonids and the pike *E. lucius* (71–73%, divergence ca. 80 million years ago). Hence, these results strongly support horizontal transfer between a salmonid fish and an ancestor of *C. carpio* after its divergence from most other cyprinid species. Such an event of horizontal transfer has already been suggested by Zhang et al. (2020) in their vertebrate-wide large survey of the horizontal transfer of transposable elements [42].

### 3.4. The Evolution of the Myb-like Proteins Recapitulates the Evolution of Transposase Proteins of Harbinger Transposons

The presence of two independent ORFs in *Harbinger* elements is an unusual feature in DNA transposons. Until this study, the evolutionary history of *Harbinger* transposons was only studied through large-scale analyses of their transposases [16,22,23]. Moreover, while transposases have a high degree of conservation even between different families of *Harbinger* transposons, the *Myb*-like proteins are much more diverse and display only some similarity in restricted parts of the *Myb*-like domain (Figure 2 and Figure 3) [16]. Thus, the evolution of this second ORF has been poorly studied so far [16].

**Figure 5:**
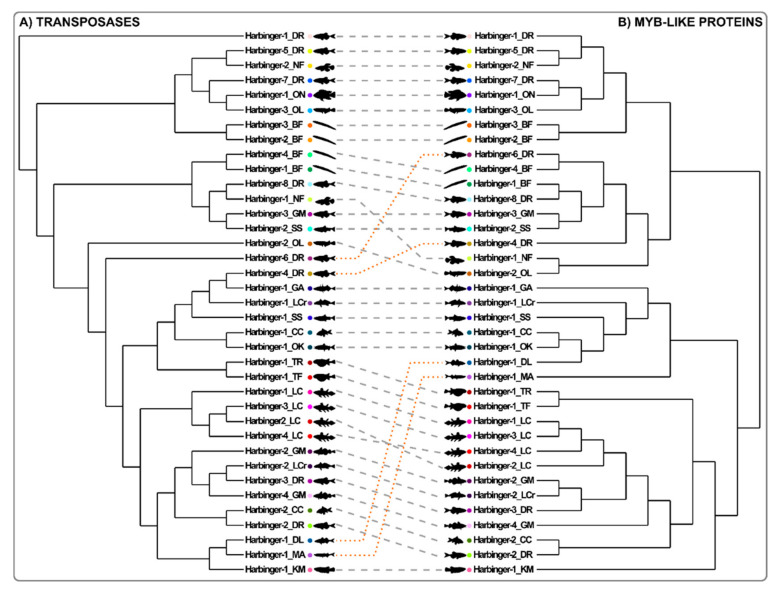
Phylogenetic congruence between transposases and *Myb*-like proteins of *Harbinger* transposons from fish genomes. The trees of the transposases (**A**, cf. Figure 4A) and *Myb*-like (**B**, cf. Figure 4B) proteins were constructed using the Bayesian method [35]. The correspondence of the transposases and *Myb*-like proteins from a same *Harbinger* element are indicated with colored dots and connected by dashed lines. Non-congruent elements are indicated with orange dashed lines. See the legend of Figure 4 for species name abbreviations.

The *Myb*-like proteins associated with the transposases of the *Harbinger* transposons presented in Figure 4A (and Figure S2A) were aligned on their *Myb*-like domains (ca. 85 aa), and a phylogenetic tree was constructed using both Bayesian and maximum likelihood methods (Figure 4B and Figure S2B) [33,35]. We calculated the congruence index Icong [43] between the phylogenies of *Harbinger* transposases and *Myb*-like proteins, which revealed that the trees are more congruent than expected by chance (*p*-value = 0.005) (Figure 5). The results therefore suggested a unique origin of the transposase and *Myb*-like gene association in *Harbinger* transposons. However, we noticed that the phylogenetic positions of four sequences (indicated with orange arrowheads in Figure 4) were different in the transposase and *Myb*-like Bayesian phylogenies. The phylogenetic positions of the proteins of these elements *(Harbinger-1_DL*, *Harbinger-1_MA*, *Harbinger-4_DR*, *Harbinger-6_DR*) were well supported (Figure 4), suggesting potential recombination events between the transposase and the *Myb*-like genes in these *Harbinger* sequences.

### 3.5. Harbinger-like Transposons Are Expressed in Fish

Despite the broad distribution of *Harbinger* transposons, studies on their activity remain scarce [27,28,29]. To the best of our knowledge, no proof of activity has been reported so far for *ISL2EU* transposons.

**Figure 6:**
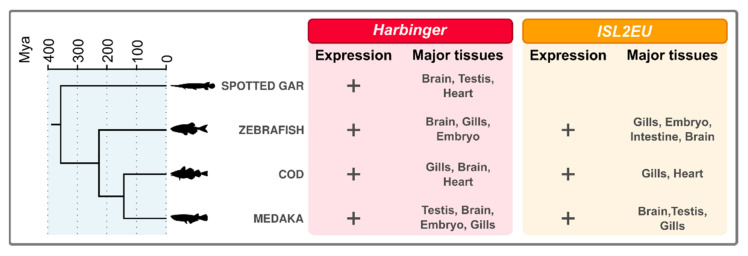
Expression analysis of *Harbinger* and *ISL2EU* transposons in spotted gar, zebrafish, cod and medaka using the PhyloFish database [37]. Expression identified in PhyloFish database is indicated with +. Absence of “+” indicates that the element was not detected in the genome. For each species, the organs where the TEs are mainly expressed are indicated.

Using the PhyloFish database, which allows investigating sequence expression thanks to multiple fish transcriptome datasets from multiple organs, *Harbinger*-like transposon expression was detected in numerous fish species. Particularly, expression datasets were accessible for four of the species studied in Figure 1: spotted gar, zebrafish, cod and medaka. Expression of *Harbinger* and *ISL2EU* transposons was found in all four species (except for *ISL2EU* transposons, which are absent from spotted gar) (Figure 6). *Harbinger* and *ISL2EU* were particularly highly expressed in organs such as brain and gills, and in testis and embryos in some species.

**Figure 7:**
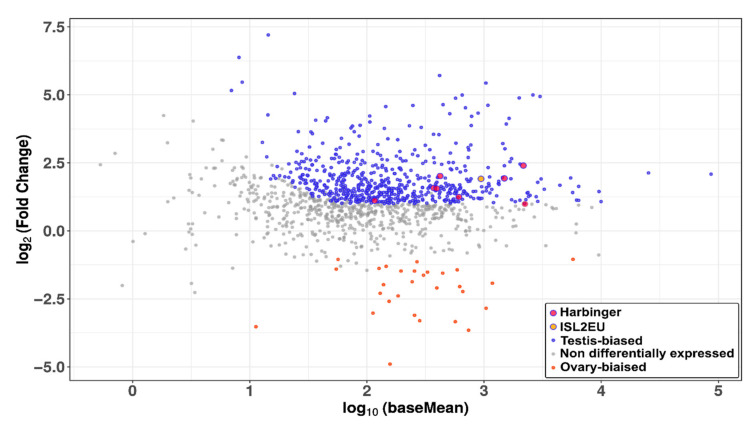
MAplot representing the relative expression in male and female gonads of all TE families of the medaka genome. Each dot corresponds to the relative expression of one TE family in RNAseq data. The x-axis corresponds to the signal intensity averaged across all replicates, and the y-axis to the log2 fold change of expression between testis and ovary (log2FC). The higher the log2FC of a TE family, the more it is over-expressed in testes (significant over-expression in testes is indicated in purple), and the lower it is, the more it is over-expressed in ovaries (significant over-expression in ovaries is indicated in orange). The further the gene is on the right, the more it is expressed overall, across all replicates. The *Harbinger* and *ISL2EU* families are highlighted with bigger red- or yellow-colored dots, respectively.

We further investigated expression in the medaka, the species with the highest genome coverage of *Harbinger* transposons in our analysis (Figure 1). We focused on medaka gonads, since transposition activity in germ cells allows transmission of new insertions and expansion of TE families. Analysis of RNAseq data showed expression of both *Harbinger* and *ISL2EU* transposons in both male and female gonads, each family showing similar expression levels (Figure 7). Transposon expression was testis-biased, i.e., higher in male than in female gonads.

## 4. Discussion

### 4.1. Harbinger-like Transposons Are Inequitably Widespread in Fish Genomes

*Harbinger*-like transposons are found in diverse eukaryotic clades, such as vertebrates, arthropods, fungi and plants [13,14,16,17,18,20,21,22,23]. Within vertebrates, they are absent from mammalian genomes but have been identified in other vertebrate species, including crocodilians, turtles and fish (https://www.girinst.org/repbase/, accessed on 15 September 2021) [16,22,44]. Teleost fish, which are the most species-rich clade within vertebrates, have genomes with highly diversified TE composition, both quantitatively and qualitatively, particularly compared to mammals and birds. Therefore, focusing on this group of animals is of particular relevance to the evolutionary history of *Harbinger*-like transposons. Here, we have focused our analysis on eight teleost fish species, one non-teleost ray-finned fish (spotted gar), one sarcopterygian fish (coelacanth), one cartilaginous fish (elephant shark) and one cephalochordate (amphioxus) (Figure 1). This led us to the observation that *Harbinger* transposons are widespread in ray-finned fish, including teleosts, but in variable amounts; the highest genomic success was for the medaka. They are also present at high copy numbers in coelacanth and amphioxus but are absent from the elephant shark genome, suggesting secondary loss of these elements during the evolution of cartilaginous fish [16,22]. However, other species of cartilaginous fish must be analyzed to test for the presence/absence of *Harbinger* transposons in this lineage. A putative case of horizontal transfer of a *Harbinger* element from a salmonid into a cyprinid fish was detected, but such events might be relatively rare for *Harbinger* transposons in vertebrates [42].

*ISL2EU* elements are scarcer. They were not detected in the non-teleost species included in this study, i.e., amphioxus, elephant shark, spotted gar and coelacanth. Within teleost fish, *ISL2EU* transposons were found in zebrafish, cod, platyfish, medaka and tilapia; but neither were in stickleback, nor in the two pufferfishes, fugu and tetraodon. We propose that *ISL2EU* elements might have been introduced ca. 200–300 million years ago at the base of the teleost fish lineage through horizontal transfer from a more divergent species; and that there was subsequent secondary loss in a common ancestor of stickleback and pufferfishes. Overall, our results suggest that, even if *Harbinger* and *ISL2EU* transposons are related, they present different evolutionary dynamics that might be due to different abilities to invade genomes or different mechanisms of repression in their hosts.

### 4.2. Evolutionary Relationships between the Two ORFs of Harbinger Transposons

In order to investigate the evolutionary dynamics of *Harbinger*-like transposons, we studied the phylogenetic relationships of the transposases of elements from different fish genomes, focusing on the DDE domain (Figure 4A). This allowed the identification of several families of fish *Harbinger* transposons. Some of them might be more ancient, dating back to a chordate common ancestor of amphioxus and fish. In contrast, others are apparently more recent and teleost-specific.

Furthermore, we studied the phylogenetic relationships between the *Myb*-like proteins of *Harbinger* transposons from different species. *Myb*-like proteins were less constrained than transposases, although their secondary structure was well conserved (Figure 2 and Figure 3). Transposases were highly similar even between different families of *Harbinger* transposons. In contrast, *Myb*-like proteins were diverse and presented only sporadic similarities in restricted parts of the *Myb*-like domain, making their comparison more difficult [16]. This raises the question of whether the two *Harbinger* proteins followed the same evolutionary trajectories. In teleost fish genomes, transposase and *Myb*-like protein phylogenies were consistent with respect to the element they belonged together (Figure 4 and Figure S2). This indicated that, even if the two types of proteins presented different degrees of conservation, they shared common evolutionary path within *Harbinger* transposons. Hence, our results were consistent with a single origin of the association between transposase and *Myb*-like protein in *Harbinger*, though there were possible rare events of recombination during evolution between elements belonging to different families. Such a recombination might be restricted by the fact that co-evolution between the two ORFs within a same element is necessary to maintain interactions between the two proteins for a successful transposition.

Some peculiar *Harbinger*-like transposons called *Spy*, characterized by a single ORF, have been identified in invertebrates [22,25]. This ORF encodes a transposase with a DDE domain but also a helix–turn–helix (HTH) motif, which is believed to act as a sequence-specific DNA-binding domain. Hence, this HTH motif might fulfil the same function as the DNA-binding domain of the *Myb*-like protein in *Harbinger* elements. We did not identify any *Harbinger* transposon with a single ORF in teleost fish, confirming that both ORFs are probably essential for autonomous *Harbinger* transposition in this clade. To date, single ORF *Harbinger* transposons have only been found outside of vertebrates [22,25]. This could suggest either that the structure with two ORFs of the *Harbinger* transposons is more efficient for their spreading and maintenance in fish, or that the single ORF type has been more easily repressed and eliminated in the lineage that led to vertebrates.

### 4.3. Harbinger-Like Transposons Are Transcriptionally Active in Teleost Fish

We report here the expression of *Harbinger*-like elements in teleost fish. Using the PhyloFish database [37], we detected the expression of these transposons in spotted gar, zebrafish, cod and medaka (Figure 6). *Harbinger* and *ISL2EU* transposons were mainly expressed in the same tissues in these species, suggesting common mechanisms of activation and repression. Moreover, in the medaka, both *Harbinger* and *ISL2EU* transposons showed testis-biased expression in gonads (Figure 7), as reported for other transposable elements in other organisms [45,46].

TEs are repressed in genomes, limiting their potential deleterious effects through insertional mutagenesis. This repression can occur through the prevention of transcription (generally with epigenetic marks, such as DNA methylation or histone modifications) or post-transcriptionally (with piRNAs, for example) [47,48,49,50]. In the medaka, piRNAs, which can mediate the cleavage of transposable element mRNAs, are more expressed in testis compared to ovaries [51]. However, TE expression is the result of both transcription and repression. Therefore, the higher expression we observed in the testis for *Harbinger*-like transposons is probably due to stronger transcription in this organ, which would not be completely compensated for by piRNA inhibition, if at all.

## 5. Conclusions

This work characterized *Harbinger*-like transposons in teleost fish genomes. Even if these elements represent small parts of these genomes, they are widespread in this clade. *Harbinger* and *ISL2EU* transposons are also transcriptionally active in fish. Since their discovery, beyond their neutral or negative effects, multiple works have demonstrated the propensity of TEs to be positively recruited by host genomes as new regulatory and coding sequences [5,52,53]. *Harbinger* transposons are not exceptions to this, as multiple cases of *Harbinger*-derived genes have been reported in various organisms [16,18,24,54,55,56,57,58]. The ability of *Harbinger* transposons to form new genes may be linked to the presence of two ORFs encoding proteins with useful and different molecular properties that can interact together [24].

A recent study has shown that *Harbinger* transposons invaded the genome of sea kraits, *Laticauda*, about 15–25 million years ago, and compose as much as 8–12% of their DNA [44]. In these organisms, several insertions occurred in introns, regulatory regions and exons; and even coding sequences have been added into exons, conferring potential adaptation. In the tomato *Solanum lycopersicum*, light stress conditions induce expression of genes having *Harbinger* transposons (among other TEs) located in their genomic proximity (mostly in their introns) [59]. The authors suggest that these elements serve in a stress regulatory network to adapt rapidly to new environments. Thus, *Harbinger* transposons represent an interesting and beneficial reservoir of useful sequences for species adaptation in teleost fish and other organisms.

## Data Availability

Data availability is indicated in Section 2.

## Supplementary Materials

The following supporting information can be downloaded at: https://www.mdpi.com/article/10.3390/ani12111429/s1, Supplementary Data S1: Predicted fish *Harbinger* transposase proteins; Supplementary Data S2: Predicted fish *Harbinger Myb*-like proteins; Supplementary Data S3: Genome coverage script; Figure S1: Distribution of Harbinger and ISL2EU transposons on medaka chromosomes; Figure S2: Phylogenetic relationships between Harbinger transposases and Myb-like proteins of different fish species; Figure S3: Phylogenetic relationships between *Harbinger* and *ISL2EU* transposase consensus and individual copy protein sequences from different fish species.

## References

[1] Wicker T., Sabot F., Hua-Van A., Bennetzen J.L., Capy P., Chalhoub B., Flavell A., Leroy P., Morgante M., Panaud O. (2007). A Unified Classification System for Eukaryotic Transposable Elements. Nat. Rev. Genet..

[2] Kidwell M.G., Lisch D.R. (2000). Transposable Elements and Host Genome Evolution. Trends Ecol. Evol..

[3] Volff J.-N. (2005). Genome evolution and biodiversity in teleost fish. Heredity.

[4] Chuong E.B., Elde N.C., Feschotte C. (2016). Regulatory evolution of innate immunity through co-option of endogenous retroviruses. Science.

[5] Chuong E., Elde N.C., Feschotte C. (2016). Regulatory activities of transposable elements: From conflicts to benefits. Nat. Rev. Genet..

[6] Jangam D., Feschotte C., Betrán E. (2017). Transposable Element Domestication as an Adaptation to Evolutionary Conflicts. Trends Genet..

[7] Van Der Laan R., Eschmeyer W.N., Fricke R. (2014). Family-group names of Recent fishes. Zootaxa.

[8] Fricke R., Eschmeyer W.N., Van der Laan R. (2022). Eschmeyer’s Catalog of Fishes: Genera, Species, References.

[9] Chalopin D., Naville M., Plard F., Galiana D., Volff J.-N. (2015). Comparative Analysis of Transposable Elements Highlights Mobilome Diversity and Evolution in Vertebrates. Genome Biol. Evol..

[10] Carducci F., Barucca M., Canapa A., Carotti E., Biscotti M.A. (2020). Mobile Elements in Ray-Finned Fish Genomes. Life.

[11] Kapitonov V.V., Jurka J. (1999). Molecular Paleontology of Transposable Elements from Arabidopsis Thaliana. Genetica.

[12] Jurka J., Kapitonov V.V. (2001). PIFs Meet Tourists and *Harbingers*: A Superfamily Reunion. Proc. Natl. Acad. Sci. USA.

[13] Zhang X., Feschotte C., Zhang Q., Jiang N., Eggleston W.B., Wessler S.R. (2001). P Instability Factor: An Active Maize Transposon System Associated with the Amplification of Tourist-like MITEs and a New Superfamily of Transposases. Proc. Natl. Acad. Sci. USA.

[14] Jiang N., Bao Z., Zhang X., Hirochika H., Eddy S., McCouch S.R., Wessler S.R. (2003). An active DNA transposon family in rice. Nature.

[15] Kikuchi K., Terauchi K., Wada M., Hirano H.-Y. (2003). The plant MITE mPing is mobilized in anther culture. Nature.

[16] Kapitonov V.V., Jurka J. (2004). *Harbinger* Transposons and an Ancient HARBI1 Gene Derived from a Transposase. DNA Cell Biol..

[17] Zhang X., Jiang N., Feschotte C., Wessler S.R. (2004). *PIF*- and *Pong*-Like Transposable Elements: Distribution, Evolution and Relationship with *Tourist*-Like Miniature Inverted-Repeat Transposable Elements. Genetics.

[18] Casola C., Lawing A.M., Betrán E., Feschotte C. (2007). PIF-like Transposons are Common in Drosophila and Have Been Repeatedly Domesticated to Generate New Host Genes. Mol. Biol. Evol..

[19] Grzebelus D., Lasota S., Gambin T., Kucherov G., Gambin A. (2007). Diversity and structure of PIF/*Harbinger*-like elements in the genome of Medicago truncatula. BMC Genom..

[20] Yuan Y.-W., Wessler S.R. (2011). The catalytic domain of all eukaryotic cut-and-paste transposase superfamilies. Proc. Natl. Acad. Sci. USA.

[21] Pereira J.F., Almeida A.P.M.M., Cota J., Pamphile J.A., da Silva G.F., de Araújo E.F., Gramacho K.P., Brommonschenkel S.H., Pereira G.A.G., de Queiroz M.V. (2013). Boto, a class II transposon in Moniliophthora perniciosa, is the first representative of the PIF/*Harbinger* superfamily in a phytopathogenic fungus. Microbiology.

[22] Han M.-J., Xiong C.-L., Zhang H.-B., Zhang M.-Q., Zhang H.-H., Zhang Z. (2015). The diversification of PHIS transposon superfamily in eukaryotes. Mob. DNA.

[23] Markova D.N., Mason-Gamer R.J. (2015). Diversity, abundance, and evolutionary dynamics of Pong-like transposable elements in Triticeae. Mol. Phylogenetics Evol..

[24] Sinzelle L., Kapitonov V.V., Grzela D.P., Jursch T., Jurka J., Izsvák Z., Ivics Z. (2008). Transposition of a Reconstructed *Harbinger* Element in Human Cells and Functional Homology with Two Transposon-Derived Cellular Genes. Proc. Natl. Acad. Sci. USA.

[25] Han M.-J., Xu H.-E., Zhang H.-H., Feschotte C., Zhang Z. (2014). Spy: A New Group of Eukaryotic DNA Transposons without Target Site Duplications. Genome Biol. Evol..

[26] Hancock C.N., Zhang F., Wessler S.R. (2010). Transposition of the Tourist-MITE mPing in yeast: An assay that retains key features of catalysis by the class 2 PIF/*Harbinger* superfamily. Mob. DNA.

[27] Elewa A., Wang H., Talavera-López C., Joven A., Brito G., Kumar A., Hameed L.S., Penrad-Mobayed M., Yao Z., Zamani N. (2017). Reading and editing the Pleurodeles waltl genome reveals novel features of tetrapod regeneration. Nat. Commun..

[28] Markova D.N., Mason-Gamer R.J. (2017). Transcriptional Activity of PIF and Pong-like Class II Transposable Elements in Triticeae. BMC Evol. Biol..

[29] Smith J.J., Sumiyama K., Amemiya C.T. (2012). A Living Fossil in the Genome of a Living Fossil: *Harbinger* Transposons in the Coelacanth Genome. Mol. Biol. Evol..

[30] Kapitonov V.V., Jurka J. (2008). A Universal Classification of Eukaryotic Transposable Elements Implemented in Repbase. Nat. Rev. Genet..

[31] Amemiya C.T., Alföldi J., Lee A.P., Fan S., Philippe H., Maccallum I., Braasch I., Manousaki T., Schneider I., Rohner N. (2013). The African Coelacanth Genome Provides Insights into Tetrapod Evolution. Nature.

[32] Katoh K., Misawa K., Kuma K., Miyata T. (2002). MAFFT: A Novel Method for Rapid Multiple Sequence Alignment Based on Fast Fourier Transform. Nucleic Acids Res..

[33] Guindon S., Gascuel O. (2003). A Simple, Fast, and Accurate Algorithm to Estimate Large Phylogenies by Maximum Likelihood. Syst. Biol..

[34] Le S.Q., Gascuel O. (2008). An Improved General Amino Acid Replacement Matrix. Mol. Biol. Evol..

[35] Huelsenbeck J.P., Ronquist F. (2001). MRBAYES: Bayesian Inference of Phylogenetic Trees. Bioinformatics.

[36] Darriba D., Taboada G.L., Doallo R., Posada D. (2011). ProtTest 3: Fast Selection of Best-Fit Models of Protein Evolution. Bioinformatics.

[37] Pasquier J., Cabau C., Nguyen T., Jouanno E., Severac D., Braasch I., Journot L., Pontarotti P., Klopp C., Postlethwait J.H. (2016). Gene Evolution and Gene Expression after Whole Genome Duplication in Fish: The PhyloFish Database. BMC Genom..

[38] Mariette J., Noirot C., Nabihoudine I., Bardou P., Hoede C., Djari A., Cabau C., Klopp C. (2014). RNAbrowse: RNA-Seq de Novo Assembly Results Browser. PLoS ONE.

[39] Dechaud C., Miyake S., Martinez-Bengochea A., Schartl M., Volff J.-N., Naville M. (2021). Clustering of Sex-Biased Genes and Transposable Elements in the Genome of the Medaka Fish Oryzias Latipes. Genome Biol. Evol..

[40] Yang W.R., Ardeljan D., Pacyna C.N., Payer L.M., Burns K.H. (2019). SQuIRE Reveals Locus-Specific Regulation of Interspersed Repeat Expression. Nucleic Acids Res..

[41] Kumar S., Stecher G., Suleski M., Hedges S.B. (2017). TimeTree: A Resource for Timelines, Timetrees, and Divergence Times. Mol. Biol. Evol..

[42] Zhang H.-H., Peccoud J., Xu M.-R.-X., Zhang X.-G., Gilbert C. (2020). Horizontal Transfer and Evolution of Transposable Elements in Vertebrates. Nat. Commun..

[43] De Vienne D.M., Giraud T., Martin O.C. (2007). A Congruence Index for Testing Topological Similarity between Trees. Bioinformatics.

[44] Galbraith J.D., Ludington A.J., Sanders K.L., Suh A., Adelson D.L. (2021). Horizontal Transfer and Subsequent Explosive Expansion of a DNA Transposon in Sea Kraits (Laticauda). Biol. Lett..

[45] Yin W., Wang Z.-J., Li Q.-Y., Lian J.-M., Zhou Y., Lu B.-Z., Jin L.-J., Qiu P.-X., Zhang P., Zhu W.-B. (2016). Evolutionary Trajectories of Snake Genes and Genomes Revealed by Comparative Analyses of Five-Pacer Viper. Nat. Commun..

[46] Stow E.C., Kaul T., deHaro D.L., Dem M.R., Beletsky A.G., Morales M.E., Du Q., LaRosa A.J., Yang H., Smither E. (2021). Organ-, Sex- and Age-Dependent Patterns of Endogenous L1 MRNA Expression at a Single Locus Resolution. Nucleic Acids Res..

[47] Zempleni J., Chew Y.C., Bao B., Pestinger V., Wijeratne S.S.K. (2009). Repression of Transposable Elements by Histone Biotinylation. J. Nutr..

[48] Rebollo R., Romanish M.T., Mager D.L. (2012). Transposable Elements: An Abundant and Natural Source of Regulatory Sequences for Host Genes. Annu. Rev. Genet..

[49] Iwasaki Y.W., Siomi M.C., Siomi H. (2015). PIWI-Interacting RNA: Its Biogenesis and Functions. Annu. Rev. Biochem..

[50] Sarkar A., Volff J.-N., Vaury C. (2017). PiRNAs and Their Diverse Roles: A Transposable Element-Driven Tactic for Gene Regulation?. FASEB J..

[51] Kneitz S., Mishra R.R., Chalopin D., Postlethwait J., Warren W.C., Walter R.B., Schartl M. (2016). Germ Cell and Tumor Associated PiRNAs in the Medaka and Xiphophorus Melanoma Models. BMC Genom..

[52] Etchegaray E., Naville M., Volff J.-N., Haftek-Terreau Z. (2021). Transposable Element-Derived Sequences in Vertebrate Development. Mob DNA.

[53] Cosby R.L., Chang N.-C., Feschotte C. (2019). Host-Transposon Interactions: Conflict, Cooperation, and Cooption. Genes Dev.

[54] Liang S.C., Hartwig B., Perera P., Mora-García S., de Leau E., Thornton H., de Lima Alves F., de Alves F.L., Rappsilber J., Rapsilber J. (2015). Kicking against the PRCs—A Domesticated Transposase Antagonises Silencing Mediated by Polycomb Group Proteins and Is an Accessory Component of Polycomb Repressive Complex 2. PLoS Genet..

[55] Duan C.-G., Wang X., Xie S., Pan L., Miki D., Tang K., Hsu C.-C., Lei M., Zhong Y., Hou Y.-J. (2017). A Pair of Transposon-Derived Proteins Function in a Histone Acetyltransferase Complex for Active DNA Demethylation. Cell Res..

[56] Velanis C.N., Perera P., Thomson B., de Leau E., Liang S.C., Hartwig B., Förderer A., Thornton H., Arede P., Chen J. (2020). The Domesticated Transposase ALP2 Mediates Formation of a Novel Polycomb Protein Complex by Direct Interaction with MSI1, a Core Subunit of Polycomb Repressive Complex 2 (PRC2). PLoS Genet..

[57] Cosby R.L., Judd J., Zhang R., Zhong A., Garry N., Pritham E.J., Feschotte C. (2021). Recurrent Evolution of Vertebrate Transcription Factors by Transposase Capture. Science.

[58] Zhou X., He J., Velanis C.N., Zhu Y., He Y., Tang K., Zhu M., Graser L., de Leau E., Wang X. (2021). A Domesticated *Harbinger* Transposase Forms a Complex with HDA6 and Promotes Histone H3 Deacetylation at Genes but Not TEs in Arabidopsis. J. Integr. Plant Biol..

[59] Deneweth J., Van de Peer Y., Vermeirssen V. (2022). Nearby Transposable Elements Impact Plant Stress Gene Regulatory Networks: A Meta-Analysis in A. Thaliana and S. Lycopersicum. BMC Genom..

