# Supplementary material for: Diversity of Harbinger-like Transposons in Teleost Fish Genomes
Source: Animals (Basel). 2022 May 31;12(11):1429. doi: 10.3390/ani12111429 (PMC9179366; doi:10.3390/ani12111429)
Supplement: Supplementary file 1 [file animals-12-01429-s001.zip › animals-1703301-supplementary.pdf]

animals

MDPI

Supplementary materials

Supplementary data S1. Predicted fish *Harbinger* transposase proteins.

```
>Harbinger-1_OK
FLSALTMACPFVRDVVDeealvlrrafrrervfrdrlDPLAFPDDHLYERYRFSADGIRY
LCRLLGPRIKHRTARSHALSVEQMVCVALRFFASGAFLYSVGDAEQLNKATICRTIRSVC
LAIKALADVFISFPGHRRLCDIKEEFYRIAGKRIYKLQDNC*HIVGYSLLCVTGFPNVIG
AVDCTHIRIKAPSGAHEADFVNRKSFHSINVQVCNADCVISNVVAKWPGSVHDSRIFRAS
EIYQCLSQGKPHNPYL*PSWLCQEYHCVYEVVMMRFCVDR*ILWCVAGRQGYGCQPFLLT
PFTDPQEAQQAYNHAHARTRARVEMTFGLLKARFHCLHKLRVSPVRACD
ITVACAVLHNVAC
>Harbinger-1_CC
QMVCVALRFFASGAFLYSVGDAEQLNKATICRTIRSVCLAIKALADVFISFPGHRRLCDM
KEEFYRIAGKRIYKLQDNC*HIVGYSLLCVTGFPNVIGAVDCTHIRIKAPSGAHEADFVN
RKSFHSINVQVNIVCDADCVISNVVAKWPGSVHDSRIFRASEIYQRLSQGKPHNPYL*PS
WLCQEYHCVYEVVMMRFCVDR*ILWCVAGRQGYGCQXFLLTPFTDPQEAQQAYNHAHART
RARVEMTFGLLKARFHCLRELRVNPVRACDITVACAVLHNVAC
>Harbinger-2_CC
NISRVIGITDGTLIPVSNPVVNEPLYIYRKGYPAINVQIVCDHKGMFNDIVAKWLGSTHD
SFVWATSAVCRWLKRVHGFGXSRLLGDSGYPLHPYLLTPVPHPATTA
>Harbinger-1_DL
MACPFLEDVLDEEALILRRAFSHERVFRDRSDPLAVGDDYLIERYRFSGDGLRYLCRLL
GPKIQHQTAGSHALTVPQMVCVTLRCFASGSFLYSVGDAENLNKGTICSTYPGLVGRPL
CQFYGLFLTATIIQTTFNMSA*SIHLICSLLSTLXSXTSLQNVLVFNFNLLPGPFMARQ
VCSL
>Harbinger-1_LCr
QRLNMACPFNHDPVDEGAAILRRELHICRQRVLRPRIDISALPDNFLFERYRFTSQSIIY
IHNLIRPHISNITSRSHALTSQQILCVALRFFANGSFLYNIGDAEHLSKATVCRAIRRVC
LALKRLLPIXVVFPGHKPVRAIKEEFHRIAGEGCRDLLNSFNIRLPNVIGCIDGTHIPIT
APPSHNEADYVNRKSIHSINVQVHVDTVKIICDAAHIISNVEA
>Harbinger-2_LCr
RRERFKERKDLLANHDNWLISRFRLPKASVTGVTAELRPARERNGGRRAGCLTALGFPAT
GAFQRELADRSGVCQLTLSRAMPAVWDGIIRMSSWYIRFPYSAVEQADIKAQFAARAGFP
NVIGAIDCTHIAIKAPSQDDFFYVSRKHFHSINVQIICDARMQLTNIVARWPGSTHDSFI
MTNSIAENRLEAGTVRDGWLLFV
>Harbinger-1_MA
RERILRPRVDFLSYPHVFLFERYRFSRESITYINNLIHPYIRHITHRGRALTSEQMLCVA
LRFVANGSFLYNIGDAEHISKATVCRIVRKVCLALKRFL*IFVVFPGHKPLRAIKEEFHR
IAG*WY*IRFSKANLKNDDQLITSCCDLWK*TNN*NPLQDFLMRLDALMAHIFPSMH
```

>Harbinger-1_KM
MAALALLEDITNGRIRRERVFRDHTDLLAHDDAWLII*FRVPRAILLELCAELAPALHRE
TRRSRALPVPVQVLTTLGFLATGAFQRELADRSGISQSSLSRAMPAVWDGLIRMMPRYIK
FPYTVAETLRRCAATRFLTSTI
>Harbinger-5_DR
FQMLHHYYTNILRTSEVWAYSRTSDWWENTAQKYTDDQWLEDFRVSRETFRYICSTLKPS
LQRLDTSFRLCIPLAKRVAIALYKLASTTEYRTVANLFAVSRTSVCRCVHDFCKAVIAVL
RPKLINTPDQAKMAEIADYFEDKFGIPQCVGAIDGSHIPILKPPQYQSDFHNRKGWHSII
LQAVVDGKGLFWDLNVGQPGREHDASVLKKSCLWTWATSTTAFPGRVKNICGTEVGYFIL
GDSAYPLQKWLLKPYPDTGRLTEAQELYNMRTSRARCVVEHAFGRLKGRWKCLSKRNDCN
VNVVVDMVETCCTLHNLCE
>Harbinger-1_ON
TMALFGAVAATAVQRYIWTRSRKQEWWDWDVRGFTDTEFIHNFRMSRQTFEYLCQLLSAR
MLRQDTHLRQSIPVSKRVGIGLYWLATGACYRTMSNLFGVAKSTVCSIVHEFCFAVRYVL
MPEYIKWPRGDDLIQVIEGFRQRWGFPQCAGAIDGSHIPITAPEDNHCDYFNRKGWHSVI
LQGVVDHQFWMGVEVPVMLLGDPAYPLRSWLLKGYADTGTLTEEQQYFNQRHSRARMTVE
CAFGQLKGRWRCLGKRLDVDIHTVPTIIAACCTLHNVCE
>Harbinger-1_DR
FAALLSDYSSCTSIRTTWIRQRSDVWWQHVLTSWTDCEWKDNFRMGRAAFLQLCNRLQPH
IQRQTTTFRKPVPVEQRVAICIWRLATNVEFRTISHLFGIGQSTAVSITNCVASAIVKNL
LSIFIRTPSEQEFESIIQGFRDKWGFPQCGGAIDGTHIGILAPPVSSADYYNRKGFYSVI
LQGVVDHRLMFWDINVGWPGKVHDARVFANSSLFDRGQGNSLFPPNIERFGDVDVPVMLL
GDAAYPLMPWLMKPYPENQLTTPAQSTFNNRLSKARMTVERAFGPLKGRWRCLMKRCDCH
IDNINSIISACCVLHNYCQ
>Harbinger-1_SS
PFLEEPVDIEAQILRRNLRRERVIRPRLDVLSFPDDYLFERFRFSAQSIIHLNNILSPHI
VHMTHRGHALSSEQILCVALRFFANGSFLYNIGDAEHISKATVCRAVRNVTLALKRLLYT
FVVFPSHRPTRLIKEEFHRIAGFPGVIGCIDGTHIPITAPSVNEGDYVNRKSFHSINVQI
ICDAAHIISNVEAKWPGSVHDSHIFRECTLSARFARGEFDGYLLGDRGYPCQPYLLTPYP
DPEPGPQQRYNLAHCRTRARVEMTIGMLKARFQCLRRLRVTPERACDIIVACVILHNIAT
>Harbinger-1_TR
MPNTIGVIDCTHIHIQAPHEREWEFVNRKGRHSINVQLIGNADLQIINCVVRWPGSVHDS
RILRESHIYHRFQQNTPDGILLGDSGYPLLRWLMTPFATVTSNSQQNFNYAHTSTRGTIE
RIDGILKRRFACLNYLRVEPKEACNIICACIASHNIAQ
>Harbinger-1_LC
WLARRERRRRQRRLARAMAPHGGLRASLDGGGMSDEECLAMFRLDREGFREVVALVRPAM
QPRLWRADTLGVEVKVAAALCWLATGSFQRVAGHVVGISQPSMSRALTQFVDAMVARARD
YIVFPSTPQERARIQQGFYDASGMPGVLGAVDCTHIGIIAPREEPLLYMNRKGYYSVNVQ
VVCDAECNILNVVAKFPGSSHDSHVWRHCGLSRVLSGLPYGSGHLLGEARCVLWTLHLYI
GAVRMHLSLRGFDSPSHGEDGDADSIRSCVSGDRAYPLRPWLLTPYLHPRGRGQRRYNRV
HTRAR

>Harbinger-3_DR
MAVLALLEDIVNGRIRRERVFRDHGDFLAHDDDWLISRFRFPRAILLDLCAELGPLLERE
TARSHALPVPLQVLTTLGFLATGSFQRELADRSGLSQSSLSRAMPAVWDGIIRMSSRYIR
FPYHAVDQPNIKAQFAAIAGFPNVIGAIDCTHIAIKAPSEDEFAYVNRKHFHSINVQIIC
DAQMRLTNIVARWPGSTHDSFILTNSMVGMRLQGGRVRDGWLLGDRGYPLKTWLLTPLNN
PQTDQERRYNDAHSHTRSVVERAIGQLKCRWRCLDKSGGVLLYRPNKVCRIVLACGVLHN
VAH
>Harbinger-2_OL
LYADGQSDTRPDFRLTRRTIEHLVEQLRVPHTLGGAQETEVLVFLFWLACGTSYGVVARA
FDMPRSTVSDVVHRTADRILQLMSRVTQLPSGNELPLVASGFXQLAWSSAFQKVAGSIDG
CHIRIKALKENAACYFNRKLFYSIQMQAVCDSNAKFIDIFVGNPGSVHDSRVLRKSPIYV
NQTLPT*RILLGDGGYPCIAHPITLLTPYREPMRNAVEARYNQHHXRTRSVVERAFGMIK
MRRRAIFLGALEVKPTFAMKIIACSSILHNLC
>Harbinger-1_NF
LNQSRRDEGEYSSLVRPMRGIDEQSHFKYFRMSANRFDDLLHRIRPHINHAATHSSPVKL
EERLAVTLRVLASGNSQQSVAQSYRLGSTTVSLIVTEVCEALWSALCSDFVSLPDKNKWA
DISQEFWRVWNFPNCLGCVDGKHVIIKAPPNAGSDFFQLQRNSFPRAHGCV*C*LQI*HG
GRRGIWARE*WWSFSGVHVREQAAPWNPGPTTPGQPAWFHNYCSTRVPSGDAAFPLHQNL
MRPFPGM
>Harbinger-4_GM
CTELSPGLERQTARSHALPVPIQVLTTLGFLATGSFQRELADRSGMSQGALSRAIPPVLN
GIIRISARYIRFPYDAVNQANIKAQFAAIAGFPNVIGAIDCTHIAIKAPSEGEYEYVNRK
HFHSLNVQIICDAQMRLTNIVARWPGSTHDSFVLRNSSVGNRLEAGRVCDGWLIGKQ*GD
SGYALRPWLLTPLANPQTVREQRYNDIHARTRTVVERAIGQLKSRWRCLDRSGGMLLYHP
EKVCRIVQACGVLHNVAH
>Harbinger-1_BF
MLGFYDTLMNELMREHRCDFKSFVRMEPQMFYDLLMRVGPSIEKSRNVRPALPAALKLAI
TLRFLATGNSYQSLEFAFRVAHNTISQFIPQVCKAITTEYGDEVFKTPSTPDEWRQVAQG
FQKRWQFPHVCGAIDGKHVRIRKPKKSGSVYFNYKGFFSIVILALADHNYKFLWANVGSP
GSNSDCGIFNNCSLEPSLSGGTIGFPDADPITNDDRDTGYFIIGDDAFPLRTYILKPYAQ
RYLTIEERIFNYRTSRARRVVENAFGIMAMRFRCLLTTLSVAPETATRITEACLTLHNLM
R
>Harbinger-3_BF
AALLVAEGGEPVHRGVWQHPRNPASFWDQIVLDTLSDAEWYKRFRMRKATFQMLCDELDP
ELRHKDTRMRDAISVQKRLAIGLYWLASGDLMRSVADLFGVSVSSACNIIHEVHNAINEV
LFRRYLTFPTGQKLRETIHGFMEKWQFPQCAGAVDGSHIPINAPTKDPTDFFNRKGYHSV
ILQGVVDHLYRFTDICVGRPGSVHDARVLRDSPFFTRMESGTMLPRELTREIEGVPVPVA
VLGDAAYPQMPWLVKPYPDSGALSREKFDFNYRQSRARMTVECAFGRLKGRWRCLSKRLD
VDLDNVPSIVAACCVLHNVCE
>Harbinger_7_DR
MLDPQTCNAMIVPIIRSQETPATRNAQSERHPEKDVLHDCVALLGCFAAIRTALGGPDPK

GRQAREKLSSTVCRCVDSLWTGRHSFARSGCRSAGGTLMNSIRKVSEAKRIAFASASSPP
KGNINGVEVPVMLLDDPAYPLRNWLLKGYTDSGNLTEEQRSFNERHSRARMTVECTFGRL
KGRWRCLGKRLDVDISNVPTMISACCMLHNLCEK
>Harbinger-8_DR
MNRLREKEGEFSLLVRQLRSMDEEMHFKYFRMSAHRFNDLVRRLKPLIVHQCTHSMPVDI
EKRLAVTLRILASGGSQQAIAASYKLSSSAVSAKVSEVCKALWKALQPEYLPCPSVAQWE
NISADFWRMWNFPNCVGSIDGKHVNKKAPPHAGSDFNYKGTHSIVLMATCDARYRFTMVD
VGGYGRESDGGIFKESRFGSMLLENELNLPPPSKLPGTEVQVPHVIVGDAAFPLHCNLMR
PFTGM
>Harbinger-2_GM
MADLALLEDLNARLRRERVFRDRADFFMESDEWLLSRFRLPRHLLMELCNALEPQLRRET
RRSNAIPVPVQVCSTLGFLATGILQREIGDRSAISQPSISRTMPAVLAAIMSLSERYIKF
PYNNDQQTGIKRDFYAIARFPNVIGAVDCTHV*PSINDYAYINRKSYHSVNVQIICDARM
SILNMVARWPGGTHDSFIFQNSNVGHRLHQGALHGQSHLGELRCSRTXPASSRFLLGPLH
SRN
>Harbinger-2_SS
GRDQPRRKRRSVWVQRWLKSRKQAGEFHRLIQELRLFGEEFRSYFLDRSQFDHLLQMVGA
RIARMDTNYRESISPVERLAICFKFLATGDSYRTIGFSFRVGRSTVAGIVPSVAQAIWDC
LVGEYMPVPKEEDWRAIAAEFLERWNFPNCLGSIDGKHVVIQAPPCSGSQFYNYKGTYSV
VLLAVVDAIYCFRVVDVGAYGKGSDGGTLRDSAFGQALQDGTLEIPPPASLPGAEDLGPV
PHVFVGDEAFPLRPNLMRPYAGRQLPLPKPVRIFNKRLSRARLVVECAFGILAARWRMYR
RVLGLSPSNVDACVKATCVLHNYLR
>Harbinger-4_BF
MAELRNEDPTAFHIFMRMPIAMYDELLERVTGRLTKKATFMRDPLDPGLKLALTIQHLVS
GNTYASMKFSWRVPKCTISLVVREVCEAIIATYLDELMVCPTYTPNQWRQIADRFYQKWN
FPHTCGAIDGKHVACQGPWNSGSMYYNYKGFYSISLMALVDADYRFIWADIGGLGSASDA
QVFSQVFNASELKECIEDGTIGFPDPEPLPNDTQDVPFFIIGDDAFSLRTTMMKPYSGRG
LAREERIFNYRVSRARRVAENAFGILARRFRVLLTTMQHHPSTVKLIVTTCVLLHNLMR
>Harbinger-4_DR
MACPFLEDPVDEEAVLIRRELHLRRARILRPRVDFLSFPHTFLFERYRFSLQSITYIHNL
IRPYISNITHRGRALTSEQILCAALRFFANGSFLYNIGDAEHISKATVCRAVRKVCLALK
RFLHIFVVFPGHKPLGVIKEEFYRIAGLDSPMWLDALTAHIYLSLHQLKMSQTMLTGSQS
TVLMCRYVELCSLNCLNNTYPQYYISYLILSTVKIICDAAHIITNVEAKWPGSVHDSRIY
RECTLSNRLQSGKLMYELHTWAFCMNYTLGHFNAALNQCVFFFFTGEIDGFLLGDRGYPC
QPTLLTPYPEPEQGPQQRFNVAHCKTRGRVEMTIGLLKARFQCLRHLRVTPERACDIIVA
CVVLHNIAI
>Harbinger-2_NF
LPDEGKFREIASYFESRWGLPQCVGAIDGSHIPIIAPRNFHTDYFNRKGWHSLILQAVVD
GKGLFWNVFAGLPGSMHDARVLRLSSIWDLASRGNLFPDHSIQIADVDFGYCILGDSAYP
LQDWLLKPFTDTGRLTEQQLLFNKKFSRARVVVENAFGRLKGRWRCLLKRNDCDVSLVRS
MILTCCALHNLCE

>Harbinger-3_OL
MAYSEAVLSTSIERNVWVRSRSQEWWDTDVSGFTDAEFISNFRMTRSTFNYVCERLRLTL
SREETCLRRPITVQKRVGVGLYWLATGACYRTVANLFGIARSTVCSIVREFCEAVRRVLM
PQYIKLPKGGDLQEVIEGFQQRWGFPQCGGAIDGSHIPIIAPEDNHTEYFNRKGWHSVLL
QGVVDHRFCFTNIYAGWPGSVHDARVLRNSHVYSLAERGELFPPGTEEIMGVQVPIMLLG
DPAYPLRSWLLKGYCDTGTLTAEQRNFNVRHSRARMTVECAFGRLKGRWRCLAKRLDVDV
SLVPTIIGACCTLHNLCE
>Harbinger-2_BF
AALALARGGDPINRTIWSYVRLPATFWQKFVQGTLCDAEFYKRFRMTRDTFQMICDKLNP
VLEKSDTRMRLAIPTKKRLAICIYWLASGDLMRSVADLFGVSEGSVSLIVHKVCDAINEV
LFRDYLSFPTGQKLKETIQGYKERWGFPQCAGAIDGSHIPIKAPSKNPNDFYNRKGFYSV
ILQGVVDHMSRYV*VQRFTMIDIGMPGSVHDARVLRESNVFDRGESGTLLPQ*EFAQDIN
GVTVPAVLLGDAAYPTLPWLMKPYPDNGALTREKFDFNYRQSRARMTVECAFGRLKGRWR
CLTKRLDVLLDNVPDIVGACCVLHNICE
>Harbinger-1_GA
MLCVALRFFANGSFLYNIGDAEHISKATVCRVVRKVCLALKRFLRIFVLFPGHKPVRAIK
EEFHRIAGFPNVVGCIDGTHIPIIAPSENEADYVNRRSIHSINVQIICDAAHLITNVEAK
WPGSVHDSRMYRESTLSNRLETGEIDGFLLGDRGYPCRPTLITPYPEPEPGPQRRFNVAH
CRTRARVEMTIGLLKARFQCLRHLRVTPERACDIIVACVVLHNIAI
>Harbinger-6_DR
ALANCSRFKRRKPYLERRFWVTPGGTSAWWDMFVSEVVVPEDWHKNFRMSRSGMIHLSEK
LRPFVEGQTTRLRAPVDVLTKVACTLYYPSDEGRIRKTANAFGLSRQVVSTIIRQVCKAI
TTTLGPEYIKAPKTEAKVNELVANFYRTHGMPQCLGAIDCTHIEIRRPLSNSTDFINRKG
KRVNILKGKKGKYSLNIQALCDYKYRFMDVVVKWPGSVHDARIFANSQLSSDLKDGTIPP
CPKK
>Harbinger-1_TF
MPNTIGVIDCTHIHIQAPHEREGEFVNRKERHSINVQLIGNANLQIINCVVMTPFATVTS
NSQQNFNYAHTSTRGTIEHINGRLKRRFACLNYLCVEPKEACNIICACIVLHNIAQ
>Harbinger-4_LC
WlarrerrrqqrrlgmalrgGVRARLDTGGMSDEECLAMFRLDREGFREVVALVRPAMQP
RQWRADTLGVELKVAAALCWLATGSFQRVAGHMVGISQSSMSQALDQFLDAMVARARDYI
VFPSTPQERARIQQGFYAASGMPGVLGAVDCTHIGIIAPREEPLLYLNRKGYSSINVQVV
CDAELNILNVVSRFPGSAHDAHVWRHCGLSAVLSGLPYGSGHLLGAYARXVLWILHLYST
AVRVYLSVRGF*RPADA*AGDANSMLSCVSGDRAYTLRPWLLTPYLHPRGGGQSRYNITH
ARAR
>Harbinger-2_LC
ATGSFQRVAAQVVGISQSSVSTALQQFLDAMVQHTTTYIAFPHTDQQWANIMQRFFEASG
MPGVLGAVDCTYVPIMAPHQEPVCYLNHKWFYSLNMQVVCDSNGLITDMVARFPGSAHDA
FIWSQCGLHRQLHKLPPGSSFLVGK
>Harbinger-3_LC
WLAWRERRRRQRRRASAMAWRRVPRVRLNVGGLSDEECLARFRLDREGFREVVAIVRPAM

QPHTWRGGPLGVELKVAAALCWLATGSFQRVAGQVVGIXQSTMSAALTQFLDAMVVRATD
YIVFPSTPQERERIQQGFYDASGMPGVLGAMDCMHIGIIAPREEPLLYLNRKRYYSMNVQ
VVCDADAHILDVVARFPGSTHDAHIWSQCGLSRLLSGLPEGSGHLLGELGWLSPHTLYKH
RGVVRMYPPRGAPTAPGTGRMGMLTACTHVLQVIVHTHCGH
GS
>Harbinger-2_DR
MAALQRVFQLRVRQRERQRQRQRRPRSTLCTINAFIRQHQNPLDMLDDMAVIHRYRLPRG
EIVQLLNVIGPQLMRATRRNFALSPDVQLLAAVRYYATGSFLQVLGDGLGLSKPSVSRAV
QAVTYALLPLAAEHIKFPASRQAMSDIQEYFLTHYHIPQVIGVIDGTLIPISTPSVDGHT
YICRKGYPAINCQVICDHNCLITDIVARWPGSTHDSYIFTNSSVGQEAQNSNGHWRLLGD
SGYPLRPYLFTPVANPVSNSEAHFNEAHRVARSTVERTLGRWKLRFRAIHKSSGGLLFVP
QKCCAVITVTAMLHNIAV
>Harbinger-3_GM
WKRERRRHARCLWVHETIRSRTELGEFH*AQFDDLLARIGARISRLDNNYRRSISASER*
SGFI*RYLATGDSYRTIANSFHVGISTVSSIVPDVATAIDSLVEEFMAVPSTDEWTSIAG
RFEERWNFPLCCGPLDGKHVVIEAPAHSGDQFYNYKGTFSLVLLAVVDAEYCFRVIDVGV
YGRTSDGGILANSAFGAGLQSGTLQLPADLPLPGADHRGPQPHVFVADEAFPLRKNLMRP
FPGRNLARERRVFNYRLSRAWMVVENAFGILSSQWRVYRHAFEVNVEVAEKCV
>ISL2EU_OL
QVPLLEPGDDVMADKEFLVQDLLTDIGSKIIIPPF*
SSISKEETEQTAAIARLRIIVERVIGRIKSFHIRDSPVPLTLMGSVNQIWLNCRVLANYH
>ISL2EU_SS
SRVISYWIDMMEENLREYIPWLPRETIRATMPQCFKDHYPGTTCIIDCSETALQKCKNLD
SRGESFSHYYAQNTIKYLVAIAPCGLVMFISSAYGGRCSDKYITSDSGFLEYLCPGDEVM
ADRGFTIRDLLHERKVK
>ISL2EU_ON
LLDENRLLRSQLQKTELNENXLKDDTEKVRYYTGLXCYAIXMSLFDTVKDFLPISKKLTG
FQmllltlmrlrldlPVQHLCHLFDVSRKTLSSVFADTVDVLYARLGALVHWPKRRCXQA
TMPXQFIETFGNRVAVIVDCFEIYTERP*NLKARAETYSHYKXTHTMKYLVGITPQGAIS
FISKGWGGRASDKHITERCGILXKLSPGDVVLADRGFDIRDAVGMTCAEVKIPAFTRGXC
QLDAKDVEXTRAIAHLRIHVERVIGSLRNKFKMLHTTMPI*DXLVPCEGEDATLLDKIVK
VCCVLVNMCPSVV
>ISL2EU_DR
VLYLSGHLDAAAQRIRELEAKVRQLKSNSVIYLPQNYTLTDTVQXTRTLDLGSHLRKYSG
YFGNQLPHQPPDAXPVLLXCCCWPKREGYSRHFWHQHRHCSRVIITWANYLYLVLGSLPI
WMSREQVNIRLALMPAKFAEYCPDLRVIVDCTEIRXESPSSLTLQSETFXVYKNHTTFKA
LIGIAPCGVITFVSKLGSISDQEITKQCGILQLLEPGDACMADKGFVIDQMLSDVGACLI
IPPFKRGACFSKENTEKTQSIARLRILVERAIRRIKEYHIWDTTVPLTLSGSVNQLWTTC
CLMSNFQYPLDTKDHICV
>ISL2EU_XM
ILDCTEVELEMPSFLTLQSEMFSAYKNHITLKGLIGVAPCGMVTFISALYRGCISDKEIT

KVSGILPLLELGDEVMADKGFLIQDLLHEVGAKLIIPPF-RHPGQFSKKETEETQTIARL
RIIVERAIARVKCYHIWDSPVPLTLIGTVNQIWHNCCVLANYQGPF
>DR_copy1
FQMLHHYYTNILRTSEVWAYSRTSDWWENTAQKYTDDQWLEDFRVSRETFRYICSTLKPS
LQRLDTSFRLCIPLAKRVAIALYKLASTTEYRTVANLFAVSRTSVCRCVHDFCKAVIAVL
RPKLINTPDQAKMAEIADYFEDKFGIPQCVGAIDGSHIPILKPPQYQSDFHNRKGWHSII
LQAVVDGKGLFWDLNVGQPGRKHDASVLKKSCLWTWATSTTAFPGRVKNICGTEVGYFIL
GDSTYPLQKWLLKPYPDTGRLTEAQELYNMRTSRARCVVEHAFGRLKGRWKCLSKRNDCN
VNVVVDMVETCCTLHNLCE
>DR_copy2
QECLHNFFSLVVYFCRTIWVFPRSCDWWKYINNTWTDHEWLQNLRMQRSSFILLCDTLRP
WLTRQNARYRKPVPVEIRVAICIWRLATNLEYRSISHLFGVGVSTCCIITQEVVTAINVI
MKPQYIKKPSAAEFKMIVQGFRDRWGFPQVAGAIDGTHINIKAPSNTPADYYNRKGNYSI
VLQAVVDNKMKFWDINVGQPGKVHDARVFCLSSLFDRGSSGTLLPTWTETFEAIDVPLFL
LGDSAYPLSHWLMKPYPEGRGVTPEQIKFNHRLSQARMTVERAFGRLKGRWRCLLKQCEA
HITLVSRIVSACCVLHNFCE
>DR_copy3
FKNVYIYFFSLVVYFCKTIWLFPRSCDWWKYINNTWTDHEWLQN
LRMERSSFILLCDTLRPWLTRQNTRYRKPVPVEIRVAICIWRLATNLEYRSISHLFGVGV
STCCIITQEVVTAINVIMKPQYIKKPSAAEFKMIVQGFRDRWGFPQVAGAIDGTHINIKA
PSNTPADYYNRKGNYSIVLQAVVDNKMKFWDINVGQPGKVHDARVFCLSSLFDGGSSGTL
LPTWTETFEAIDVPLFLLGDSAYPLSHWLMKPYPEGRGVTPEQIKFNHRLSQARMTVERA
FGRLKGRWRCLLKQCEAHITLVSRIVSACCVLHNFCE
>DR_copy4
FAALLSDYSSRTSIRTTWIRQRSDVWWQHVLTSWTDCEWKDNFRTGRAAFLQLCNRLQPHI
QRQTTTFRKPKPVEQRVAICIWRLATNVEFRTISHLFGIGQSTAVSITNCVASAIVKNLLS
VFIRTPSEQEFESIIQGFREKWGFPQCGGAIDGTHIGILAPPVSPADYYNRKGFYSVILQG
VVNHRLMFWDINVGWPGKVHDARLFANSSLFDRGQGNSLFPPNIERFGDVDVPVMLLGDAA
YPLLPWLMKPYPEN*LTAPAQSTFNNRLSKARMTVERSFGLLKGRWRCLMKRCDCHIDNIN
TIISACCVLHNYCQ
>DR_copy5
MAVLALLEDIVNGRIRRERVFRDHGDFLAHNDDWLCSRFRFPRAILLDLCAELGPLLKKE
TARSHALPVPSQVLTTLGFLATGSFQRELADRSGLSQSSLSRAMPAVWDGIIRMSSRYIK
FPYHAVDQPNIKAQFAAIAGFPNVIGTIDCTHIAIKAPSEDELAYVNRKHFHSINVQKYV
MLKCA*QILWQGGLGQPMIRLPLQTAWLG*GSKVAGYVMGGFLVSDVFEDIIPANFLFNC
ISGDRGYPLKTWLLTPLNNPQTDQEHRYNDAHSLTRSVVERAIGQLKCRWRCLDKSGGVL
LYRPNKVCRIVLACGVLHNVAH
>DR_copy6
RAQQRPARVLLDRSDPLNFFDDTAFQGRFRMYKQHALEIISLLEPTLSCLSLRGRPLPSS
LQVLLTLRFLASGTFHRETGDLCGVSEATVCRIVHKVCRAICELRSVYIKFPDAADQAKY
KVQFYEYGHFPGVIGCIDGCHVPIKCPSTPDAEEYRNRKNWFSINVQGVCTPNLEFANLV

AHWKGSTHDSRIFLNSSLCAQFQRGQHSGLLLGDSGYGQSTFLFTPYINPTTVEQQRYNR
AHIQTRGMVERMFGIWKNRFQSLRNTLRFSPRRCCKVIIATAVLHNYLK
>DR_copy7
ALANCSRFKRRKPYLERRFWITPGRTSAWWDMFVSEVVVPEDWHKNFRMSRSGMIHLSEK
LRPFVEGQTTRLRAPVDVLTKVACTLYYLSDEGRIRQTANAFGLSRQVVSIIIRQVCKAI
TTTLGPEYIKAPKTEAKVNELVANFYRTHGMPQCLGAIDCTHIEIRRPLSNSTDFINRKG
KYSLNIQALCDYKYCCMDVNVKWPGSVHDARIFANSQLSSDLKDGTIPPCPKKLLDDEEA
VSVFLLGDPAYPLMPYLMKEYPNGGVTPQEQYYGLSLCKARMVIECAFGRLKARFAALRR
AMDINMNDLPFVIYACFVLHNYCE
>DR_copy8
LERRFWITPGRTSA*WDMFVSEEVVPEDWHKNFRMSRSGMIHLSEKLRPFVEGQTTRLRA
PVDVLTKVACTLYYLSDEGRIRKTANAFGLSRQVVSIIIRQVCKAITTTLGPEYIKAPKT
EAKVNELVANFYRTHGMPQCLGAIDCTHIEIRRPLSNSTDFINRKGKYSLNIQALCDYKY
CFMDVVVKWPGSVHDARIFANSQLSSDLKDGTIPPCPKKLLDDEEAVSVFLLGDPAYPLM
PYLMKEYPNGGVTPQEQYYGLSLCKARMVIECAFGRLKARFAALRRAMDINMNDLPFVIY
ACFVLHNYCE
>DR_copy9
LERRFWITPGRTSAWWDMFVSEVVVPEDWHKNFRMSRSLSEKLRPFVEGQTTRLRAPVDV
LTKVACTLYYLSDEGRIRKTANAFGLSRQVVSIIIRQVCKAITTTLGPEYIKAPKTEAKV
NELVANFYRTHGMPQCLGAIDCTHIEIRRPLSNSTDFINRKGKYSLNIQALCDYKYCFMD
VVVKWPGSVHDARIFANSQLSSDLKDGTIPPCPKKLLDDEEAVSVFLLGDPAYPLMPYLM
KEYPNGGVTPQEQYYGLSLCKARMVIECAFGRLKARFAALRRAMDINMNDLPFVIYACFV
LHNYCE
>DR_copy10
MAVLALLEDIVNGRIRRERVFRDHGDFLAHDDDWLCSCFRFPRAILLDLCGELGPLLERE
TARSHALPVPLQVLTTLGFLATGSFQRELADRSGLSQSSLSLAMPAVWDGIIRMSSRYIK
FPYHAVDQPNIKAQFAAIAGFPNVIAAIDCTHIAIKAPSEDEFAYVNRKHFHSINVHIIC
DAQMRITNIVARWPGSTHDSFILTNSMVGMRLQGGRVCDGWLLGDDRGYPLKTWLLTPLN
NPQTDQERRYNDAHSLTRSVVERAIGQLKCRWRCLDKSGGVLLYRPNKVCRIVLACGVLH
N
>DR_copy11
MAALQRVFQLRVRQRERQRQRQRRPQSTLCTINAFIRQHQNPLDMLDDMAVIHRYRLPRG
EIVQLLNVIGPQLMRATRRNFALSPDVQLLAALRYYATGSFLQVLGDGLGLSKPSVSRAV
QAVTYALLPLAAEHIKFPASRQAMSDIQEYFLTHYHIPQVIGVIDGTLIPISTPSVDGHT
YICRKGYPAINCQVICDHNCLITDIVARWPGSTHDSYIFTNSSVGQEAQNSNGHWRLLGD
SGYPLRPYLFTPVANPVSNSEAHFNEAHRVARSTVERTLGR*KLRFRAIHKSSGGLLFVP
QKCCAVITVTAMLHNIAV
>DR_copy12
DDMAVIHRYRLPRGEIVQLLNVIGPQLMRATRRNFALSPDVQLLAALRYYATGSFLQVLG
DGLGLSKPSVSRAVQAVTYALLPLAAEHIKFPASRQAMSDIQEYFLTHYHIPQVIGVIDG
TLIPISTPSVDGHTYICRKGYPAINCQVICDHNCLITDIVARWPGSTHDSYIFTNSSVGQ

EAQNSNGHWRLLGDSGYPLRPYLFTPVANPVSNSEAHFNEAHRVARSTVERTLGRWKLRF
RAIHKSSGGLLFVPQKCCAVITVTAMLHNIAV
>DR_copy14
FQMLHHYYTNILRTSEVWAYSRTSDWWENTAQKYTDDQWLEDFRVSRETFRYICSTLKPSL
QRLDTSFRLCIPLAKRVAIALYKLASTTEYRTVTNLFAVSRTSVCRCVHDFCKAVIAVLRP
KLINTPDQAKMAEIADYFEDKFGIPQCVGAIDGSHIPILKPPQYQSDFHNRKGWHSIILQA
VVDGKGLFWDLNVGQPGREHDASVLKKSCLWTWATSTTAFPGRVKNICGTEVGYFILGDSA
YPLQKWLLKPYPDTGRLTEAQELYNMRTSRARCVVEHAFGRLKGRWKCLSKRNDCNVNVVV
DMVETCCTLHNLCE
>DR_copy15
MLDPQTCNAMIVPIIRSQETPATRNAQSERHPEKDVLHDCVALLGCFAAIRTALGGPDPK
GRQAREKLSSTVCRCVDSLWTGRHSFARSGCRSAGGTLMNSIRKVSEAKRIAFSSPPKGNI
NGVEVPVMLLDDPAYPLRNWLLKGYTDSGNLTEEQRSFNERHSRARMTVECTFGRLKGRW
RCLGKRLDVDISNVPTMISACCMLHNLCEK
>DR_copy16
FQMLHHYYTNILRTSEVWAYSRTSDWWENTAQKYTDDQWLEDFRVSRETFRYICSTLKPS
LQRLDTSFRLCIPLAKRVAIALYKLASTTEYRTVANLFAVSRTSVCRCVHDFCKAVIAVL
RPKLINTPDQAKMAEIADYFEDKFGIPQCVGAIDGSHIPILKPPQYQSDFHNRKGWHSII
LQAVVDGKGLFWDLNVGQPGREHDASVLKKSCLWTWATSTTAFPGRVKNICGTEVGYFIL
GDSAYPLQKWLLKPYPDTGRLTEAQELYNMRTSRARCVVEHAFGRLKGRWKCLSKRNDCN
VNVVVDTL
>DR_copy17
RTATVWSHPREHRWWDVIVPEFTPEEFIQNFRVSRESFEYICRRLRHMLERKDTNFRLSV
PVKKRVAIALCKLATGSEYRYVSQLFGVGVSTVFNCVQDFCSAVIKILVPVHMKFPSPEK
LKEMADVFENCWNVPQCIGSIDAHHIPIIAPEKNPRGYLNRKGWHSVVLQAVVDGNGLFW
DLCVGFSGNLSDARVLRQSYLWSLLSERDLLNHNKVDISGCDVGYYLIGDSAYPLQNWLM
KPFPDIGGLTPQQESFNSRLSSARSVSDLSFKKLKARWQCLFRRNDCKVELVKKMALTCC
VLHNICE
>DR_copy18
RPTRVWSNIESTDWWERVVMREFQPSDWLEKFRMTKETFFLLCGKLKPRLNRQDTRLRPA
LPLEKRVAVALWRLASNVEYRTISTLFGVGRSTVCKCVRDVCHAIVLLLRPLYLRTPSEQ
ELEDAARLFATRWGFPHCVGAVGSLHVPIIAPSSNTDNYWNSRGWLSVVTQGAVNGLGQF
WDVCAGFPGSTEHSAILQNSTLWARGCDGGFLLRQPPLDFMGHPLGFLMLGDAGYPLKSW
LLKGYPESSALTAGQRAFNRRLERARSVVDQAFLRLRARWQCLLKRNDCRMDVVPTMILA
CCVLHNVCE
>DR_copy19
IQR*TTTFRKPVPVEQRVAICIWRLATNVEFRTISHLFGIGHSTAVSITNFVASAIVKNL
LSLFIRTPSEQKFESIVQGFRDKWGFPQCRGAIDGTYIGILAPPVSPAD*YNRKGFYSVI
LQGVVDHRIMFWEIIVRWPGKVHDARVLANSSLFDKGQSNALFPPNLLRFREVDVPIMLL
GVAAYPLLLWLMKPYPENQQTTPAQTTFNNRLSKARMTVKRPFGLLKG
>DR_copy20

LLLPLRIIQSPSPTEEDLPNHVPIPSDPDWWERVVMTEFGPEDWLDKFHVTREIFLLLST
KLKPQLEQVYIQQTQQTITLEKCIAMALMRLSTSTEYCFLSELFGVPIPAVCQCVREVCE
AIILLLKPIYMQLPAQHELEDNVEQFRSLWGFPHCIGVIDSLHIPVNLPAAESQDCWNPS
GWHSVVLQGVVNAHGNFWDVCSGFTGSTDDMTILQSSELWTMAEKGGFCSQPPKELMGRP
LGFLLLGDVGFSLQNWLLKCYPSSSNLTPQQQTFNVKLNLGLKVIEQAFQRLKARWQCLH
NRNDNNVDLVSKMAVACCILHNICS
>DR_copy21
RTTWIRQRSDVWWQHVLTSWTDCEWKDNFRMGRAAFLQLCNRLQPHIQRQTTTFRKPVPV
EQRVAICIWRLATNVELEQSPIYLVLVSQLQ*A*QTVLPQQL*KICFQFSSAHPLNRNLK
A*FRVSETSGGSHNVEEPLMALILGF*LHQSALQTTITGKVSILQGVVDHRLMFWDINVG
WPGKVHDARVFANSFLFDRGQGNSLFPPNIERFGDVDVPVMLLGDAAYPLLPWLMKPYPE
NQLTTPAQSTFNNRLSKARMTVERAFGLLKGR*RCLMKRCDCHIDNINSIISACCVLHNY
CQ
>DR_copy22
LLLPLRIIQSPSPTEEDLPNHVPIPSDP-DWWERVVMTEFGPEDWLDKFHVTREIFLLLS
TKLKPQLEQVYIQQTQQTITLEKCIAMALMRLSTSTEYCFLSELFGVPIPAVCQCVREVC
EAIILLLKPIYMQLPAQHELEDNVEQFRSLWGFPHCIGVIDSLHIPVNLPAAESQDCWNP
SGWHSVVLQGVVNAHGNFWDVCSGFTGSTDDMTILQSSELWTMAEKGGFCSQPPKELMGR
PLGFLLLGDVGFSLQNWLLKCYPSSSNLTPQQQTFNVKLNLGLKVIEQAFQRLKARWQCL
HNRNDNNVDLVSKMAVACCILHNICS
>DR_copy23
MACPFLEDPVDEEAVLIRRELHLRRARILRPRVDFLSFPHTFLFELYRFSLQSITYIHNL
IRPYISNITHRGRALTSEQILCAALRFFANGSFLYNIGDAEHISKATVCRAVRKVCLALK
RFLHIFVVFPGHKPLGVIKEEFYRIAGFPNVVGCIDGSHIPIIAPTENESDYVNRKSIHS
INVQIICDAAHIITNVEAKWPGSVHDSRIYRECTLSNRFFTGEIDGFLRGDRGYPCQPTL
LTPYPEPEQGPQQRFNVAHCKTRGRVEMTIGLLKARFQCLRHLRVTPERACNIIVACVVL
HNIVI
>DR_copy24
PVLFWKIP*MRRLY*FEENYIYVEQGF*DPEWIFLSFPHTFLFERYRFSLQSITYIHNLI
RPYISNITHRGRALTSEQILCAALRFFANGSFLYNIGDAEHVSKATVCRAVRKVCLALKR
FLHIFVVFPGHKPLGVIKEEFYRIAGGFPNVVGCIDGSHIPIIAPTENESDYVNRKSIHS
INVQIICDAAHIITNVEAKWPGSVHDSRIYRVCTLSNRFFTGEIDGFLLGDRGYPCQPTL
LTPYPEPEQGPQQRFNVAHCKTRGRVEMTIGLLKAHFQCLRHLRVTPERACDIIVACVVL
HNIAI
>DR_copy25
FAALLSDYSSCTSIRTTWIRQRSDVWWQHVLTSWTDCEWKDNFRMGRAAFLQLCNRLQPH
IQRQTTTFRKPVPVEQHVAICIWRLATNVEFRTISHLFGIGQSTAVSITNCVASAIVKNL
LSIFIRTPSEQEFESIIQGFRDKWGFPQCGGAIDGTHIGILAPPVSPADYYNRKGFYSVI
LQGVVDHRLMFWDIYIWPGKVHDARVFANSSLFDRGQGNSLFPPNIERFGDVDVPVMLLG
DAAYPLLPWLMKPYPENQLTTPAQSTFNNRLSKARMTVERAFGLLKGRCRCLMKRCDCHI
DNINTIISACCVLHNYCQ

>DR_copy26
SADLRPDFRLSRQSFNSLMTALGEESDHGWGSVMETLMFLFWLASATSYRVVARAFAIPR
STVHRAIHNTSKKVLRIMKKVIAIPSGDDLPSLGNEFARLAGSAVFNKVVGSIDGCHVRL
KPPSTDSLCYLNRKLFHSIQFQAICDHKGKFLDVFAGFPGSVHDARVLKHSPIYYQRLYP
PPGWCIVGDGGYPCLSQPIKLMTPYRHPLHTPIQQAFNHHLSKARCVVERAFGVLKTRWR
SIFLKALEIDPLYAPEVVACCTVLHNIC
>DR_copy27
RRFWVRPGRCRVWWYNFVCGLVVPEEWRENFRMSRDSLYRLSELLRPHIQGQPMRIRSPV
DVVTKVACTLYYLGVEGRLRKTANAFGLARQTVSKIIKEVCRAHISHFIWALVCFTSIYR
N*GAEACHKLLSNTWDAAVS*SH*LHAYSVEIKQPSAHSTDYINRKNSFSLNIQAICDYK
YYFMDVVIKWPGSVHDVRIFANSKINAYLKYGKIPSCPKVIVEGEDPIPVFLLGDPTYPL
LPYVMKEYASGGNTP*EQYFGLSLCKCRMVIECAFGPLKARFGALRRAMDINLDDLLFIF
NACFVLHNFCE
>DR_copy28
PGRTSAWWDMFVSEVVVPEDWHKNFRMSRSGMIHLSVKLRPFVKGQTTRLRAPVDVLTKV
ACTLYYLSDEGRIRKTANAFGLSRQVVSIIIRQVCKAITTTLGPEYIKTPKTEAKVNELV
ANFYKTHGMPQCLGAIDCTHIEIR-PLSYSTDLINRKGKYSLNIQALCDYKYCFMDVVVK
WPGGGGVHDACIFANSQLSSDLKDGTIAPCPKKLLDDEEAVSVFLLGDPAYPLMPYLMKE
YPNGGVTPQEQYYGLSLCKARMVIECVFESTICGSEKGNGHKYE*SSFCYYACFVLHNYC
E
>DR_copy29
RYLATGNSVRTIAFHCRLGESTVAKAIKDTCAAINRNMALTHLPRPTEEDWRNIASKFRS
R*NFPHCIGAIDGKHIIIFAPPHSGSLYYNYKKSYSVVLLAVVDAEYRFRLVHIGEFGRS
SDGGVFAGSAIGQALDAKKLEVPNDQQLPGAEHLGNMPFTIVGDAAFPLKTYLMRPYPGR
DLTSQQRIFNYRLSRARNVVENAFGILSSRLRILHSRINIHPDKINSLILSACILHNYLL
>DR_copy30
RFLATGDSYRTIAFSYRVGVSTVAGIVAAVTRAIWDTLAQEVMPVPTTEDWRNISTDFLH
RWNFPNCLGSIDGKHVVIKAPDNSGSLFYNYKGTYSVVLLAVVDSQYRFRVVDVGSYGRM
SDGGVLANSIFGQALRDGALGLPQDALLSGAEHFGPQPHVFVADEAFPLRRDLMRPFPGH
NLSGRQRIFNYRLSRARLIVENTFGILTAQWRMYRGAIEISPANVDACVKATCVLHNFLR
>OL_copy1
MAYSEAVLSTSIERNVWVRSRSQEWWDTDVSGFTDAEFISNFRMTRSTFNYVCERLRLTL
SREETCLRRPITVQKRVGVGLYWLATGACYRTVANLFGIARSTVCSIVREFCEAVRRVLM
PQYIKLPKGGDLQEVIEGFQQRWGFPQCGGAIDGSHIPIIAPEDNHTEYFNRKGWHSVLL
QGVVDHRFWMGVQVPIMLLGDPAYPLRSWLLKGYCDTGTLTAEQRNFNVRHSRARMTVEC
AFGRLKGRWRCLAKRLDVDVSLVPTIIGACCTLHNLCE
>OL_copy2
MSGFIDTEFISYFRMTRSTFNYVCERLQLTLSQEETCLRQPITVQKHVGVGLYWLATGTC
YRMIANLFGIAKSTVCSIVHKFCQAVPRVLMPQYIKLPKGDDLQEVIEGFQQRWGFLQCG
GAIDWSHIPIIAPEDNQAEYFNRKGWHSVLLQGVVYYRFW*EI*ILYFYNSLKILYLPHF
LLIEPYICKNIILENTVHKKLYTVQ*FNTSLVPTIISACCTLHNVCE

>OL_copy3
ARRSRRLIFTRVPLAALARHEQLGLPCI
YCLPNIKVPVLANYADGQSDTRQDFRLTRRTINHLVEQLRVPHNQGWAQETEVLVFLFWL
GCGTSYRVVARAFDMPRSTVSDIVHRTADRILQLMRRVIRLPSRAELPLVASGFEQLAGS
AAFQKVVGSIDGCHIRIKAPKEDPASYFNRKLFYSIQMQAVCDSNAKFLDIFVGYPGSVH
DSRVLRNSPIYTNKTYPPEGYILLGDGGYPCIAQPITLLTPYREPLRNAVEARYNRHHAK
ARSVVERAFGMMKTRWRAIFLGALEVKSKFAVKIIACSAILHNMCI
>OL_copy4
GLPCIYCLPNIKVPVLANYADGQSDTRQDFRLTRRTINHLVEQLRVPHNQGWAQETEVLV
FLFWLGCGTSYRVVARAFDMPRSTVSDIVHRTADRILQLMRRVIRLPSRAELPLVASGFE
QLAGSAAFQKVVGSIDGCHIRIKAPKEDPASYFNRKLFYSIQMQAVCDSNAKFLDIFVGY
PGSVRDSRVLRNSPIYTNKTYPPEGYILLGDGGYPCIAQPITLLTPYREPLRNAVEARYN
RHHAKARSVVERAFGMMKTRWRAIFLGALEVKSKFAVKIIACSAILHNMCI
>OL_copy5
QADLALLEDVANGRIRSECIFRQQEDLLANEDEWLMSRFRLLRAVLLDLCGLLGPALQRS
TWRNHTVPVPLQVLKTLGFLATGTFQRELADRSGISQPTLSRVMPDVLKGIKALSRDTTK
FPYTAGEQANKKAQFAAMSGFPNVIGAIDCTHVAIRAPNVNENTFINRKHFHSINVQIIC
DADMLLTNVVARWPGLTHDSFILRHSSVGRRLEAGAIRDGWLLGESGYPLKQWLLTPFLN
PHSAEERHYNMCHSRARAMVERTIGLFKGRWRCLDCTGGRLLYTPGKVCQIVLACAVLQK
PASL
>OL_copy6
LPRIYCLPSIKVSVLALYADGQSDTRPDFRLTRRTIKLWWSSFGFLTLWGGPQETEVLMF
LFWLACGTSYRVVARAFDTPRSTVSDIVHRTAGRILQLMSRVIRLPSRAELPLVASRFEQ
LAGSDAFQKVVGSIEGCHIRIKAPKEDPASYFNRTLFYSIQMQAVCDSNAKFLDIFVGYP
GSVHDSRVLRKSPIYANKTYPPEGYILLGDGGYPCIAQPITLLTPYRESLRNPVEAR*NR
HHAKARSVVERAFGMMKTRWRAIFLGHRKLKQNLPSK
>OL_copy7
RMACSFLEDPIDEEAALIRRELHLRRARILRPRLDVFSFPHTFLFERYRFSLQSITYIHN
LIQPFITNISHRGRALKSEQILCAALRFFANGSFLYNIGDAEHISKATVCRAVRKVCLAL
KRFLHIFIVFPGHKPLRAIKEEFHRIAGSGFPNVVGCIDGTHIPIIAPTENEADYVNRKS
IHSINVQIICDAAHIITNVEAKWPGSVHDSRIFRESTLSNRLEGEIDGFLLGDRGYPCQP
KLLTPYPEPEQGPQQRFNLAHSRTRARVEMTLGLLKARFQCLRHLRVTPERACDIIVACV
VLHNIAILRGE
>OL_copy8
MVCPFLEDPIDEEAALIPREFHLRRARILRPRLDFLSFPHTFLFERYRFSLQSITFIHNL
IQPYITNISHPGRALTSEQILCAALRFFANGSFLYNIGDAEHIRKKATVCRAVRKVCLAL
KRFLRIFIVFLGHKPLRAIKEEFHRISMLNNQMWLDALMAHIYLSLHQLKMRRNM*TESQ
STVLMCRYIELYLKCLILSTVKIKCDAAHIITNVEAKWPGSVHDSQIFRESTLSNRLECG
K*MYALHLHMHLNVPHKQYDVFVFYTGEIDGFLLGDRGYPYHPKLLTPHPEPEQGPQQRF
NLAHSRTRARVEMTIGLLKARFQCLRHLRVTPERACDIIVACVVLHNIAI
>OL_copy9

MSCLFEEHIVDEEARIIQGFFHRKSVKTTYRCFFVSK*LFTGALSFF*RLLNLFHKSFV
AVHI*RDSSGSSAEHREHFMHRPSFFCD*VFLYSVGDAEHVGKATVCRAVRTACLGLKR
LLGYFVQFPGHKPLRVIKEEFHRIAGLFPNVIGCMDGTRIPIKSPSVNEEDNVYRKSMH
SINVQVTIDGSCIIIPINLSSY*LLFPTI*LICDATHLITNIEAKWPGCVHDARIFRVV
IVSQIPPGYKILYNLHHIKCITAIIGHFNGYLLGDRGYPCLPFLMTPYPEPQPGPQAHF
NLAHSRTRARVEMTIRILKPQFQCLHGLRVTPERACDITAACSVLHNIA
>OL_copy10
RMACPFLEDPIDEEAALIRRELHLRRARILRPRLDVFSFPLTFLFERYRFSLQSITYTQN
LIQPFITNISHRGRALTSEQILCAALRFFANGSFLYNIGDAEHISKDTVCRAVRKVCLAL
KRFLRIFIMFPGHKPLRDIKKEFHRIAGSGFPNVVECIDGTHIPIIAPTENEADYVNRKS
IHSINVQIICDAAHIITNVEAKWPGYVHDSRIFRESTLSNRFYTGEIDGFLLGDRGYPCQ
PKLLTPYPEPEQGPQQRFNWAHSRTGARVEMTIGLLKACFQCLRHLRVTPERACDIIVAC
VVLHNIAILRGEQHPALHIQDPEE
>OL_copy11
MADLALLEDVANARIRRERIFRQQDDLLANEDEWLMSRFRLPRAVLLDLCGLLGPALQRS
TRRNHAVPVPLQVLTTLGFLATGTFQRELADRSGISQPTLSRVMPDVLKGIRTLSRESIK
FPYTAGEQAYKKAQFAVMSGFPNVIGAIDCTHVAIRAPNANENAFINRKHFHSINVQIIC
LAGDSGYPVKQWLLTPFLNPHSAEEQHYNMCHSRARTVVERTIGLFKGRWRCLDCTGGRL
LYTPGKVCQIVLACAVLHNVAQL
>OL_copy12
MAELALLEDVANARIRRERIFRQQDDLLANEDEWLMSRFRLPRAVLLELCGLLGPALQRS
TRRNHAVPVPLQVLTTLGFLATGTFQRELADRSGISQPTLSRVMPDVLKGIRTLSRESIK
FPYTAGEQAYKKAQFAVMSGFPNVIGAIDCTHVAIRAPNANENAFINRKHFHSINVQIIC
DADMLLTNVVARWPGSTHDSFILRNSSVGRRLEAGAIRDGWLLGDSGYPLKQWLLTPFLN
PHSAEEQHYNMCHSRARAIVEAPSACLRAIGLFKGRWRCLDCTGRRLLYTPGKVCQIVLA
CAVLHNVAQL
>OL_copy13
REEHGEFYRLIQELK-MYHERFRGYFRMSISEFENLLQQLAPSLTKEQTHYRKTIDPEQR
LAVCLRFLSTGDSYRTIASSFRLGVSTVASIVRETCDALWHCLRDEHLPVPTEEMWRSTA
RRFHERWNFPNCLGAMNGKHIFIQAPANSGSLYFNYKGTFSVVLLALVDADYRFLVVDVG
SYGSNSDGGIFANSGLGKALRDGTLNVSPPSELPGAPELGKLNHVIVADEAFPLKPYLLR
PYPG
>LC_copy1
THTIRIRRLWTKTRSSVLWNTVIEKAFTNFDWMDNFRVSRATFDFICTKLRPTIQRRDTN
MRRAIPVEVRLAMTLWRLGTCCEYSTIEHLFGVSKSTVCIIVRDVCDAIVNILTPEYIHV
PTGEQLQEVMNGFEECFGLPQTAGALCSTHIPILPPRENPNDYYNEKGWHSVIMQAVVDH
QYCFWDLNIGWPGKADCTYVLLNSELYGRAMKGTLFPSTVRNIGGVDVPIHLLGDLAYPM
LPWLLKPFQENGQLTPEQEDFNCRFTSARTVVENAVGRLKGRWRCLLKRNNIDLSFLPTV
INACCALHNICE
>LC_copy2
TRAWTNTKSNDWWERVVINEFQPQDWLEKFRMSKDTFFHICDKLKPKLSRQDTHLRPALP

LEKRVAVALWRLATNVEYRTISVLFGVGRSTVCKCVREVCHAIVSLLKPQYLRQPNEQDF
ENIVRVFNARWGFPHCAGAIDSLHISIIAPPQYNVDYWNNKCWHSVVVQGVVDGLGQFWD
VCAGFPGSTEDVTILQNSNLWAMASEGGLFPDPPKAFMGKPLKYLLLGDAAYPLQDWLLK
SYSESEGLSKSQLNFNYRLNRAHSVIENAFLRLKARWQCLLKRNDCSLDLIPTMILACCI
LHNICE
>LC_copy3
FRLDREDFREVVAPV*PTMQPRLWRADTLGVEVKVAAALCWLATGSFQRVAGHVVGISKP
STSRALNQFVDAMVACARDYIVFPNTPQERACIQQGFYDVSGMPGVLGAVDCMHIGIIAP
REEPLLYMNRKGYYSVNVQVVCDAECNILNVVAKFPGSSHDSHVWRRCILWTLHLYIGAS
SHGEDGDADSIHSYISGDRAYPLWPWLLTPYLHPRGKGQSWYNRVHARARNAVERCFGVL
KMRFRCLDRTSGSLPLAPGKVCKVFLICCMLHNI
>LC_copy4
AGGMSDEECLAMFRLDREGFREVVALVRPAMQPHLWRADTLGVEVKVAAALCWLATGSFQ
RIAGHMVGISQPSMSKALNQFVDAMVAHARDYIVFPSTPEERARIQQGFYDASGMPGVLG
AVDCTHIGIITPREEPLLYMNRKGYYSVNVQLVCDAECNILNVVAKFPGSSHDSHVWRRS
ILWTWGFDSPSHGKDGDADSICSCVSGDRAYPLRPWLLTPYLHPQGRGQSRYNRVHSWAW
NTVERCFRVLKMRFRCLDRTGGSLPLMPGKVC*AFLVCCMLHNI
>LC_copy5
SDKECLAMFRLDREGLREVVALVQPAIQPRL**ADTLGVEVKVALALCWLATGSFQRVTG
CVVGISKPSMSRVLDEFLDAMVARVRDYIVFSSTPQEQARIQQGFYDASGMPGVLGAMDC
MHISIIAPREAPLLYLNRKGYYSINVQVVSDAECNILNVMAKFPGSSHDSYVWRHCGLWT
LHLYIRAVPSHGEDGDANSIHSCVSGDHAYPLWPWLLIPYLHPRGRGQSRYDRVHTRARN
VVERCFGVLKICFRCLDHTGGSLPLVPAKVCKVSLVCCMLHNI
>LC_copy6
FRALLNTHTIRIRRLWTKTRSSVLWNTVIEKAFTNFDWMDNFRVSRATFDFICTKLRPTI
QRRDTNMRRAIPVEVRLAMTLWRLGTCCEYSTIEHLFGVSKSTVCIIVRDVCDAIVNILT
PEYIHVPTGEQLQEVMNGFEECFGLPQTAGALCSTHIPILPPRENPNDYYNEKGWHSVIM
QAVVDHQYCFWDLNIGWPGKADCTYVLLNSELYGRAMKGTLFPSTVRNIGGVDVPIHLLG
DLAYPMLPWLLKPFQENGQLTPEQEDFNCRFTSARTVVENAVGRLKGRWRCLLKRNNIDL
SFLPTVINACCALHNICE
>XM_copy1
LLMFLYFCLCRTIWVQPRPSTWWAAVSSSWDKVDWKENFRMTKTSFLHLCDILRPRLTRQ
HTNFRSPLSVELRVAICLWRLATNLEYRAISRLFGIGTSTACCVTQQVVTAINVVMKPLY
IKAPSEAELKVIVQGFRDRWALPQVAGAIDGTHISIMAPDENPVDYYNRKNVYSVILQGV
VDNKMKFWDVNVGCPGKVSDAQVLVNSSLYSRGENGTLFPGWIERLNNVDIPLHILGDAA
YPLLPWLVKPFPEGNGLTQPQVNFNYRLSQARMTVERAFGRLRGRWRCLLKRCDAHISFV
SHIISACCILHNYCE
>XM_copy2
NKLVHNFRMSRETFTFLCNKVRPALERETTAFLMNIPLRRVALALWILATGSEYRSIAHL
FEVSIATVCRYVQEFCSAAVSLLVPEQICFPDEGKFREMAELFENKWGFLNCVGAIDGSH
IPIIAPQEYHMDYYNRKGWHSVIL*GGW*GSLLERLHRTAREFTQCQSPVVVYSWELVGQ

GCLLPAEKRRIGAAHVGFCVLGDAAYPLQDWLMKPFSDTGRLGAEKQTFNSKLCRARVVE
NAFGRLKGRWRCLLK
>XM_copy3
SNVRIRTRPWTTTPRSDWWERVVMTEFQPSDWLDKFRMSRETFFYLCEKLRPRLARQDTS
FRLALPVEKRVAVALWRLASNVEYRTISTLFGVGKSTVCRCVRDMCHAIVALLSSTYLRP
TEQELEESAQLFHSYLGFPHCVAAITTLHTAIITPSSNASDYANPAGWLSVLLQVAISGQ
GYFWDVCASFPGGTDPAEILQNSALWATAAEGGLSPAQTPIFMSTVCRYVLLGEACYPLQ
TWLMKAYPEQKSRRGCRAALPEPQQLFNRRLSRALRTSEEALLRLRARWQCLSKRNDCGL
DVVPTMMLACCILHNMCE
>XM_copy4
KMAVLALLEDIANRRIWREHGLRDRTDLLAHEDEWLISQFRLPRAILLELCLELTPTLHR
ETRRNCALLVLVLSTLLATGTFQRELADRSGISHSSLS*AMPAGWDCLMHMLPRYVKFPY
TVAEQANIKLQFAAMSRFPNLVLGTNFKQGPDTMAGF*VVQGYPLATWLLTAFFNRQIEQ
ELRYNELHGRARSVIERTIGQLKGRWCCLDRRGGVLLLHPTEECLIVLACGVLHNVANLH
G
>TR_copy1
MSLHIFSFFCRTVWVRQRSGAWWEQVNANWTDYEWQLNFKMRKTTFQQLCDILTPHLQRQ
MTTFRNPVPVEQRVALCIWRLATNVGFQTISHLFGIGQSTAVTIANDVASAIVNKMLPLY
IQTPSEEEFKLIIQGFRDKWGFPQCGGAIDRAHIGILAPNERPADYYNSKGFYSVILQGV
VDHRLRFWDINVGWPGKLSDATVFGNSSLYERGQSGMLFPHITERFGGESVPVAILGDGA
YPLLPWLMTPYPENQHTNPAQLTFNNHVSKARITAKRAFGRLKERWQCLMKRCDCNINNI
NTVISACCVLHNFCE
>TR_copy2
HSATNERLRRQEVTQYVTAQRQYKPRKYTERPNLLLQ*SDEELNSIYRFGTQGIIYLCDV
LRPHLQRPTVRSHALTVEEQVLIALHFFASGSFFEVIADSIGVTKSTVGCVMHSVARALS
SLLNECVKFPENEEENLQNKKQLFSIAGMPNTIGVIDCTHFHIQAPHEREWEFVNRKGRH
SINVQLIGNADLRIINCVVRWPGSVHDSRILRESHIYHRFQQNTPDGIILGDSGYPLLRW
LMTPFATVTSNSQQNFNYAHTSTRGTIERINGILKWRFACLNYIRVEPKEACNIICACIV
LHNIAQ
>TR_copy3
MMIAGGIRSNVQIRTRPWTTTASTDWWERVVMTEFQPPDWLDKFRMSRETFFYLCDKLRP
RLTRQDTTFRLALPVEKRVAVALWRLASNVEYRTISALFGVGKSTVCRCVRDMCHAIVAL
LSSIYLRPPSGQELHDSAQHCLSSWGFPHCVAAIATLHTAIITPSNNASDYANPAGWLSV
LSQLWFQVVVNGSGQFWDVCASFPGGTDPADILQNSSLWATAAEGGLSPSPLPMFYVILG
EACYPLQSWLMKAYPEEGGRRGRKATLTEPQCLFNQQLRRALRAPEETLLRLRARWQCLS
KRNDCGLDVVPTMILACCILHNMCE
>TR_copy4
MYVDGQTDLRPDFRLSRSTVAKLIDVLRSPFDHGWGLEVEVLVYLFWLASATSYRVVSRG
FSIPRSTVFDIVHRMSDKVLSLKNRTIKFPGLVDIPNIAAGFQRLSGSPALQNVVGSIDG
CHIRIKSPGAHAQCYFNRKLFYSIQLQAVCDHQGLFIDIFTGYPGSVHDARVLRNSPLYV
QGLYPPEGYCIVGDGGYPCMSRPIALVTPYREPVANMMVARFNRHHAKARSVIERAFGIMK

TRWRAIIFKAPEVKPAFATKVTACCTILHNVCL
>TR_copy5
DCGGTGVDCSSFFCKWIEIADSIEVTKSTVGCVVHSVARALSSLLNEYMKFPENEEEISQ
NKKQFFSIAGMPNTIGVIDCTHIHIQAPHEREWEFVNRKERHSINVQLIGNADLQIINCV
VRWPGSVHDSRILRECHIYHRFQQNTPDGILLGDSGYPLLCWLMTPFATVTSNSQQNFNY
AHTSTRGTIERINGILKRRFACPNYLRVEPKEACNIICACIVLHNIAQKRRVPMEEDDIP
LPEGLIEAPQ
>TR_copy6
YIDGQTDLRPDFRLSRSTVAKLIDVLRSPFDHGWGLEVEVLVYLFWLASATSYRVVSRGF
SIPRSTVFDIVHRMSDKVLSLKNRTIKFPGLVDIPNIAAGFQRLSGSPALQNVVGSIDGC
HIRIKSPGAHAQCYFNRKLFYSIQLQAVCDHQGLFIDIFTGYPGSVHDARVLRNSPLYVQ
GLYPPEGYCIVGDGGYPCMSRPIALVTPYRESVVNMMVARFNRHHAKARSVIERAFGIMK
TRWRAIFFKALEVKPAFATKVIACCTILHNV
>TR_copy7
YIDGQTDLCPDFRLSRSTVAKLIDVLRSPFDHGWDLEVEVLVYLFWLASATSYRVVSRGF
SIPRSTVFDIVHRMSDKVLSLKNRTIKFPSLVDIPNIAAGFQCLSGSPALQNVVGSIDGC
HIRIKSPGAHAQCYFNRKLFYSIQLQAVCDHQGLFIDIFTGYPGSVHDARVLRNSPLYVQ
GLYPPEGYCIVGDGGYPCMSRPIALVTPYREPVANMMVARFNRHHAKARSVIERAFGIMK
TRWRAIFFKALEVKPAFATKVIACCTILHNVC
>TR_copy8
FRSESDEFLFGRFRLPRPGLIDLCNQLEPALRSHTLQSNPVPPHVQVLSVFGFLATGTFQ
RELGDRVGISQQSISRALPRVLD*INQLATQYIKFLYPPEYQVTVKRGFHNIAGLPNTIG
AIDCTHVRIKAPSPDPFPYLNRKQYHSINVQLICDANNHLLNVVSTFPGGAHDSFIFQNS
SVGTHLEQGAAGDTWLLGDQGYALAPWLLTPLTNPQTPQEILFNHMHARSHSTIECTIGI
LKGRWMCLDTAGGKLLYKPEKV
>TR_copy9
NLLLQYSDEEMYSRYLFGTQDIIYLCDVLCPHLQRPTVRSHALTVEEQVLIALRFFASGS
FF*SYCR*HWSHKINSGMCGAFCGKSSFKFIK*VCEIS*KLRRNFTKQKTVLSIAGMPNT
IGVIDCTHIHIQAPHEREWEFVNRKGCHSINVQLIGNADLQIINCVVRRPGSVHDSRNLR
ESHIYHRFQQNTPDGILLGDNGYPLLRWLMTPFATVTSN*QQNFNYAHTSTRGTIQRING
ILKWRFACLNYLRVEPKEAGNIICTCIVLHNIAQ
>TR_copy10
MYIDGQTDLRPDFRLSRSTVAKLIHVLRSPFDHGWGLEVEVLVYLFWLASATSYRVVSRG
FSIPRSTVFDIVHRMSDKVLSLKNRTIKFPSLVDIPNIAAGFQRLSGSPALQNVVGSIDG
CHIRIKSPGAHAHCYFNMKLFYSIQLQAVCDHQGLFIDIFTGYPGSVHDARVLRNSPLYV
QGLYPPEGYCIVGDGGYPCMSRPIALVTPYREPVANMMVARFNRHHAKARSVIERAFGIM
KTRWRAIFFKALEVKPAFATKVIACCTILHNVCLQNGDSMEPSEDAL
>TR_copy11
MYIDGQTDLCPDFCLSRSTVAKLIDVLRSPFDHGWDLEVEVLVYLFWLASATSYRLVSRG
FSIPRSTVFDIVHRMSDKVLSLKNRTIKFPSLVDIPNIAAGFQRLSGSPALQNVVGGIDG
CHIRIKSPGAHAQCYFNRKLFYSIQLQAVCDHQGVFIDIFAGYPGSVHDARVLRNSPLYV

QGLYPPEGYCIVGDGGYPCMSRPIALVTPYREPVANKMVARFNRHHAKARSVIEPLL
>TR_copy12
MTALVHVMQQRQRQYKPRQYTERPNLLLQYSDEELYSRYRFGTQDIIYLCDVLRPHLQRPT
VRSHALTVEEQVLIALRFFASGSFFEVIADSIGVTKSTVGCVVHSVASLLNEYVKFPENEE
EISQNKKQLFSIAGMPNTIGVIDCTHIHIQAPHERE*EFVNRKGRHSINVQLIGNADLQII
NCVVRWPGSVHDSRILRESHIYHRFQQDTPDGILLGDSGYPLLRWLVTPFATVTSNSQQNF
NYAHTSTRGTIEHIDGILKQRFACLNYL*VEP KEACNIICACA
>T5_copy14
MAYLLLLQDIADRALRRERMFRDHIDLFAESDEFLLRRFRLTRPVLIDLCNQLEPALRSHT
LRSNPVPPHVQVLSVLGFLATGTFQRVLGDRVGISQPSISRALPRVLDGINQLATQYIKFP
YTPQYQVTVKRGFHNIAGLPNTIGAIDCTHVSIKAPSPDSFPYLNRKQYHSINVQLICDAN
NHLLNVVSRFPLSGDRGYALAPWLLTPLTNPQTPQEMLYNQMHARSRCTIERTIGILKGRW
MCLDTAGGKLPYKPERVCRIIMACCVLHNIA
>SS_copy1
PAEPSDWWERVVTKEFHPQDWLNTFHLTKDTFDLLCDQLRPEVKDPTCHVSLEKRVAMVIL
RLATNLDYLSIGDLLGTSSTTVIKCVRDVCNIIVTVLKPLFIHQPSEQELEEIAAAFNTQW
GFPHCVGVIDSLHISVKSQSQTSDGWNSKGWPSTVVQGVVNGRGHFWDIRVGFSGSTDDAT
ILQGSELWMLAREGGLSPKTPHKLMGQPLGYVLLGDAAFPLQTWLLKCYPESPQLTPQQRT
FNTQLSQARTPIEVTFRRLKARWQCLKRNNSNAALIPVMTQACCILHK
>SS_copy2
MEGPFLEEPVDIESQILRRNLSRERVIRPSLDVLSFPDDYLFERFCFSAQSIIHLNNILSP
HIVHMAHCGHALSSENKFFVLHCFLPRGVFYITSVTLSIFPRQPSVRLSEM*LALKRLLYT
FVVFPSHRPTRLIKEEFHRIAGISLSNNLFIWRLEALISECHLSIVAFIIPNENTSQYIYS
S*LLFCTVKIICDAAHIISNVEAKWRGSVHDSRIFRECTLSARFARCEFIYKAISYYNRYF
VSSYCN*KFKLYPRTIIGKFNGYLLGDRACQPYFLTPYPDHEPGPQQRYNLAHCRTRARVE
MTIGMLKARFQCLRRLMVTPERACDIIVACVILYNIA
>SS_copy3
PFLEESVYIGAQILSRNLSRERVIRSCLDVLSFPDAYLFERFRFSARLIIHLNNFLRPHI
VHMTYRGHGLSSEQILCVALSFFANGSFLYNLGDTEHISKATVCRAVRNVTLTLKCLLYT
FVVFPSHRPTRLKEEFHRIAGFPGVIGCIDGIYIPITAPSVNEGEYVNRKSFHSINVQII
CDAAHISHVEAKWPRSVHDSHICEYTLRARFAREFDGYLLCDRGYSCQPYLLTSYPDPEP
ASQQLYNLAHCRTRARVEMTIGMLKTRFQCLHRLRITPERACDIIVACVILHNIATI
>SS_copy4
MACPFLEETVDIEAQILRRNLSRERVIRPLLEVLSFPDDYLFEHFRFSAQSIIHRNNIPS
PHIIHMTHRGHALSSELILCVALRFFSPTGVSYTTSVTLSIFPRQPSVRLSEM*LLH*NV
YSTLVVFPSHRPTRLKEESIELQVSV*AIIYLV*SFET*STNQLILYIDGTHIPITAPSV
NEGDYVNRKSFHSINVQVGLNIIL*HTK*KILHNTANYCSALRRSCDAAHIISNMEAKWP
GSVHD*HIFCECTLSARFARGEFICIYSYVLL*SIFCFLLLQLNPRIIIGEFDGYLLSDR
GYPIPALFAYPEPGLQQHYNLAHCRTRARVEMTIGMLRAWFQCLRRVRVTPERACDIIVA
CVILHNIA
>SS_copy5

MACPFVRDVVDEEALVLRRAFRRERVFRDRLDPLAFPDDHLYERYRFSADGIRYLCRLLG
PRIKHRTARSHALSVEQMVCVALRFFSSGAFLYSVGDAEQLNKATICRTIRSVCLAIKAL
ADVFISFPGHRRLCDIKEEFYRIAGFPNVIGAVDCTHIRIKAPSGAHEADFVNRKSFHSI
NVQVVCNADCVISNVVAKWPGSVHDSRIFRASEIYQCLSQGKPHNPYLAGRQGYGCQTFL
LTPFTDPQEAQQAYNHAHARTRARVEMTFGLLKARFHCLHKLRVSPVRACDITVACAVLH
NVACL
>SS_copy6
HSFIHLNNILCPHIVHMTHHGHALSSEQIFVFLPTGVFYITSVRLSIFPRQLSVRNVTLA
LKRSLYTCGVPKSQTHKTQRRIPQNCRYILGFPGCIDGTHIPITAPSVNEGDYVNRKLFH
SISVQVVKIISDAAHIISNVEAKWSGSVHDCQIF*ECTLSTGFARGEFDGYLLGDRGYPC
QPYLLTPYPDPEPGPQQRYNLAHCRTRARVEMTIGMLKARFQCLRRLRVTPERACDIVAC
VILHNIATI
>SS_copy7
IISDRLNPVEFNDNDEFSRRYRFSKDPVMQLERKVGPTIKHGSDRNEAVPPLLQLLVALR
FYVTGCFHMVVGDLCGLHKSTVCRIVVSVQCYCRSEESVHKVESYRGNSSWILQESEIPR
CTRGNRQHAFLFPTLVVRTENYSVMVKGYCSINVQAVCDKGQLTNIVARWPGSTHDCRIF
DNSHLCAMLERGAYEGHLLGDNGYACRGCLMTPLLNPQTPEERECNTSHILVRIVIEKVF
GVWRKKRFPCLEGLRTKMDTTLTIIIAVAVLYNFG
>SS_copy9
KRAALAPLEDLANGRIRRE*IFRDQQDLLANDDEWLMSRFRLPRAVLLDFCAVVGPALQR
STRRNQDVPVPLHVLTTLGFLETGTFRRELADRSGISQPTFSCVMPDVLSVIIGLTHLHI
KFPYTVGEQANKKVQFAAMSGFPNVIGAIDCTHVAIRAQSENEFAFIN*KHYHSINVQII
FHVDMILTNVVARWPWSTHDSII*RNNSVGSRLEAVAVRDGLLLGKDEQCRNSKSIAISL
YCSARADSSGRNILYKREKVCNIVRVCAMLHNVAQL
>SS_copy10
MANLALLEDLAQAALCRERVFNERADLFAESDAWLISRFCFPRHILLYLCNQLSPILKRE
TKRTNAIPVHTQVLSTLGSLATGTFQRKIAARSCISQPTLSRILPAVLHGTISLTPQYIQ
FPYTAVQQARVKMDFHSIARFPNVIGAIDCTHIAIKAPSLNKFNFLNRKGFHSVNVQVIC
DSHLALLNVVAKWPDGTHDSVIVQNSSVGLNLQEGAVEDGWLIGGDRGYPLKTWLMTPLT
NPSDQQEARYNQAHATRTTVERTIGLLKARWLCLSGTGGTLQYQPSKVCYIVKACSVLHN
IA
>SS_copy11
FRRERVFRDRLDPLAFPDDHLYERYRFSADGIRYLCRLLGPRIKHRTARSHALSVEQMVC
ALRFFSSGAFLYSVGDAEQLNKATICRTIRSVCLAIKALADVFISFPGHRRLCDIKEEFY
RIAGFPNVIGAVDCTHIRIKAPSGAHEADFVNRKSFHSINVQVVCNADCVISNVVAKWPG
SVHDSRIFRASEIYQCLSQGKPHNPYLLGDRGYGCQPFLLTPFTDPQEAQQAYNHAHART
RARVEMTFGLLKARFHCLHKLRVSPVRACDITVACAVLHNVA
>SS_copy12
FRFLATGDSYRTIGFSFRVGRSTVAGIVPSVAQAIWDCLVGEYMPVPKEEDWRAIAAEFL
ERWNFPNCLGSIDGKHVVIQAPPCSGSQFYNYKGTYSVVLLAVVDALYCFRVVDVGAYGK
GSDGGTLRDSAFGQALQDGTLEIPPPASLPGAEDLGPVPHVFVGDEAFPLRLNLMRPYAG

RQLPLPKPVRIFNKRLSRARLVVECAFGILAARWRMYRRVLGLSPSNVDACVKATCVLHN
YLRRSFQEPLRME
>SS_copy13
GRDQPRRKRRTVWVQRWLKSRKQAGESHRLIQELRLFGEEFRSYFCLDRSQFDHLLQMVG
ARIARMDTNYRESISPVERLAICLFRFLATGDSYRTIGFSFRVGRSSVADIVPSVAQAIW
DCLVGEYMPVPKEEDWRAIAAEFLERWNFPNCLGSIDGKHVVIQAPPCSGSQFYKGTYSV
VLLAVVDALYCFRVVDVGAYGKGSDGGTLRDSAFGQALQDGTLEIPPPASLPGAEDLGPV
PHVFVSDEAFPLRPNLMRPSAGRQLPLPKPVRIFNKRLSRARLVVECTFWILAARCRMYR
RVLGLSPSNVDACVKTTCVLHNYL
>BF_copy1
MAELRNEDPTAFHIFMRMPIAMYDELLERVTGRLTKKATFMRDPLDPGLKLALTIQHLVS
GNTYASMKFSWRVPKCTISLVVREVCEAIIATYLDELMVCPTYTPNQWRQIADRFYQKWN
FPHTCGAIDGKHVACQGPWNSGSMYYNYKGFYSISLMALVDADYRFIWADIGGLGSASDA
QVFSQVFNASELKECIEDGTIGFPDPEPLPNDTQDVPFFIIGDDAFSLRTTMMKPYSGRG
LAREERIFNYRVSRARRVAENAFGILARRFRVLLTTMQHHPSTVKLIVTTCVLLHNLMR
>BF_copy2
MEELRVEDPSSFFNYLRMEPVMFDELVQRVGPRIKKQDTKMR*ALPPSLTLAITVRFLAT
GDNNPSLMYNFRVAHNTISLFIPEVCQAIVEEYKDEVITCPTTPEEWTPIEEVFRERWNV
LHAVGAVDGKHVAIRKPGKSGSLYHNYKGFFSLVLMTLVDGDYKFLWMDISSSGSMSDAQ
IFNDSELKDCLEDGSIGFPEPFPLPQDSEPMPYFLLGDDAFGIRTFLMKPHSHRHLQRDE
RIFNYRISRGRRVVENAFGILVQRWRVLLTTIQPRPSVVQDIVESCVCLHNLMR
>BF_copy3
QGGPQIYRSVWTRERSHHWFQHIVLETYTDIDFYRRFRMRKAIFRFIVTDLQHVLHRSDT
RLRAAIPVPQRIAIALYWLASGDLLRTVADLFGVSAASVCHVVHDVCQAIVDVLLPRYLT
FPSGERLRETVQGYGEKWGFPQCAGAVDGSHIAIKAPVDGRTDFFNRKGYYSIILQGVVD
HLYRFTDINVGWPGSVHDARVLRNSSIFERAERGTLLPQQELAREIHGTNIPAVILGDAA
YPHLPWLMKAYPDNGHLTREQLIFNYRLSRARMTVECAFGRLKGRWRCLSKRLDVDLDHV
PTVVTACCVLHNVCE
>BF_copy4
AGVAIIEAGFVDREIWQRQRSSHWWDVIALTTFTDRDWYSRFRLSRGTFQRLVDELRPSI
EKKNTQMRDAISAEKRLAVTMYWLASGDLFRTVADLFGISEGSVCVIIHDVCKAIVDVLL
PQYIRWPTGERLRANIRGYEEVRNFPQCGGAIDGTHIPVRAPAGEGPDFHNRKGWYSMLL
QGTVDYLYRRFTDISIGFPGSVHDARVLRKSDIFKRGDRGTLFPQDLTKEIDGVQVPVML
LGDPAYPLLPWLMKGYADNGRLDRAKTHFNFRLSSARMTVECAFGRLKGRWRCLAKRLDV
DVGRVPQIVGVCCTLHNICE
>BF_copy5
LQGQYDNLMQELMRESHGDFRNYLRLDAEMFREILDRISPRITKKFYCRPPLSPGLKLAV
TLRFLATGNSYKSLSYSFRIAVNTISLFVPEVCAAIVEEYREEQFATPSTPDQWKVVAEN
FASRWNFPHCCGALDGKHVAMRKPPQGGSHYYNYKGFHSIVIMALVDANYKFLWANVGAE
GSTSDAAIFQLSTLRQGLQEGTIGLPPPDDLPDDDRETPYYIIGDDAFPLREWLMKPFSM
RNLAHNQRIFNYRLSRARRVVENAFGILASRFRVLLTTMNVLQRNAECITKACLVMHNIM

R
>BF_copy6
TVWVRQWLLDRPNYGLYDQLMAQLAEGDVKSFINFMRMEPEMFYKLVEDLTPKIKKKTTR
WREPLPPALRLAVTLRYLATGESYQSLGYAFRVAPNTLVSMVPEVCQAIYDHYHDMVFKC
PTTEEEWKEVAQAFSNKWHFHHCCGCIDGKHVRIQAPAHSGSLYHNYKGFSSVIMLAVVD
ANYRFLYVDMGSYGADSDAGIFRETGLYQALEEDKASLPGSEPLPDGDAAVPYFLVGDDA
FPLRSWLMKPHSKKELSPEERIFNYRLCRARRIVENAFGILANR
>BF_copy7
RTCWVKPWLQRRVLQGQYDNLMQELMRESHGDFRNYLRLDAEMFREILDRISPRITKKFY
CRPPLSPGLKLAVTLRFLATGNSYKSLSYSFRIAVNTISLFVPEVCAAIVEEYREEQFAT
PSTPDQWKVVAENFASRWNFPHCCGALDGKHVAMRKPPQGGSHYYNYKGFHSIVIMALVD
ANYKFLWANVGAEGSTSDAAIFQLSTLRQGLQEGTIGLPPPDDLPDDDRETPYYIIGDDA
FPLREWLMKPFSMRNLAH-NQRIFNYRLSRARRVVENAFGILASRFR
>ON_copy1
FWTRPGRTTAWWDNFESEAVLPEEWRENFRMSRTSFLALCELLRPHIEGQKTEMRLPVSV
VKKVACTLYYLADEGRLRKTANAFGLSRQVVSKIIRQVCTAITVHLGPVFIQLPLTESKV
EELVACFYRAYGIPQCIGAVDGTHVEIKQPSTNSMDYVNRKGRFSLNIQATCDYSYSFID
VVVKWPGSVHDARIFANSTLNTHLKTGRIPSSKKQIVEHEEAIPVFLIGDPAYPLLPYLM
KEYANGGSTAQEQYFGLCLCRARMVIECAFGRLKARFGALRRPMDINLNELPLVIYACFV
LHNYCE
>ON_copy2
CRINLSVPVLDRFFNDDDTRPDFRLSRESVNVLLNHLNQDRRHCCGATIEVLVFAFWLAS
GASYRVVSRVFAIPHSTVHRIVHRVTEEVAAIRHQVIHLPRTPEDLHIVSHGFKELARHR
AFLKAAGAIDCCHVRIKPPSGPDGQ*YRSRKLVPSIILQAVCDHQGRFIDTYVGWPGSVH
DSRALRNSPLYQQAIYPPPGHFILADGGYPCLQHPLPLITPYKRPVQGVGALRFNSHHSR
ARCIIERAFGMMKTRFRAIFLQTQEVHHTFVPHV
>ON_copy3
TMALFGAVAATAVQRYIWTRSRKQEWWDWDVRGFTDTEFIHNFRMSRQTFEYLCQLLSAR
MLRQDTHLRQSIPVSKRVGIGLYWLATGACYRTMSNLFGVAKSTVCSIVHEFCFAVRYVL
MPEYIKWPRGDDLIQVIEGFRQRWGFPQCAGAIDGSHIPITAPEDNHCDYFNRKGWHSVI
LQGVVDHQFWMGVEVPVMLLGDPAYPLRSWLLKGYADTGTLTEEQQYFNQRHSRARMTVE
CAFGQLKGRWRCLGKRLDVDIHTVPTIIAACCTLHNVCE
>ON_copy4
ILSRFLATGDSFRTIAFSFRVGVSTVSQIIPQVATAIWDCLVDNFMAVPSTADWWSIAEG
FQERWHFPLCCGALDGKHVQTKAPPNSGSMFHNYKGTFSIVLLAVVDARYRFCVIDVRGY
GRTSDGGILANSTFGQALRAGTLYLPPDQPLPGEEHRGAQPHVFVADEAFPLRRELMRPF
PGRLLPLEKRIFNYRLSRARMIVEGAFGVLSSQWRLYRRSMELRPEIAEKCVKATCVLHN
FLRCWDERGAPAVRGVAPAVVEPLQGLGRVAANNSSREAVL
>ON-copy5
SDCRKCQTWCPFLEEPVDVEAQILRRALRRERVIRARLDILSFPDDFLCERYRFSAQSII
YLNNILRPYIAHVTHRGHSLSSVHIICIALRFFANGSFLYNIGDAEHVSKATVCRAVRNV

TVALKRLLYSFVVFPGHRPTRFIKQGFHKIAGFPGVIGCIDGTHIPIIAPSVNEGDYVNR
KSFHSINVKIICDAANIITNVEAKWPGSVHDSRIFRECTLSTKFGHGELFTLFAYPLP*P
GPKQRYNLAHCRTRARVEMTMGMLKARFQCLQSLRVTPERACDIIVACVILHNIATI

>ON_copy6
MSCPFVREQPIAEGARLIRSVYRIQRILRDRQDPLAQRDSVLIERYQFSREGIIYLTNLL
DPYVKSSTHRSRALTTAQTVCIALRFFASGTFLYAVGDAENIGRAIRKVYLALKHFLGVF
VVFPSHVRPQVVKQGFFAIAGDSMCHITNVEAKWPGSVHDSRIFRESG*CTLFERGKKKR
LILICHTYFGPDITTFHHYTGAYDGILLGDRGYACRQYFLTPFPDPNPGPQTRYNAALAR
TRARIEMTFGQLKGRFQCLKGLRVAPDRACDIIVACAVLHNIA
>ON_copy7
RGRRDVLQTLDDRELLRRYRLDRAGIMFVVDLLRDAITSPTRRHNAITPETKVITTLRYL
ATGKMQQCSSDDLGLSQSSISRVIPQTLTALSQPNIVTQFVSFPLDARTLHTHKRAFMDI
AGFPGVVGVIDGTHVRIIAPSEDEAVFVNKRNFHSINVQIVFNAACKILDIVAKRPGSTH
DARMLSESGIRQLFERRYVPANCHLLGDSGYPCKPWLLTPYLQPRQGPQLNYNRGPQDNK
SGGGAWHRRFHVLHGEVRLRPEKVSKVIIACAILHNI
>ON_copy8
MANRDRRDVLQTLDDRELLRRYRLDRAGIMFVVDLLRDAITSPTRRHNAITPETKVITTL
RYLATGKMQQCSSDDLGLFQSSVSRVITQTLTALSQPNIVTQFVSFPLDARTLHTHKRTF
MDIAGFPGVVGVIDGTHVRIIAPSEDEAVFVNRRNFHSINVQIVFNAACKILDIVAKWPG
STHDARMLSESGIRQLFERLYVPANCHLLGDSGYPCKPWLLTPYLQPRQGPQLN
>ON_copy9
IMSRFLATGDSFRTIAFSFRVGVSTVCQIIPQVATAIWDCLVDDFMAVPSTADWRSIAEG
FQERWNFPLCCGALDGKHVQMKAPPNSGSMFHNYKGTFSIVLLAVVDARYRFRIIDVGGY
GRTSDGGILANSTFGQALRAGTLHLPPDQPLPGGEHRGAQPHVFVADEAFPLQRELMRPF
PGRLLPLEKRIFNYRLSRARMIVEGAFGALSSQWRLYRRPMELRPEIAEKCVKATCVLHN
FLCCLDDRGAAAVRGVAPAVVEPLQGLGRVAANHSPREAVLVRKKF
>NF_copy1
NQRRQPTVWAFSRASAFWDVTMPNFTPSDWIAHFRMSEETFSYLCSKLRPAMQKRNTNFR
ACVPLRKRVAIALWKLATNSEYRSIGLLFGVSTTSVCRCVQDFCKAVCKLLLAEVIAFPT
LQKLQEMADYFETRWGVPQCVGAIDGSHIPIIAPQGFHTDYFNRKGWHSIILQGIVDGRG
MFWNVNAGQPGSLHDARVLRLSTFWDLVAHGQLHPTSTKNIEGVNVGFYVLGDSAYPLQN
WLLKPFSDNGRLTAEQQAYNRKTSRARVVVENAFGRLKGRWRCLLKRNDSDVELLKHMVL
TCCVLHNICE
>NF_copy2
FFILRYFIFAVTATALLCAFSRASAFWDVTMPNFTPSDWIAHFRMSEETFSYLCSKLRPA
MQKRNTNFRAYVPLRKRVATAMWQLATNSDYRSIGLLFGVSTTSVCRCVQDFCKAVCKVL
LAEVIAFPTLQKLQEMADYFETRWGVPQCVGAIDGSHIPIMAPQGFHTDYFNRKGWHSII
LQGIVDGRGMFWNVNAGQPGSLHDARVLRLSTFWDLVAHGQLHPTSTKNIEGVNVGFYVL
GDSAYPLQNWLLKPFSDNGRLTAEQQTYNRKTSRARVVVENAFGRLKGRWRCLLKRNDSD
VELLKHMVLTCCVLHNICE

>NF_copy3
VWAFSRASAFWDVTMPNFTPSDWIAHFRMSEETFSYLCSKLRPAMQKRNTNFRACVPLRK
RVAIALWKLATNSEYRSIGLLFGVSTTSVCRCVQDFCKAVCKILLAEVIAFPTLQKLQEM
ADYCETRWGVPQCVGAIDGSHIPIIAPQGFHTDYFNRKGWHFIILQGIVDGRGMFWNVNA
GQPGSLHDARVLRLSTFWDLVAHGQLHPTSTKNIEGVNVGFYVLGHSAYPLQNWLLNPFS
DNGRLTAEQQTSNRKTCRARVVVENAFGRLKGRWRCLPKRNDSDVELLKYIVLTCCVLHN
ICE
>NF_copy4
FQMAYLQNTPRRRMVWAIDRSSEWWDVTVPAFTNEQWLQNFRMTEQTFLYLCNKLRPVLE
RQHTSFRMCVPLQKRVAIALWRLATGSEYASIGRLFGVGCTTVCRCVREFCSAAETFLVP
EQIRLPDEEQFRGMAADIENRWGLPQCVGAIDACHIPILSPQHNHNKYLNHNGWHSIILQ
GVVDGNGLFWNVFAGLPGSLDDARVLRLSTLWELASRGNLFPAHTRSVGTMTTGYYILGD
SPYPLLDWLLKPFHDNGRLPSEQQMCNHKFRRVQLVAESAFRKLKGRWQCLLKKNECQVQ
LVKSTVLTCCALHNLCE
>NF_copy5
LNQSRRDEGEYSSLVRPLRGIDEQSHFKYFRMSANRFDDLLHRIRPHINHAATHSSPVKL
EERLAVTLRVLASGNSQKSVAQSYRLGSTTVSLIVTEVCEALWSALCSDFVSLPDKNKWA
DISQEFWRVWNFPNCLGCVVGKHVFIKAPPNAGSGFFNNKGSHSLVLMAVCDANYRFSMV
DIGAYGREGDGGVFQECMFGSSLLHGTLELPPPANLPGSTTTVPHVFLGDAAFPLHQNLM
RPFPGMLS
>NF_copy6
RRRAIWSLNRSSDWWDNYLPRYTEAQWLQHFRMSEETFMFLCNKLWPALRQKDTFFRECV
PLRKIVVIALWKFATGSDYRPISVLFDVGISTAAKCVKDFCAAAEALLLPELIHLPDEGK
FREIASYFESRWGLPQCVGAIDGSHIPIIAPRNFHTDYFNRKGWHSLILQAVVDGKGLFW
NVFAGLPGSMHDARVLRLSSIWDLASRGNLFPDHSIQIAGVDFGYCILGDSAYPLQDWLL
KTFTDTGRLTEQQPLYNKKFSRARVVVENAVGRLKGRWRCLLKRNDCDVSLVRSMILTCC
ALHNLCE
>NF_copy7
LEEPVDVETQILRRHLRRERMIRPRVDVLSLPNEILYKRFRFQAFPFSRSSIICLNNIIS
KATVCRAVRNVTLALKRLVHMFVVFPGHRTTRHLKEALHRTVAISGSQHIARLTHRGDAL
RSDQTFCTAVSFFVNGSFLYHIRDAEHVSGKIIRDADHLISNVEAKWPGSARGSRIFCEST
>NF_copy8
MDALALLDDYVNGRIRMERVFRDHTVFLAHDDDWLISRFRFPRAPLLNLCAELSPVLERP
THQSHSIPVHFQLLTTLGILATGSFQWELTDRSGISQSSMSIIMPAVLNGIIKMTVRYMK
FPYNVREQANIKAQFAAMVIFPNVIGTIDCTHVATRAPSENGYVYVNRTNIHSINVQIIC
GSDMVLTNVVACWPGSTHDSFILMHSSVGNRLQAGAAYDGWLLGEQWLPPRSWLLTPFNN
PQNAEELQFNEVHGCARSVFECAIGYIKCRWRCLDASGGRLLYQPK
KVY*IILACSVLHNIT
>NF_copy9
TVWAFSRASAFWDVTMPNFTPSDWIAHFRMSEETFSYLCSKLRPAMQKRNTNFRACVPLR
KRVAIALWKLATNSEYRSIGLLFGVSTTSVCRCVQDFCKAVCKLLLAEVIAFPTLQKLQE

MADYFETRWGVPQCVGAIDGSHIPIIAPQGFHTDYFNRKGWHSIILQGIVDGRGMFWNVN
AGQPGSLHDARVLRLSTFWDLVAHGQLHPTSTKNIEGVNVGFYVLGDSAYPLQNWLLKPF
SDNGRLTAEQQAYNRKTSRARVVVENAFGRLKGRWRCLLKRNDSDVELLKHMVLTCCVLH
NICE
>NF_copy10
RFWVRPGRTASW*ENFEQEIVLPEEWRNNFRMTRSSLLKLSELLRPHIKGESTVMRSLVC
VTKKVACTLYYLADEGRLRKTVYAFGLSRQVVSKIVQEVCEAITVHLGSLYIQLPTSENA
VYSLVEGFEQMHGLPQCLGAVDGTHVGIKQPSANSTDYLNRKGNFTLNIQATCDYKYCFM
DVVKWPGNVHNARVFANSAINDFLKSQKIPSCPR
>GM_copy1
KYATNVEFRTISHLFGIGHSTAITIANHVAGVIVTKMQPLYIRIPSETDLKDIIKSFRDS
WGFPQCGGAIDGTHIGILAPPNNSSDYYNRKGFYSIILRGVKDHRLRFWDINVGWPKVHD
ASVFGNSSFYERGQSGTLFPNITKSFQGVNVPVVMLGDAAYPLLPWLMKPYPENQQSTPA
QHAFNYRLSRARMNVERAFGRLKGRWRCLMKRYDCRIDINMVISACCLLHNFCEDNSED
>GM_copy2
KRRFWVRPGRTSSWWDNFVNGVVVADEWRENFRMSRASLVALSEELCPYIDGQSTTIRAP
VDTVKKVAVTLYYLSDEGRLRKTANAFGLSRQIVSNVIRETCKAISIHLGPKYIKMPFTE
PEAEDLVLGFHRAHGMPQCLGAVDGTHIEIKQPSRNSMDYINRKGKHSLNVQAVCDFKFR
FMDVSLKWPGSVHDARVFANSKLRSYFKTGKIPALKKQ
>GA_copy1
MLCVALRFFANGSFLYNIGDAEHISKATVCRVVRKVCLALKRFLRIFVLFPGHKPVRAIK
EEFHRIAGGFPNVVGCIDGTHIPIIAPSENEADYVNRRSIHSINVQIICDAAHLITNVEA
KWPGSVHDSRMYRESTLSNRLETGEIDGFLLGDRGYPCRPTLITPYPEPEPGPQRRFNVA
HCRTRARVEMTIGLLKARFQCLRHLRVTPERACDIIVACVVLHNIAI
>GA_copy2
MWAYPRSDHWWDTIVPDFTQFMQNLRVSAGSFEYICCQVKHAMGRRNANYSLCVPIRKRV
AIAMWKLATGVEYRTISHLFGVGLSTVFNCVQEFCNTVITLLLPIHITFPDAEKLLEMAT
FFDNRWRIPQCVGAIDGSHIPVIAPEDYPRDYFNRKGWHSIVLQAVVNGKGLFWDVCVGY
PGSVHDARVLRQSHLWEALSDGELLGQNKVTISGCDVGHYLIGDPAYPMQNWLMKPFCDT
GRLTPEQHTYNYRLSSARSVVEMSFGRLPPKKRRLQAGAEQEDGT
>GA_copy3
RSPVMWAYPRSDHWWDTIVPDFTPLQFMQNLRISPGSF-FSTSAVR*SMPWGEETQTTV*
VSQSERVWLLGFGCGCSHLFGVGLSTVFNCVQEFCNTVITVLLPIHDRCPDAEKLLEMAT
FFNTRWRIPQCVGAIDGSHIPVIAPEDYP*DYFNRKGWHFIVLQAVVDGKGLFWDVCVGY
PGSVHDARVLRQSYLWEALSDGELLGQNKVTISGCDGHYLIGDPAYPMQNWLMKPFCDTG
RLTPEQHTYNYRLSSARSVVEMSFGRLKGRWRCLLKRHDCMLELSKRMALTCCVLHNICE
>GA_copy4
MACPFMDDPVDEEAALIRRELHLRRERILRPRVDFLSYPYSFLFERYRFSLQSIRYIHNL
IHPYIRNISHRRRALTSEQMLCVALRFFANGSFLYNIGDAEHISKATVCRVVRKVCLALK
RFLRIFVLFPGHKPVRAIKEEFHRIAGFLTGEIDGFLLGDRGYPCRPTLITPYPEPEPGP
QRRFNVAHCRTRARVEMTIGLLKARFQCLRHLRVTPERACDIIVACVVLHNIAI

>GA_copy5
PFSEDSQRDSEMSGVVHRRHHHFASRGYRQRVYVERTKPLERYTPEELYVRFRFGKADIE
YLVNLLRPKLQHRTQRSHGLSVEDQILIALRFYACGTFYQVVGDDMGVVKSAVCDVVRDV
SIVLASLVNEFVSFPKDNQIAQAKRSFFLLGNMANTIGAIDCTHVCIQAPRENEWEFINR
KGRHSINVQLVCDADLIITNCVVKWPGSVHDARILRESALYRALQTNRPDGIILGDSAYP
LLPWPMTPFLTANTPVQACFNTAHCRARCAIERLNGRFACLNYLRAEPQKACNITLACIV
LHNIA
>GA_copy6
MACPFMDDPVDEEAALIRRELHLRRERILRPRVDFLSYPYSFLFERYRFSLQSIRYIHNL
IHPYIRNISHRGRALTSEQMLCVALRFFANGSFLYNIGDAEHISKATVCRVVRKVCLALK
RFLRIFVLFPGHKPVRAIKEEFHRIAGDFRMWLDALMAHIFPSLHLPKMKRTMSTGGPST
VLMCRYTESLVFLMVKDYITILMYALHTYWGTSMQL*SNVNFLTGEIDGFLLGDRGYPCR
PTLITPYPEPEPGPQRRFNVAHCRTRARVEMTIGLLKARFQCLRHLRVTPERACDIILAC
VVLHNIAI
>GA_copy7
MDYLEYLNEDQHARQRPARRILLDRSDPLNQFDEITFRDRFRMHKENALEIITLLEPRLS
SLSQRGRPVSNQLQVLITLRFLACGIFHRETGDLCGVSEPTVCRIVHKVSSAICELRSLY
IKFPDAAGQANYKVQFYEYGHFPGVIGCIDGCHVPIKCPSTPDAEEYRNRKNCFSINVQG
VCTPNLEFSNIVARWKGATHDSRIFLNSSLCAQFESGQHSGLLLGDSGYGQSNYLFTPHL
NPTTAEQQRYNRAHIRTRGMIERMFGVWKTRFQCLRNTIRFKPRRCCKVIIATAVLHN
>GA_copy8
MACPFMDDPVDEEAALIRRELHLRRERILRPRVDFLSYPYSFLFERYRFSLQSIRYIHNL
IHPYIRNISHRGRALTSEQMLCVALRFFANGSFLYNIGDAEHISKATVCRVVRKVCLALK
RFLRIFVLFPGHKPVRAIKEEFHRIAGDFRMWLDALMAHIFPSLHLPKMKRTMSTGGPST
VLMCRYTESLLFLMVKDYITILMYALHTYWGTSMQL*SNVNFLTGEIDGFLLGDRGYPCR
PTLITPYPEPEPGPQRRFNVAHCRTRARVEMTIGLLKARFQCLRHLRVTPERACDIIVAC
VVLHNIAI
>CC_copy1
FQMYHILQSRRHRTSVWTFN*ASEWWGVIVPGFTNTQWLENFRMSEETFIYLCDKLRPAM
ERRDTNFRVCVPLKKRVAIALWKLATGSEYRSVGHLFGASISTVCRCVQEFCAAAETLLI
PEQIRFPDQERFKEMAAYIENRWGLPQCIGAIDGSHIPIIAPQDYHCDYFNRKGWHSIIL
QGVVDGKGLFWNVFTGLPGSLHDARVLRLSTLWEVASRGNHIPASTKNIAGVNFGYYILG
DSAYPLQNWLLKPFIDTGRLTAEQRFYNMKISRARVVVENAFGRLKGRWRCLMKRNECAV
DLVKSMVLTCCALHNLCE
>CC_copy3
QEDPRPDFRLSRESLAVLQNLLNQERRDGWGATIETLVFLFWLACGASYRVVSRVFGMPR
STVHYIVHRVTEEVVVIRHKVIFLPKTPEHLEEVSRGFAGLARHRAFIKAAGAIDSCHIR
IKPPSSPDGQCYRNRKLFPSIILQAVCDHQGRFIDTYVGWPGSVHDSRVLRHSPLYAQSS
YPPPGHFILADGGYPCLQWPLPLIPPYKRQVQGVGAQHFNSHHSRARCIIERAFGMMKTR
LRSIFLQALEVHYTFVPHV
>CC_copy4

MACPFMDDPVDEEAVLIRRELHLHRERILRPRVDFLSYPYAFLFERYCFSLQSIKYIHNL
IHP*IRNISHRARALPSEQMLLVCFGLFLFGSFLYNIGDAEHISKATVCRAVRKVCFTLK
RFLRIFVLFPGYKPVRVIKEEFYRIAGMWLGALMAHIFRSLHLLKLTAYCF*WSKTTSQY
CTCLILCTVKIICDAAHIITNVEAKWPSSVHESRMYRESTLSNRLERGK*MYVLYTYFNV
CTTHLQGNFYAALKQCKLTGEIDGFLLGDRGYPCQPNLLPPYPEPETGPQQHFSVAHCRT
RARVKMTIGLLKARFQCLRHLRVTPERACNIVA
>CC_copy5
MACPFMDDPVDEEAVLIRRELHLHQVRILRPRVDFLSYPYAFLFERYRFSLQSIRYIHNL
IHP*IRNISHRGRALTFEQMLCVALRFFANGSFLYNIGDAEHISKATACRAVRKVSKKFL
THNLFLITFAQTTENKPAILIKEEFYRIAGDFRMWLDALMAHIFR*LHLLKMKRYCATLI
TCTVKIICDAAHIITNVEAKWPSSVHESRMYRESTLSNRLERGK*MYVLYTYFNVCTTHL
*GNFYAALKQCKLTGEIDGFLLGDRGYPCQPNLITPYPEPETGPQQHFSVAHCRTRARVK
MTIGLLKARFQCLRHLRVTPERACNIVA
>CC_copy6
FDVFAYPNEYLTERYRFSKDSLVYLTRLLKPHIANVTNRGSALSTENILCIALRFFASGH
FLYSVGDSEHVGKATVCRAVRTVCLALKRLFPTFVQFPGHKPLLVIKDEFHRVAGLFPNV
IGCIDGTHIPMGDYVNRKSIHSINVQVTTVRQPKSSPMSRQKWPGSVHDARIFRESSLCQ
TFQQGQYNGYLLGDTGYPCLPYLMTPYPEPEPGPQTRFNLAHSRTRAKVEMTIGILKSRF
QCLRGLRVSPERACDIIVACVVLHNIATIPNVI
>CC_copy7
MACPFDEDPVDVEAQIIQGFFRRERVIRPRIDVFAYPNEYLTERYRFSKDSLVYLTRLLK
PHIANVTNRGSALSTEKFCASFPGHKPLLVIKDEFHRVAGLSLSVSGFPNVIGCIDGTHI
PMGDYVNRKSIHSINVQVTTFALYNFCFFVFTIFVLYTSIITGQYNGYLLGDRGYPCLSY
LMTPYPEPEPGPQTRFNLAHSRTRAKVEMTIGILKSRFQCLRGLRVSPERACDIIVACVV
LHNIA
>CC_copy8
MSCPFLREQPVAEGARIIRRAFQINRVLRDRQDPLAQRESVIVEKYRFSCEGVIYIIHLL
EPHIKCSTRRSLALTTAQTVCIALRFFASGTFLYTIGDTENLVKSAVCRAIRKVYLALKQ
FLGVFVVFPSHLRPQVVKQNFFAIAGWCVTMFHITNVEAKWPGSVHDSRIFRESHLCTLF
ECGKGTHAEPNKTAFFHYTGDFDGILLGNRGYACRQYFMTPFPDPNPGPQTRYNAALART
RARIEMTFGQLKERFQCLKSLRVAPDRACDNVACAIVHNIA
>CC_copy9
MAALQRVLQLRVRERQRQRRPRSRLVTLNAFIRQHQTPLDILDDMAIIRRYRLPRGQIAQ
LLNSIGPQLMRATRRNFALSPEIQLLSALRFYATGSFLQVLGDGLGLCKASVSRAVQAVT
NALLPLAVEHIQFPASRQDITEIQQYFLTHHHIPQVIGVIDGTLIPILTPSVDGHV*ICR
KGYAAINCQVICDHKGLITDIVARWPGSTHDSFMFTNSSVGQEAQNLQGQWRLLGDSGYP
LRPYLFTPVANPMNNRETNFNEAHRVARSIVERTIGRWKMRFRAIHQSSGGLLFAPQKCC
AVIIVTAMLHNNAV
>CC_copy10
MADLALLEDVANGRIRSERIFRQQEDLLANEDEWLMSRFRLPRAVLLDLCGLLGPALQRS
TRRNHAVPVPLQVLTTLGFLATGTFQRELADRSGISQPTLSRVMPDVLKGIIASSHDSIK

FPYTAGEQANKKAQFAAMYGFPNVIGAIDCTHVDIRAPNVNENTFINRKHFHSINVQIIC
DADMLLTNVVARWPGSTHDSFILRHSSVGRRLEAGAIRDGWLLGDSGYPLKQWLLTPFLN
PHSAEERHYNMCHSRARAIVERTIGLFKGRWRCLDCTGGRLLYTPEKVCQIVLACAVLHN
VA
>CC_copy11
MALLALLEDYANGRIRRERVFRDHDDFLAHDDDWLISRFRFPRAVLLDLCAELGPVLERA
TRRNHAIPVQIQVLTTLGFLATGCFQRELADRSGISQPSLSAIISVVLNGILNMVSRYIR
FPYTVREQAEIKTQFAAMSGFPNVIGAIDCTHVAIRAPSENEFAYVNRKHVHSINVQIIC
DSNMTLTNIVARWPGSTHDSFILTHSSVGNRLNAGAVRDGWLLGDSGYPLRRWLLTPFLN
PQSAEETHYNEVHSRARAVVERAIGILKCRWRALDASGGRLLYHPAKVCKIVRACGVLHN
IA
>CC_copy12
VLLHFLSCHRRIWIRH*SGVWWKHVLTSWTDFEWCKNFRMKRTVLCDILRPHLERHITRF
RRPLSVEQQVAICIWRLATNLEFRSISHLFGIGLSTACMVTKHVTSVIVQELLQVYIKTP
SEDEFKVIIQGFRDHWGFQQCVGAIDGTHIGILCPPDSPADYYNRKGFYSVILQGVVDHR
MRFWDINVGWPGKVN*TRLFGNSSLYQRGHSGTLFPNSTERFQDVDVPIMLLGNAAYPLL
KWLMKPYPENSGATPAQAVFNHRLSKAWMTVERAFGRLKGRWRCLMKRYDCNIKNINTTI
TACCELHNFCE
>CC_copy13
FWLRPGRTSSWWDNFVNGVVVEDEWRENFRMSKASLVALSEELSPYIEGQTTNMRAPITT
LKRVALTLYYLSDEGWLRKTANAFGVSRQAVSVVVRQTCKAIAVHLGPKYIKLPFTESEA
KDLVLRFHRAHGMPQCLGAVDGTHIEIKQPSANSMDYINRKGKHSLNVQVVCDYKYRFMD
VVIKWPGSVHDAQVFANSKLNLYFKTGQIPPLKKQ
>CC_copy14
VNQLRQKEGEFSVLVLPLRDMDEEMHFRYFRMSAGRFDDLVRRVQPFIIHQSTHSMPVDI
RQCLAITLQILASGGSQQAVAASYKLSSSTVSSIVAEVC*ALWKALQPEFLPWPSVVQWE
NIAVDFWRLWNFPNCVGSVDGKHVNIKAPPHAGSDYYNYKGAHSIVLMATCDARYRFTMV
DMGGYGRECDGGVFKESRFGSRLLEHNLNLPPPANLPGTGVKIPHIIIGDAAFPLHNNLM
RPFPGM
>DL_copy1
MACPFLEDVLDEEALILRRAFRRERVFRDRSDPLAFGDDYLIERYRFSGDGLRYLCRLLG
PKIQHQTARSHALTVPQMVCVTLRWFASGSFLYSVGDAENLNKGTICRTIRRVCLALRSF
SNIFITFPGHRRPLYIKEEFYKIAGKLNLNVQPLVSNLISHSWILTIFCYSFMTLIGYNL
TFQMICDADCLVSNLEAKWPGSVHDSRVFRASPIYGRLSQGKPHILFVLACESFLLTPLA
DPQTPPQQAYNQAHNKTRARIEMTFGLLKSRFQCLHHLRVSPDRACDVIAACAVLHNIA
>DL_copy2
YTTEELYARFRFGNADIKYIADLVRPKLQRRT*RSHSLSVEEQVLIGLRFYASGTFYQVV
GDNIGVDKSTVSDVVKAVSISLASLVNQFVSLPRDDQIAQNKHKFFLLRNMPNTIGVTDC
THVHIQTPHERDWEYINRKGSCSINIQLVGDADLIITNCVVKWPGSVHDARILRESALYR
ELQTNRPDGIILGNSAYPLLPWLMTPFLAATTPAQARFNTAHCKTRCAIERLNGVLKRRF
ACLNYLRVEPQGASNIIVACTVLHNIPTNMPNT

>DL_copy3
MAALAVLEDLANGRIRRKRVFRDHHDFLAQDDDWLISRFRFPRAILLELCAELGQVLERE
TARSHALPVPLQVLTKLGFLATGSFQRELADRSGMSQSALSRTMPAVLDGIIHMLARYIR
FPYNAVNQANIKVQFAAIAGFPNVIDAVDCTHIAIKAPSEDEFAYVNRKHFHSINVQIIC
DAQMRLTNVVARWPGSTHDSFILSNSMVGIRLQASRVRDGWLIGGNLGYALVTWLMTPVA
NPQTDCERRYNDLHSRTRSVVERGIGQLKSRWRCLDRPGGMLLYRLENVCSIVLACAVLH
NVAH
>DL_copy4
WVQPRCHAWWQHVCDNWTERDWLLNFKMRRTTFHILCDSLRPRLTRQDTTYCQAVPMELR
VAVCLWRLATNLELRSISHLFGVGLSSACVFTQQVVAAINDTLKPEYLHTPSAAEFEQIV
EGFNDRCGFPQCAGAVDGTHIGILAPPHNPADYYNRKGFYSVLLQGVVDNNIKFWDVNIG
WPVRVHDAHVFTNSSLYEQGQNGTLMPRWQGTIQDVDVPLMILGDAAYPLLPWLMKAYAE
GRGVTPEQSALNYCLSQTRMTMERAFGRLKGRWR
>DL_copy5
QITHHFLRDRMRRRPKVWLYPRTSDWWENTASAFTDDQWLQHFRVSRETFSHLCMMLKPQ
LKRQDTTYRLCIPLQKRVALTLYKLAYPCDYKTVAELFAVGVASVCRCVHEFCTAVIEVL
KPQLVVTPNDSELVKIADFFLHSYGIPQCIGVLDTMQITVIKPPHYQSEPPNRESYLAIL
LQAVVDGNGMFWDLSTSCPTWVNDSCGLQESWACEGFTIRMLNVCGTDSGRFILGDEGYP
SQHWLLKPCTNTSWMTAEQEVFNSQISQAHSVAQHAFERLTGRWRCLNKRNDCSVEIVKD
MVETCCVLHNLCE
>DL_copy6
IWVQPRCHAWWQHVCDNWTERDWLLNFKMRRTTFHILCDSLRPRLTRQDTTYCQAVPMEL
RVAVCLWRLATNLELRSISHLFGVGLSSACVFTQQVVAAINDTLKPEYLHTPSAAEFEQI
VEGFNDRCGFPQCAGAVDGTHIGILAPPHNPADYYNRKGFYSVLLQGVVDNNIKFWDVNI
GWPVRVHDAHVFTNSSLYEQGQNGTLMPRWQGTIQDVDVPLMILGDAAYPLLPWLMKAYA
EGRGVTPEQSALNYCLSQTRMTMERAFGRLKGRWR
>DL_copy7
PGRSSSWWDNFVTGVAVDNEWRENFRMSRASVVALTEELRPYIEVHRTNMRESIQPLKRV
AITLYYLGDEDGRLRTAADAFGVSRQVVSVIVRHTCKAIALHLGPTYVQLPLTEPGAEEL
VAGFHRAHGLPQCLGAVHGTHIDIKQPSTDSTDYTDRRGRPSLNVQAVCDYQHRFVDVVI
KWPGSVPDAQVFVNSKVNTYLKTGKIPALKKR
>KM_copy1
PGRTSKWWDNFVNDVADAEEWTENFRMSKVSLHALSEQLRPYIEGETTNMRSPVDVLTKV
ACTMYYLSNEGRLEKAANAFGLSRSTVSIIIRQTCRAVSVHLGPRYVQLPFTEPEARALV
TGFEDAHGMPHCLGAVDGTHIEIKKPSVNTLDYVNKKKHSLNVQAVCDYRHRFMDVVIK
WPGSVHDARVFANSDINSSLQSGKIPALERRLLEDEEPIPVFLAGDPAYPLLPYLMNEHP
DGGATPQERRFGLRMRRARMVMEQALGRLKARFACLRRPMDINLKDLPHVIYACFVLHNY
CE
>KM_copy2
MADSA*LEDIANGRIRRERVFRDHTDLLAHDVEWLISRFRLPRAILLELCLELAPELQRD
TRRNCALSVLVQVLSTVGLLATGTFQKELADRSGISQSSLSRAMLAEWDALIHMLPRYMK

FSYTLAEQANINLQFAARSHFPNVIGAIDCTHIAIRAPSGNEFAFVNQKHFNSINVQLIC
DAQMCLTNIVACWPGSIHDSFILQNSRVGNKLQAGAVRDGWLLGNF*KSELLKAFLV
>KM_copy3
RRTWVNPRSHDWWQLIQTTWTDRDWLKNLTMRKCTFQNLCGVLQPYLTWQHTRYRRPHSV
EICVAICLWRLATNLEFRSISHLFGVGILTCCLITQEVITAINTVLKPHYLKRPCAADCR
KIVQGFKD*WRFPQVVGVIDGTHVPIKAPTNTPADYFNRKGQYSVILRAVVDHKMRIWDV
NIGQPGRVHDA*VFALSSLFDQIQSDKLFPHWNETIEGVNAPLLILGDAAYPLLPRLMKP
FRESIAMTADQRNFNYRLSQGRMTVERAFGCLKGRWRCLLKQNESHISLISQVILECCVL
HNF
>KM_copy4
VWAFNQASEWWDVVVPGFTDAQWLQNFRMSEKKFIYLCNKLRPALKLQHMTFRHCVPLKK
RVAIALWKLATGSEYRSVGHLFGGSITMVCRCVQEFCVAAETVLVPEQIRFPDEDHFREI
AMYIENRWGLPQCVGAIDDCHIPIIAPQHYHTDNFNRKGWHSIILQAVVDGKGIFWNLFA
GLSGNSHDARVFRLSTLSELASCGNLFPPHIRNIGTVATGHYILGDSAYPLLDWLLKPFP
DTGRLTSEQQFFNKKMCSARVVAENAFGRLKGRWRCLLKRNDCNVGLVMSMVLTCCALHN
LCE
>KM_copy5
LVNQPSVWAFMRSSEWWDVTVPAFTDSQWVHNFRMSRETFHYLCNTMHPALHLEDTPFRM
SVPLQKRVAIALWKLATGSEYRSIAHLFGVSVTTVCKCVQDFCSAAVSLLVPEQMRFPDE
RKFREMAELFENKWGFPNCVGAIDGSHIPIIAPHEYHTDYYNRKGWHPVILQGAVDGKGQ
FWNIFAGLPGSLHDARVLRLSTLWELVGQGRLLPAEKQIIGAADIGVYILGDAAYPLQDW
LMKPFSDTGRLEAEKQTFNCKLSRAHVVVENAFGRLKGRWRCLLKRNDCDVNLVKLMVLT
CCALHNLCE
>KM_copy6
KLWVRPGRTSSWWDNFVSGAVEDGEWSENFRMSRASLVALTEQLRPHIEGQTTNMRAPID
PLKRVALTLYYLSDDEGGLRKTANAFGVSRQAVSVILRQTCKAVATHLGPKYIKLPFTEP
ETEDLVSGFYSAHGMPQCLGAVDSTHIEIKQPSINSADYINSKGRYSVNVQAVCDYRYRF
MDVVIKWPGSVHDARVFANSRVNLCFKSGKIPDLKKQ
>MA_copy1
MACPFIEDPVDEDAVLIRRELHLPRERILRPRVDFLSYPHVFLFERYRFSRASITYIHTL
IHPYIRHITHRGRALTSEQMLCVALRFVANGSFLYNIGDAEHISKATVCRIVRKVCLTLK
RFLLFFVVFPGHKPLRAIKEEFHRIAGPLQDFQMWLDALMEHIFPSMHLLKMKGTMSTGG
PSTVLMCRYTDYVSFSALPRSYVMLHILSRTWRPSGPALFMTHEYIVSQFLIGEIDGFLL
GDRGYPCQPTLMTPYPEPAAGPQQRFNVAHCRTRARVEMTIGLLKARFHCLRHLRVTPER
ACDIIVACVVLHNIAI
>MA_copy2
MACPFIEDPVDEDAVVIRRELHLRRERILRPRVDFLSYPHVFLFEQYHFSGESITYINNL
IHPYIRHITHRGRALTSEQMLCVALRFVANGSFLYNIGDAEHISKATVRRIVRKVCLTLK
RFL*IFVVFPGHKPLRAIKEEFHRIAGQDFQMWLDALMEHIFPSMHLLKMKGTMSTGGPS
TVLMCRYTDYSVSFFAPPRSYVMLHILSRTWRPSGPALFMTHEYIVSQP*VTGCNVVSTC
MPYTLISMHASDVNFLIGEIDGFLLGDRGYPCQPTLMTPYPEPAAGPQQRFNVAHCRTRA

RVEMTIGLLKARFHCLRHLRVTPERACDIIV
>MA_copy3
RQRVYVERTKPLEQYTTEELYVRFRFGKADIEYLVNLLRPKLQHRTQRSHGLSVEEQILI
ALRFYACGTFYQVVGDYMGVVKSAVCDVVRDVSITLASLANEFVSFPKDNQIAQAKRSFF
LLGNMPNTIGAIDCTHVHIQAPRENEWEFINRKGRHSINVQLVCDADLIITNCVVKWPGS
VHDARILRGSALYRDLQTNRPDGIILGDSAYPLLPWLMTPFLTANTPAQARFNTAHCRAR
CAIERLNGVLKRRFACLNYLRAEPQKACNITLACIVLHNIA
>MA_copy4
AATSVQRNVWVRSRSQDWWDSV-RGFTDTEFINNFRVRRETFEWTCQRLSVSLAWQDTNL
RRPISVSKRVGVRLYWLATGSCYRTISNLSRIAKSTVCLIVQEFCKAVRHVLMPEYIKLP
QGGHLQEVIEGFRQRWGFPQCAGAIDGTHIPIIAPEDNHADYFNRKGWHSVVLQGVIDHQ
FWFTNIYTGWPGSVHDARVLRNSKINTMAERGELFPKEIEEIMGVQVPIMLLGDPVYPLR
NWLLKGYSDTGTLTDAQRYFNMHHSQARMTVDCAFSRLEMRWRCLGKCLDVDISAVPTII
SACCILHNVCE
>LCt_copy1
MACPFDHDPVDEGAAILRRELHICRQRVLRPCIDISALPDNFLFERYRFTSQSIIYIHNL
IHPHISNITSRSHALTSQQILCVALRFFANGSFLYNIGDAEHLSKATVCRAIRRVCLALK
RLLPIFVVFPGHKPVRAIKEEFHRIAGEGCRDLLNSFNIRLYYDIVLPPQTILCTVKIIC
DAAHIISNVEAKWPGSVHDSRIYRESNLSARLQHGEKPEDLQ
>LCt_copy2
NPLNQYDEITSRDRFRM*KEDALEIITLLEPRLSSMSQRGRPVPSSLQVLITLRFLASGT
FHRETSDLCGVSEATVCRIVHNVCSAICELRNLYIKFPDAAEQANYKVHFYEYGNFPGVI
VCIDDGCYVEIKCPSTPDAEEYRNHKNWFSINVQAVCTPNLEFSNIVARWKGATHDSRIF
HNSPLCAQFERGQHSGLLLGDSGYSQSSYLFTPWPHPTTTEQQRYNRAHIRTIGMVERMF
GVWKNRFQSLHNTLRFEPRRCCKVIIATAVLHN
>LCt_copy3
LLEDLANGAIR-V*VFKERKDLLANHDNWLISRFRLPRPVLLELCAELWPALERNAARSQ
WLSVSTQVLTTLGFLATGAFQRELADRSGVCQSTQSRVMPAVWDGIIRMSSRYIRFPYNA
VEQANIKAQFAARASFHNVIGAIDCTHIAIKQPSQDEFVYVNRKHFHSINVQIICDAQMQ
LTNITSRWPGSTHDSFIMTNSLVGNRLEAGTMRDGWLLASFNCPV
>LCt_copy4
RRFWVRPGRTAVWWDNFENEVVVPEEWRENFRMSRSSLLVLSDLLRPYIEGEMTVMRSPV
GVIKKVACTLYYLSDEGRLRKTANAFGLARSTVSKIVREVCEAIAVHLGPMYIKLPTSEE
SVQQLIEKFEEAHSLPQCIGAVDGTHVEIKQPPVNSTDYINRKGKYSLNIQATCDYKYCF
IDVVVKWPGSVHDARIFANSALNDYLKAGKIPPCPKQIVEDEEAIPVYLIGDPAYPLLPY
LMKEYVNGGSNVQEQYFGLSLCRARMVIECAFGRLKGRFGALKRAMDINLDDLPFVIYAC
FVLHNYCE
>LCt_copy5
RRFWVRPGRTAVWWDNFENEVVVPEEWRENFRMSRSSLLVLSDLLRPYIEGEMTVMRSPV
GVIKKVACTLYYLSDEGRLRKTANAFGLARSTVSKIVREVCEAIAVHLGPMYIKLPTSEE
SVQQLIEKFEEAHSLPQCIGAVDGTHVEIKQPPVNSTDYINRKGKYSLNIQATCDYKYCF

IDVVVKWPGSVHDARIFANSALNDYLKAGKIPPCPKQ

Supplementary data S2. Predicted fish *Harbinger* Myb-like proteins.

>Harbinger-3_DR

MAKANKKRNFTECELEVLLSEVDRRKTVLFASLSSGINNKRKKIEWESLADAVNAVGSER
RTVSELKKKWSDVKVQVKRRTAAHRQSVDRTGGGTGDTALTPFEERVASIVGDTLLSGVV
SVSVGDTDVLEEAHEDGAGTSTDTDFVPPEEPEPSVSGATPRVSSASASAEARPSGRVLT
QAVLESQQDIVRAIGDINNHLKNISNALTDISQSLKELVKK

>Harbinger-2_GM

MDKARKKNFSECEVEVLISEVEARNKILFGSLSSGISTKTKKLAWESVAKSVNEVGAES
RTVADIKKKWSDIKVDVKKKVSAHRRSVGQTGSGAG

>Harbinger-3_LC

MEPRKATQKSCLPRRQRQQQQQQDPQREQQQRDPQQEQLLLLQQQVELQQQQQLGGPQGT
AGSTAPGSRKKRFLRQEVEKIVAGVDRAAAVLTGTAATSMAQKEAEWQRITNEVNALGHC
RRRVVEIKHKWRDLRVAVKKKMASINRATGGTGGGPPPQKLTPLEDIVSKHISMMEVRGA
EESYDTSESKYHATEEGTQPSVREMAMSPSRPVAPSSSVPGHRPPSGSQEEPQPLDDPAP
RASTSQDAATTSTLLRQAGKGSGLQTAVEGDAEGQHPEAGLSSQSLDEASIHSASLDTLR
EVSPPAPPRHRGSQAARRGRGRHGGRRGYALRDFYELEWRGPPVQRAILELATERARLDA
SEARERRRHERLIARANEGHAEALSDLGQRVQHIANLIERVDANMVEGQRQMLDALERLA
DGQHRAATALADITEALRQQGPRERHSVGVGTAASPPHDLQASPRMSPGSVSSETARTSG
TTVAAGAPSTPEAPAATDTQPGPSGGPQLQQTTPVVTAVRPPTRGRGRGRPRGRPPRSRT
Q

>Harbinger-3_GM

LIVSVSAFPELFNSTLAGYKDRTVKTKAWLKVSEEVGLTGKSICLSEEECRRKWKVLRDT
YLR

>Harbinger-1_LC

MRGCRWGGLLTRRAIRLEGKHHLKGPHQEIHSNDMEPRKATQKSSRTHSQGEREQQQQQE
AGEPQPDKGTTGPRTRKRRFSFEEVERIVEGVDRAANVLLGTGTTSLAQKEQQWQRITNQ
VNALGYCRRGVTEVKHKWRDLRVTVKKKMANIARAKRATGGGPPPAGLTPLEEVISKHIS
AVEVQGIPEGQDTIDRKHHGEQHAVTHKRKEHAQHARVSTLARRHLSRQQQISLYVGFTV
FFAISHCSGLHSDLLRGSPRGILLRAGAPVTLRSRRGNTVTPRRCSQGIQPPGIRCQRHP
SAPGWNRLTHVPFSLSLSTGPQPQQRRSSPTVPSTSTMATTGRRLQDAGANTPGAPATTT
TTSSSSTLIPIPVPCAPQQSQPRTPLPPTAPCTTVPPGRTHIECPSSDVCTATAHSQSSV
GTPTPPDDLPSTSHPLSTSPGGPTTPPELLSDSSDDILPPAQGTPPHSSQWAGSRLQAAV
EGDYDEQHLDAALSSSASEDALVAPATGEDTLRGSMPPAQRVPTEGESAHRAHRRRGGRR
PTTLRDFYTLERHGVAVQSAIVELATQRAAFDAGEAEERRRHERRVDEALERMTASVDGL
RQSVLHMSGLLEGVADSQRQIVEYLRQIAEGQRQDPRPVAPAVRDLRQRSRDVGQHPGEG
TSAPYPHRPPSSPAMPADPVRRGRGRGRGRRRPPRSR

>Harbinger-4_LC

DTGAPGPRKXKRRFEVXEVDTIVHGVDRAADIVLGAGSSSLAHKEEQWQRITHQVNALGH
CRRGVAEVKHKWRDLRVSVKRKMANIararratgggpppapltplEEVVARHISTLEVEG

IPGGHDTIDRK*QIAVEGAQTTGTWGSTGMQQQ*GHAAQRYNTTSTHQVHTLYSQRAXKH
HGEQHAGTHKRKQQSQHAQGSTpppppppYVPATRHLS*VPLSLSLYTGPQPQQRCRTRS
DHATSASATPRRRLQDAGTNTPGAPGAAAAATSRSSSTSTSTTTXTXTCTCTXSTTPTVN
PIPVPCAPPQPEPLTPFPPTAPVTPASPGRTHIECPPSDVGTTTAQGESSVGTQPPP*LP
QAQGSPTHSSDRMDSTVQAAVEGKYxeqlleaalsssaseDAPVAPATGEDTLRGTIPPA
HRVPTXGESaagahrrrggrrPTILRDFYSLERQGVAVQSAIMDLATQRASFDAGEAQQR
RRHERRVDVALQRVTAAVHGLTQSAHHVSGILEGLVDTQRQVADTLRQVADTQHQMVDLL
HQLVDGQRQGAQPHAXAGRDLRQRSRDVGQQVGAGTSARYPQRQRSSPAXPSDPM
>Harbinger-2_DR
MAETKKQRKVIFQKEEINIILEEVELQKHIIFSRFKGSHTNKEKQKMWDDIATKLTATRG
IKRSGNEVRKKWQDFSSLAKRKRALQRTTINKTGGGPNDAPILTAEEEKALSILGTTASD
GICGGIDLHGGEGLRQPEPESGPPCSQEQSGSPPIDRQLPSPSPPNSPTDQPPSSIVQAT
ATTPRFENICGCSQDLVQLEREKLDVLKDIRQSLKEANEMNYNFQREIIELKKAKMALEE
RRLSLEEMSFARPSISVPIILPDESDPGEQTTNLNQ
>Harbinger-1_TF
MEGKTEGGCFSATETNALLEGVRENYHVLFGNFSSPGQSGSNAKTKNEIWEKITELVNAT
GGGQRRSVDQVKLRWKKLKQKATKDHSEAKTTPTGNKPAKRGEFTDVILDIIGGADSQAL
HGIVPCDVGESIGSQGPLNLSSDGTSTLTFESDQPELSTSQTLTQAADVVRVTPSPQPPP
LTCRSKRPKSLTQDHAYLKLLKVETERAQQQLVLTDEQINLTILKQEQVKLQIEKQLM
>Harbinger-1_TR
MEGKTEGGHFSATETNALLEGVRENYHVLFGNFSSPGQSGSNAKTKK*NEIWEKITELVN
ATGGGQRRSVDQVKLRWKNLKQKATKDHSEAKTTPTGNKPAKRGEFTDVVLDIIGGADSQ
ALHGIVPCDVGESIGSQGPLNLSSDGTSTLTFVESDQPELSTSQTLTQAADVVRVTP*SP
QPPPLTCRSKRPKSHTQDHAYLKLLKVETERAQQQLVLTDEQINLTKLKQEQVKLQIKLL
EKQL
>Harbinger-1_GA
MATAPDKKRSSYFSPAELDVLMQAYGEYEHIFKKKSNTAAAAKDRELAWEKIAARVNACN
PAGIKRTWQQLKMKYKNIIQSANRKKAEARKTGGGPAPPPLTNAEEMALSLNAGRPMAEG
IPGGTSSEPVTPEDLSAFIKYSDGKISLLEPPVPTEPQAVDDGDEETVSAATEMPTESVE
EQQEDGPSTSGAQMNTVQ
>Harbinger-1_OK
MATRAAYFSPSEAQILMEAYEEVKDIIKKKGNTATVIKQREKAWQSIADRLNANMNGPKR
TWQQVKIKYKNILQNGMVPD*YLTKHKHILYPEGACSHIVCTVLAVKKNTHRQGTGGGSP
KADLTPAEDMALELNKGRPVLEGIPGGKETSIGSSQDATRFIQGMSFHLYMGYNHIHIES
IWTV
>Harbinger-1_CC
MATRAAYFSPSEAQILMEAYEEVKDIIKKKGNTATVIKQREKAWQSIADRLNANMNGPKR
TWQQVKIKYKNILQNGMVPD*YLTKHKHILYPEGACSHIVCTVLAVKKNTHRQGTGGGSP
KADLTPAEDMALELNKGRPVLEGIPGGKETSIGSSQDATRFIQGMSFHLYMGYNHIHIES
IWTV
>Harbinger-2_CC

KMTRKPQFSKHEIAVLLEEVENNKVQLFSKLKKYCITNTDKNKIWAEITQKINDAGYGYE
QSPEEVRNKWRDFARVTKRRAAACKREANKTDGGIDSVPPLPTEEEKVLAILGPVALEG
>Harbinger-1_SS
MATGEKRARTSYFSPVELEILMNAYAEYEYIFKKKSNTVAAAKERELAWQKIADRVNACN
PTGTKRTWQQLKMKYKNILQTGKPIEKRLRAEKQVGVHHHHSEAEEMALSQNSGRPIAEG
ISGGSSSDPTTPQDTRAYIRVTDGVICLVEPSAITDLHAVEDDEDTLSAATEREDTERPI
ESNAGNYNEEEGPSTSTAQLTSLPVKQLYKVYLEKQIEKCNLEIDHIRLQMQKIKIEIQL
LEHQLKVGEVPTGG
>Harbinger-3_OL
MADAASETGQWKGSEVTDLISIWGDRSIQAKLEGSYRNRAVFEEIAREINERGYRRSWLQ
CQRKIKSLRAKYKEVKDWNKQSGRQRITCPFYEELDRILGDKPSVQPVELLDSCFAQEEP
EEEGPGPAAVAASLAGRSCGDTGDAFLATPSPSTSDGSSASTSEELASSSASSVNSNGSS
TSTASTSARSRKRKSKMEATLDAFANKITTALKEDDTDILLKMQAAQHEHEKKMFAMMTR
FMERSFPLPQSHQAPMNSSHLYSPIPSPTPFTRFPVASSPSPTPRRPFFPDLNRLPFNNT
FQHPHINQPFSQAPGHTQAFDEDPLQPSQCKDDETHYTSF
>Harbinger-7_DR
IMEESSEISQWRDSEVIDFISIWGDSSIQGKLEGSYRNRSVFENIENVTNARQMLERGHR
RTWLQCQRKVKSLKGKFKEAKDSN*QSGRVTCPFYNELERILGDKPSCQPLELLDSSFT
>Harbinger-1_ON
MSYPFRLRSHCRRKRIKSDFFDPMRPISDHESDCCVNGQVALNPSFTSLTRDRRQLSVPE
KTNQKSMAAETGDGASQWTKTEVAELISIWGDNSIQAKLEGSYRNRAVFECIAKEMIERG
HRRTWLQCQRKIKSLRAKFKEAKDINKRSGRGRVTCPFYDELDGIGGDKPSCQPIELIDS
SIETAEEEPESPGASASPAPSLNIEKGCMIVFSKGACTLGEDTPVSLTFDSASDESSSSA
VSADNSIKKNAQTRNTSAKSRKRKSKMESTLEIFADKITSALKNDESELLLKMQAAQHEH
DLKVLSMLTQLITRQQPPFYQQAVQVPHLYCPTQSSLIHSTPYPMASTQSPPFEMSNQSL
SFQQDLAQPLPFGNSLPPSHNLGQPFRPERPNPRDQAQHASFNQAFTSSYPPPDEQD
>Harbinger-1_DL
IVGISACSRHTEIDLAIAHRITDGNMDKSRASYFTAAEQQLLMEFYEEVEDIRKKGNTNA
IIKEREKAWQTIADRLNA*VVRTNLSGHKRTWQQVKVKYKNILQAGX*L
>Harbinger-1_LCr
LNMETDRKRAAFFTPIELEILMTAYGEYEHVFRKKCNTAAAAKERERAWESIAARVNA
FISCRCNPAGEKRTWQQLKMKYKNIVQTGQTSA
>Harbinger-1_MA
MATGPSKNRSAFFSPTEMDVLLQAYSDYEPIFRKKKNNNTAAAAKEKELAWGKIAARVNA
YRFSRESITYINNLIHPYI-RHITHRGRALTSEQMLCVALRFVANGSFLYNIGDAEHISK
ATVCRIVRKVCSGFPNAVGCIDGTHIPINAPT
>Harbinger-5_DR
MDNRQYWSVEDTRALLNIWAEENVQRQIDGVCRNEDVIKYIVAELAKAQIQRTTVQVREK
LKKLRAQYKAIKIHNGQSGAHRKNFPWFEIMDGVLGHRPSVSGETTRDSMAANLTVGKSL
YLLCKLHKSKPMCIKKYPVFEAYFVHFFFSTESRKRKATSLSRDFMQYIREQNQEQREAE
QKLRREDREAEMLMRREELAAYMELQQKEMDRRQKDSDVLNSILGSLAAALLPKKFIINK

SSFLSVVVYCVCCVLGIYKFASCFIVNHKNMWRSMNKVYCSYDIACFILMNKSKFLFGVS
KQVLLNKNMLANCCLVMVVVCNDSNALGNQTVIVIDKYAIQICWMCILSLLSTKIQMLRQ
LWGHVFGMGVRCWCRQTPKQQKLQMKHIPSAISLILDSWGAMVSCFDFYSFSGYEFLIST
IKLLLSTIQLLIPKILLFISR
>Harbinger-2_NF
WSNEEVKIFLSLLADXRIQRELDGSVRNEKIFQGLAXAMSTHGXERTSKQCREKIKKLKA
EYRSVKDHNSRSGADRRHWKWFAEMDAIYGERPVSNGRESGVDTATPAXTG
>Harbinger-2_BF
MAGRKEGARRCIWSTPETRCLISIWGQAEIQKKLEGPRTKMVYAKIHQLMKNAGYEDRTA
EQIKRKIADLKAAFNNAVKKNKKSGEGRKLCHFYNELNVFLGGREALNPQSRLESIADPE
SSDSEGGEDGDGGEDGDAQDQALDSPRGPSPPPEDVTEDSINVSVDSSSSEKRKADEEGG
GVEKKGKTASQQKKGKPGFVKKAPQNKTRIENSFSALEKSFSSLQDEQEKRQREAEKQKM
EEWKEHDLQVVRMQQEHETRTVNNMMGQFMTMMGQFMTVMQQGSTQFPPTYGGQSRSPSP
MPYNPSPYPPIAGGQSWSSSMPRSAVPSGSQYASMHGGQSQGPSMSNPALSMPHTGSEDP
STSEETYFQL
>Harbinger-2_LCr
IGPGPEIHLFVRSDNAMNKGKKMNFTESKLKILVNEVEILFGSLSTGINMTKKRREWERV
CEAVNAVGSEQRTHRCGQVWRCG
>Harbinger-3_BF
MAEGNAGRSRGSRCTWNYEETAFLISVWSEEEMLRQLETPRNKFAYVKIVDRLAEEGIIR
TFDQVKTKIRDLKHDFKKTWQHNHKSGNGRLDPPHYQALKEFLGCREALEPSSLFESTEI
TGVLCLEVRAAQSLLPLSSIDLDTSTVNASNESSNSNQLDMSTTNASNESSNSDQLDTST
TSASSSGQLDTSTSARSSGQLDTSTSASSSGQTGGKRKAQEDGDNNPKSARQEDKNGKKG
FSKKERKRKTRLETSFSELEKTIHNVHKDQDTKLMEAENKRAEDWKAHDLRVMKMQQDHE
KETMSNMMGQFVAMMGQFMTAFAPGQQGNSSASNQNPPTAAFQGPPTSDFQRPSAANFQH
PQSSASGFQRPHSPAPGFRLPGSPYIRLSAPLCC
>Harbinger-1_KM
MFLRXFPSCIPICVELRVHETPPLTRLHYK-ICKYVPHVRFSSLRDRKLCLDVFLAFARVR
GVTVXVKLQLSEQRTPAEEQKVKWSDIKVGVKRRXAAHRLSSNNVRHHLLYMSYTLLL
>Harbinger-4_GM
MDPNQYNYQCYYSYLQNQSLPTSENSQNPPQYIMYRPPHSSENFQNPPQYVMCPPPPPTS
ENSQNPPQYIMYPPPPHMWYMHPSSNVEPPTSGSSQNPQIYSQPSTPTNSNTCYRPSLSN
IVLPSNEIESPVGVDGIQLDEGDENSSGKKSRTAFSVTEDVILVRSWLNVSKDSIIGVDQ
TSKQYWARIKNAYNNDDVRQSGQFCERSWTQLKSRWNRIHPPVQKFNGCYKQADKHRRSG
SSEKDVLADAHMIYSQDTGKKFEVEHAWLLLKDQPKFDAEFMSKCSKRTKVSTSGNYSSS
SNPETPIEVEEYDTPSPMSRPIGQKAAKRKSKGKECPNTLDLSGIESVMKDKNMNTSKLI
QLKEAQEKRLQEQERRMEYEILMKDTSNMSEQQRKDHEKYCDHIRKKLGY
>Harbinger-4_BF
MSDPAPDNTPVLSDPSGSDDAQAVSAPKKTRAKKNSFSLDGQTEMALVEWIRENEILWNR
KLISYKRTDLKDALWNDKAKELDKTGICLRGWWRSIKDIFTRLHKKKSGDAARKLTERET
WIFTSCDFLRPLVRHVGQPLRSVSMNITAIFLRLRLLHSCLFQTCHFVLFSY

>Harbinger-1_BF
MPKGRKAAGKDKGKQPRRPYTRRKKVLKTSEELDAETSGSELSEPGGGRGESNVDGSSDE
PLSSSQQPSQKASITEDQDEKIASFIESHQAFYDMSTPEYKNKQKKDAWLKDVAPEIGLT
PKQILTRFQTMRTDYFKLKRKLAGKSGQGQTRVTPLQDFKLRRYKFLDAHYRGRASSTEL
GSVKQPVFSVDEEEEEEESSDGPRHDVSKASILPKNKKSKKGLGDVLVELLADSQKELKQS
QATLAESSSSASSSTRGGSERDAFAQWLARVQHDIPDDRWRSYQREVFDVALRYSTGTQP
PQTRQHEPLDPPASP
>Harbinger-1_DR
MPSVPCPGPFPGSFEKCECAESGPSTVHLAGPGPVGRGVPERGSLGLWRGTLVVRVQACQ
SPKLKTLRHLLRHFKTGKNHVTSLSATLLTPKSNRKRTSDAFSATIPKTTVAWKGSLWST
DEVQCLLDVWADESIQEQLDKTHKNSEIFRKIKDHLLGRGYQRTTEQCRDKMKKLRAQYL
KVRDALRRSGSKPEEKDKFKWYDAVDNIIGSKPSSQPNVLESHSQSMGDSTPSTPSETED
TGSERSQRGE
>Harbinger-6_DR
EQNCLLNKKHPNYIKRGLKDALWEHKATEMGKSSSQLKKWYSNMRTRFGKIKLTPSGSGN
TELTARDHWILRHFEFLRPY
>Harbinger-8_DR
MHFRCSVAHVSGLVISTTIFKDLNCRKHKHGSALYLYDSSSADYKDTQMAANSWKEIACN
VGLEVTECLRRWKNTRDKCVQLRKKMATKSGDPGGTKFPAFYHFLTSWRNKIPSFLSLSN
VTVVNAGRSCSKNWPKNMIKAASLEAQLLTCCVEFRMSTSLRPCLMSIKHCLSGRKRNEH
TLIYTLNYFCHFFVNSLKCLFASNFFFHFSPSYISVNFFKSCMCDFVLHNKQQYCQFYNG
YLFTWSFIHLIHIAYEYNYSVCWPPKSQKHFQSTVWLWSDGNCKSASPRADAVL
>Harbinger-2_SS
MEERIIFAVSSKPCLFDTTADSYRDINTKNAAWRQVSTIVGLPEEDCRRKWRGLRDRYQR
ERRAEKEKKRSGSAASGQQPWRFCHILSFLDPFIVPRATSGNFTARPSTTSSTSVVATGA
SRPSTSSTSATIASTGAARPSTTSTSSTSMTTTGAAMEDDEMEEAPLDLGPPEIIMNLTE
VTTEDLVEQDVAVETNRERNEEEQRHTRRHRRYQPPDPLNSFQQRLLQAVERDAAQPDAH
RKEDEETLFAMSLVPTLKRLPQQRKAAVKVKMYQLLFEAEFNGTFFFSPHHRDGVNLADQ
LTGRTGRGEELSGCCFDLPHCTFLFTHISSALPSLSLWPCLKFPYYV
>Harbinger-2_LC
FALQEVETLVHGVVRDIEVLLGASASIPAKKRSKWKCITDEVNSLGHCYHDIEDIKHKWS
DLRSTVKKKIVKL
>Harbinger-1_OL
GACFRKEVRPTLRLNLNSELVNREIDNSEFSVSEXLIRVRSINSEWTDSELSVRTATIRS
HYQWSADITSHHGNREKKRSAYFSPAELDVLLQSYSEYEHIFQKKSNTAASAKQRQLAWK
T*LLKLMRKFAFKSLL*NIGCNK*LFK*RKE*NKKWRYLGVLLKCYSWCKCMKCCIVYRT
NLGGENAFNVGNV*RTFLLTFPSCKRWSV*Y*YMQLCF*K*TFVYC*TFRCAKTSPFFSL
VFPTDRGQKRRGDFLQGRREFSFGVHFPLETLNLQSGLRMTPKQLFWE*HLLFI*LSAPS
SEHTIYTTHTPPLRAHPPPPHLHLHSPLPLSLVDRTPLSTHTLQHYPCIDQVV*VYMGLR
K*AVLANCVSKK*LLH*SNSMSLISFSCRCNPAGIKRTWQQLKMKYKNIIQSGQI
>Harbinger-4_DR

MNAMAKKAREVFLWTDDEVELLLNVTNEYKTAMVIENVDWESCQTKYNDILNRFLENYPT
SEEAMATGKDFPHKKEDITKGTLFIHSFFVLAYKYYKSRQTYVHLNGKLLVMQQNICCVV
GMEKGARPRVFVCISMCLFGVCFYLFNACYVIFCIYLNVYLFIICPYSIGLCFVLIGHVT
TKLKSIRLRYRQAVDCGRKSGHGRVVLLFFELCESVWGGSPATVALSEGIETSCIGSEVS
EQSSTGMSISAGESVTEGSPSAVSGHRRQRLDATLSGHRIERLKAQALINAGAAYCRFGK
PWSHASSTTTPTISKPVPIRPHAHKKTTRGVQSTSSTYNPTQHLAIWRLHKFVVFLVLFQ
SVHTLMQTYMHSCTCLLFIATVYCTNLHIVHTYAQTNNQLAHISLYIYIYIYIYIYIYIY
IYIYIYIYTHTHIHFCKLLCKLISALLFQNVISIMLYFLRISFKMYTFL
>Harbinger-1_NF
FRMSANRFDDLLHRIRPHINHAATHSSPVKLEERLAVTLRVLASGNSQQSVAQSYRLGST
TVSLIVTEVCEALWSALCSDFVSLPDKNKWADISQEFWRVWNF-PNCLGCVDGKHVIIKA
PPNAGSDF*FNYKGTHSLVLMAVCDANYRFSMVDVGAYGRESDGGVFQECM
>Harbinger-2_OL
ETEVLVFLFWLACGTSYGVVARAFDMPRSTVSDVVHRTADRILQLMSRVTQLPSGNELPL
VASGFXQLAWSSAFQKVAGSIDGCHIRIKALKENAACYFNRKLFYSIQMQAVCDSNAKFI
DIFVGNPGSVHDSRVLRKSPIYVNQTLPT*RILLGDGGYPCIAHPITLLTPYREPMRNAV
EARYNQHHXRTRSVVERAFGMIKMRRRAIFLGALEVKPTFAMKIIACSSILHNLCISEGD
CLEP

Supplementary data S3. Genome coverage script.

```
#!/usr/bin/python
# -*- coding: utf-8 -*-

import sys
# arg 1 : Repeat Masker file .out
# arg 2 : output file
# arg 3 : genome length in base pair

file = open(sys.argv[1],'r')
rmk = file.readlines()
file.close()
SIZE = float(sys.argv[3])

##########
## Main ##
##########

COV = {}

for i in range(3,len(rmk)) :
    li = rmk[i].strip().split()

    ## Family ##
    fam = li[10]

    if fam not in COV :
    COV[fam] = [0,[],{}]
    if li[14] not in COV[fam][2] :
    COV[fam][2][li[14]] = [0,0,0,0]

    COV[fam][0] += int(li[6]) - int(li[5]) + 1
    ## Number of the element ##
    if li[14] not in COV[fam][1] :
    COV[fam][1].append(li[14])


    COV[fam][2][li[14]][2]    +=    float(li[1])*(int(li[6])    -
int(li[5]) + 1)
    COV[fam][2][li[14]][3] += int(li[6]) - int(li[5]) + 1
```

```
    if li[8] == '+' :
    COV[fam][2][li[14]][0] += int(li[12]) - int(li[11]) + 1
    COV[fam][2][li[14]][1]   =   int(li[12])   +   int(li[13].re-
place('(','').replace(')',''))

    else :
    COV[fam][2][li[14]][0] += int(li[12]) - int(li[13]) + 1
    COV[fam][2][li[14]][1]   =   int(li[12])   +   int(li[11].re-
place('(','').replace(')',''))



## Output ##

file = open(sys.argv[2],'w')

file.write('Class/Family\tTotal_coverage(bp)\t%_coverage\tNum-
ber_of_elements\t%_length_>30%\t%_length_>50%\t%_length_>80%\tTo-
tal_coverage(bp)_80-80\t%_coverage_80-80\tNumber_of_elements_80-
80\n')

TOT_cov = 0
TOT_perc = 0
TOT_el = 0

for c in sorted(COV) :
    TOT_cov += COV[c][0]
    TOT_perc += float(COV[c][0])*100/SIZE
    TOT_el += len(COV[c][1])
    c30 = 0
    c50 = 0
    c80 = 0
    N80 = 0
    L80 = 0

    for p in COV[c][2] :
        over = float(COV[c][2][p][0])*100/float(COV[c][2][p][1])
        if over >= 30 :
            c30 += 1
        if over >= 50 :
            c50 += 1
        if over >= 80 :
            c80 += 1
```

```
        if        COV[c][2][p][3]        >=        80        and
float(COV[c][2][p][2])/float(COV[c][2][p][3]) <= 20 :
            N80 += 1
            L80 += COV[c][2][p][3]


    file.write(c+'\t'+str(COV[c][0])+'\t%.3f\t'
%(float(COV[c][0])*100/SIZE)+str(len(COV[c][1]))+'\t%0.1f\t%0.1f\
t%0.1f\t'
%(float(c30)*100/len(COV[c][1]),float(c50)*100/len(COV[c][1]),flo
at(c80)*100/len(COV[c][1]))+str(L80)+'\t%.3f\t'
%(float(L80)*100/SIZE)+str(N80)+'\n')


file.write('To-
tal\t'+str(TOT_cov)+'\t'+str(TOT_perc)+'\t'+str(TOT_el)+'\n')


file.close()
```

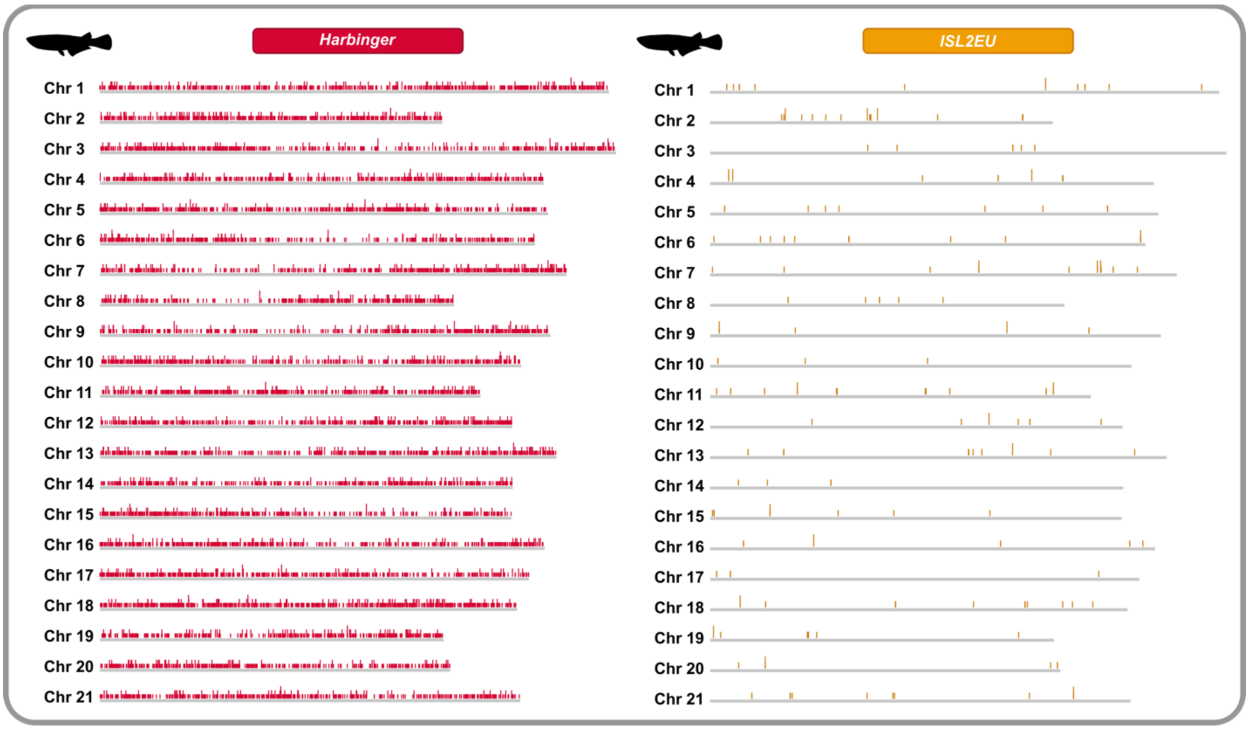

Figure S1. Distribution of *Harbinger* and *ISL2EU* transposons on medaka chromosomes. Each either red or orange colored bar corresponds to *Harbinger* or *ISL2EU* elements, respectively, at one genomic location. The height of each bar is proportional to the number of elements at a given position (smallest bars correspond to a single copy of an element).

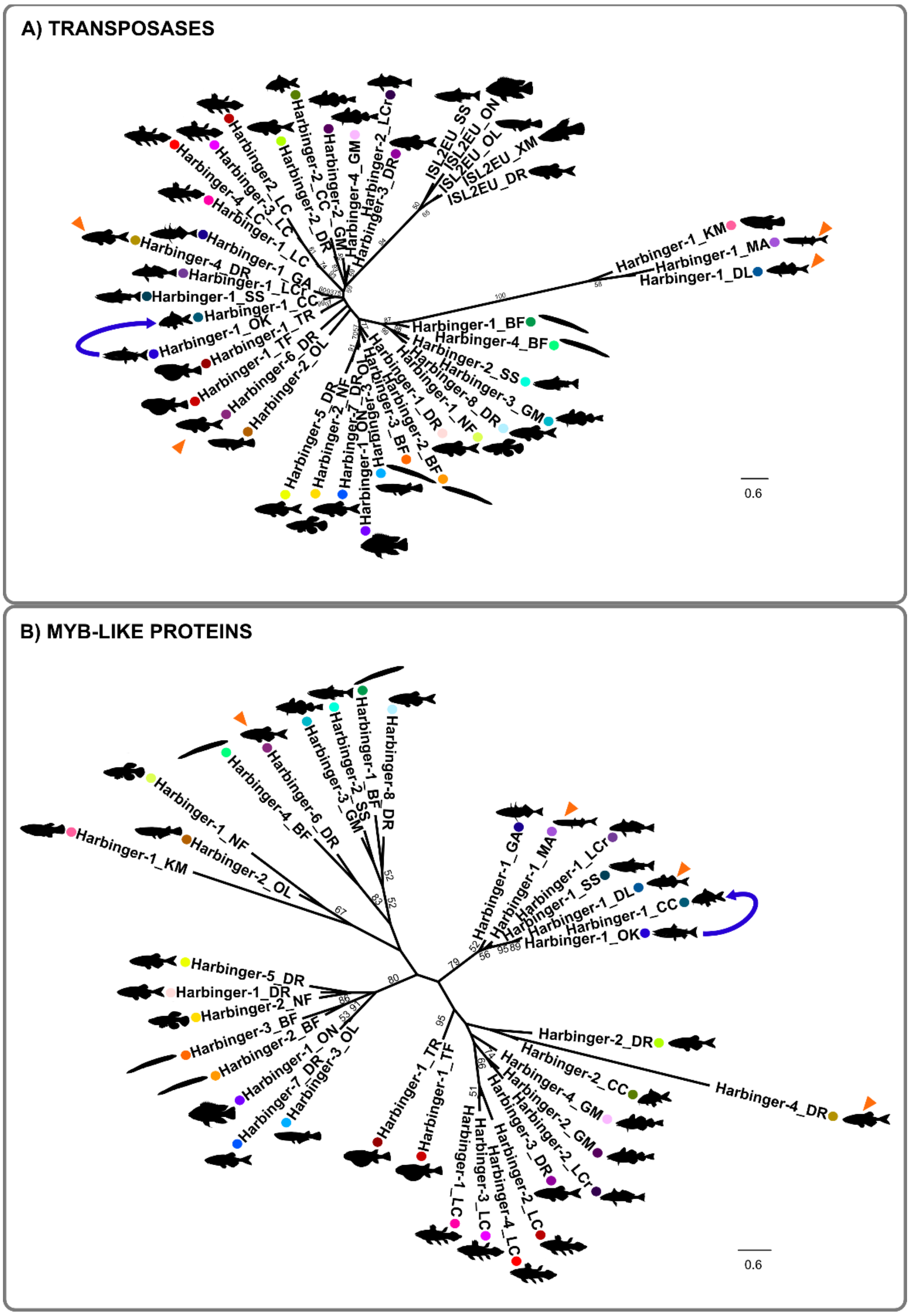

Figure S2. Phylogenetic relationships between *Harbinger* transposases (**A**) and Myb-like proteins (**B**) of different fish species. The tree was constructed using the Maximum Likelihood method [33]. Only bootstrap values higher than 50% are shown. Colored dots indicate correspondence between the transposases and Myb-like proteins from a same *Harbinger* element. The blue arrow shows a probable case of horizontal transfer. BF: *Branchiostoma floridae* – amphioxus, CC: *Cyprinus carpio* - carp, DL: *Dicentrarchus labrax* - bass, DR: *Danio rerio* – zebrafish, GA: *Gasterosteus aculeatus* – stickleback, GM: *Gadus morhua* – cod, KM: *Kryptolebias marmoratus* - killifish, LC: *Latimeria chalumnae* – coelacanth, LCr: *Larimichthys crocea* - croaker, MA: *Monopterus albus* - eel, NF: *Nothobranchius furzeri* – killifish, OK: *Oncorhynchus kisutch* - silver salmon, OL: *Oryzias latipes* – medaka, ON: *Oreochromis niloticus* – tilapia, SS: *Salmo salar* – salmon, TF: *Takifugu flavidus* – fugu, TR: *Takifugu rubripes* – fugu, XM: *Xiphophorus maculatus* – platyfish.

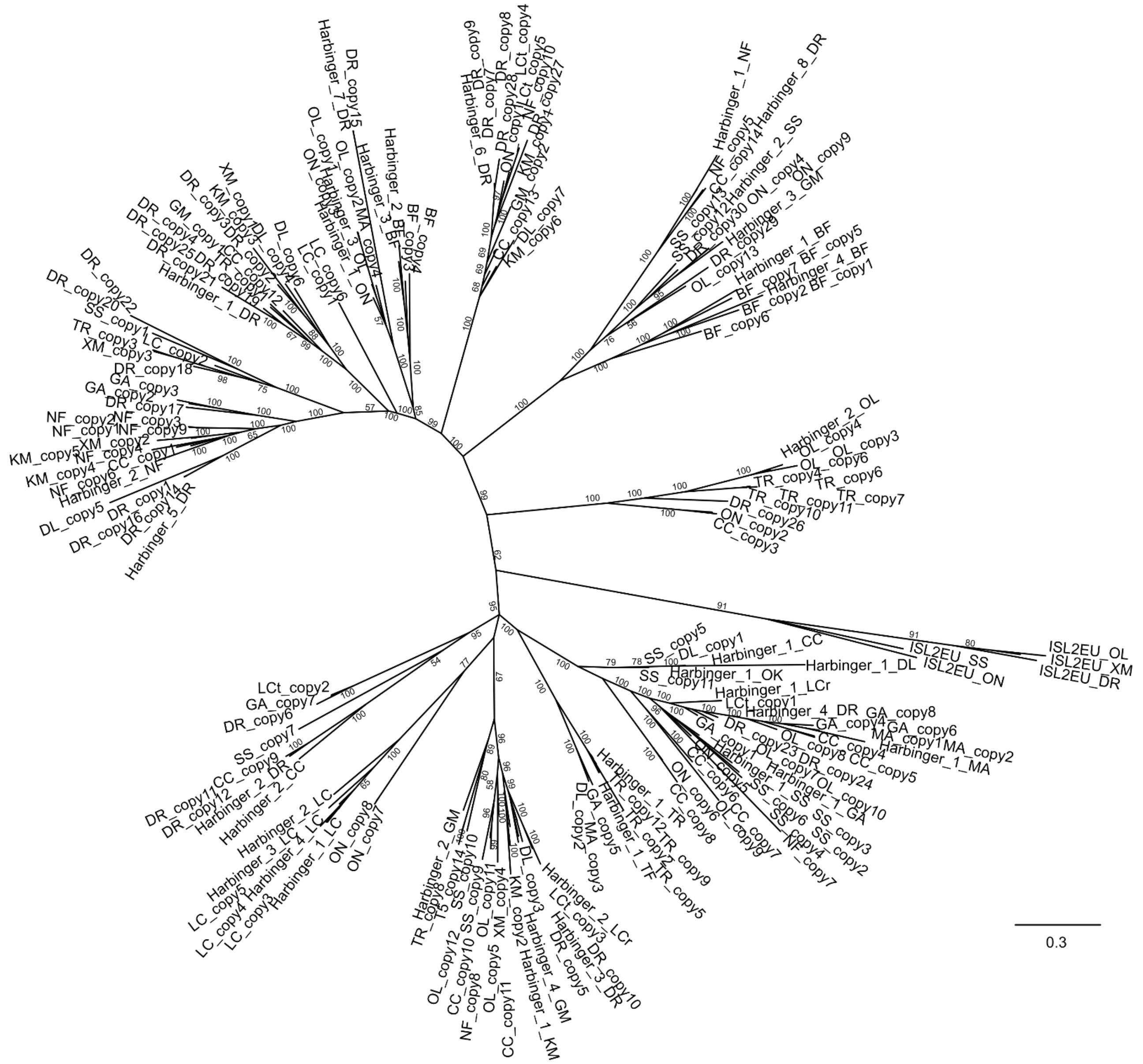

Figure S3. Phylogenetic relationships between *Harbinger* and *ISL2EU* transposase consensus and individual copy protein sequences from different fish species. Consensus sequences are indicated as "Harbinger_" and copies with "_copy". The tree was constructed using the Bayesian method [35]. BF: *Branchiostoma floridae* – amphioxus, CC: *Cyprinus carpio* - carp, DL: *Dicentrarchus labrax* - bass, DR: *Danio rerio* – zebrafish, GA: *Gasterosteus aculeatus* – stickleback, GM: *Gadus morhua* – cod, KM: *Kryptolebias marmoratus* - killifish, LC: *Latimeria chalumnae* – coelacanth, LCr: *Larimichthys crocea* - croaker, MA: *Monopterus albus* - eel, NF: *Nothobranchius furzeri* – killifish, OK: *Oncorhynchus kisutch* - silver salmon, OL: *Oryzias latipes* – medaka, ON: *Oreochromis niloticus* – tilapia, SS: *Salmo salar* – salmon, TF: *Takifugu flavidus* – fugu, TR: *Takifugu rubripes* – fugu, XM: *Xiphophorus maculatus* – platyfish.
